# Leptin-sensitive hypothalamic *Lepr+/Glp1r*+ neurons limit hyperphagia and weight gain in diet-induced obesity

**DOI:** 10.1038/s44318-026-00914-w

**Published:** 2026-10-02

**Authors:** Dylan M Belmont-Rausch, Benedicte Schultz Kapel, Abigail J Tomlinson, Chao Ding, Allison M Duensing, Bernd Coester, Cecilie Vad Mathiesen, Leonie Cabot, Jenny M Brown, Charlotte Høy Kruse, Shad Hassan, Elizabeth Heaton, Christoffer Clemmensen, Michael W Schwartz, Henning Fenselau, Martin G Myers, Tune H Pers

**Affiliations:** 1 Novo Nordisk Foundation Center for Basic Metabolic Research, University of Copenhagen, Copenhagen, Denmark; 2 Novo Nordisk Foundation Center for Genomic Mechanisms of Disease, Broad Institute of MIT and Harvard, Cambridge, MA USA; 3 Department of Molecular and Integrative Physiology, University of Michigan, Ann Arbor, MI USA; 4 Department of Internal Medicine, University of Michigan, Ann Arbor, MI USA; 5 Research Group Synaptic Transmission in Energy Homeostasis, Max Planck Institute for Metabolism Research, Cologne, Germany; 6 Excellence Cluster on Cellular Stress Responses in Aging Associated Diseases (CECAD) and Center of Molecular Medicine Cologne (CMMC), University of Cologne, Cologne, Germany; 7 Diabetes and Obesity Center of Excellence, Department of Medicine, University of Washington at South Lake Union, Seattle, WA USA; 8 Policlinic for Endocrinology, Diabetes, and Preventive Medicine (PEDP), University Hospital Cologne, Cologne, Germany; 9 Present Address: Research and Development, Novo Nordisk A/S, Måløv, Denmark

**Keywords:** Metabolism, Neuroscience

## Abstract

Despite loss of responsiveness to exogenous leptin in obesity, endogenous leptin continues to restrain feeding, although the neural substrates that remain sensitive and mediate this effect remain unknown. Combining spatial transcriptomics with single-nucleus RNA sequencing in mice with diet-induced obesity (DIO), we here show that while most hypothalamic leptin receptor (*Lepr*)-positive neurons minimally respond to elevated leptin, a single subpopulation defined by glucagon-like peptide-1 receptor (*Glp1r*) co-expression retains robust leptin sensitivity. These *Lepr*^*+*^*/Glp1r*^*+*^ neurons project onto and inhibit orexigenic *Agrp* neurons. *Lepr* deletion from *Lepr*^*+*^*/Glp1r*^*+*^ neurons blocks the anorectic effect of exogenous leptin, reinstates hyperphagic responses normally suppressed in DIO, amplifies the obesogenic response to palatable diet, and unexpectedly attenuates hypothalamic microglial activation – a hallmark of DIO previously attributed to diet rather than leptin signaling. Hence, preserved leptin responses by a single neuronal population in the hypothalamus limit hyperphagia and weight gain, among other responses, during obesity.

## Synopsis

Resistance to diet-induced obesity (DIO) is coupled to the rise of satiety hormone leptin but the cellular and molecular consequences in the central nervous system (CNS) remain unclear. Here, multi-omics profiling combined with genetics identify a key role for hypothalamic *Lepr*^*+*^*/Glp1r*^+^ neurons in controlling sensitivity of Agouti-related peptide (AgRP) neurons to orexigenic stimuli and systemic energy homeostasis.

- Spatial and single-cell transcriptomics of the hypothalamus of diet-induced obese mice identify *Lepr*^*+*^*/Glp1r*^+^ neurons as the principal leptin-responsive CNS cell population in obesity.
- *Lepr*^*+*^*/Glp1r*^+^ neurons respond to a broad spectrum of nutritional states and directly innervate orexigenic AgRP neurons in the arcuate nucleus (ARC).
- Chemogenetic activation of *Lepr*^*+*^*/Glp1r*^+^ neurons suppresses AgRP neuron-dependent feeding.
- Depletion of leptin signaling in *Lepr+/Glp1r+* neurons restores AgRP neuron responsiveness, prolongs diet-induced hyperphagia, and attenuates obesity-associated hypothalamic microgliosis.

## Abstract (web summary)

L*epr+/Glp1r*+ neurons in the CNS exhibit increased leptin responses and control AgRP neuron sensitivity to orexigenic stimuli and systemic regulation of energy homeostasis.

## Graphical abstract

**Figure:**
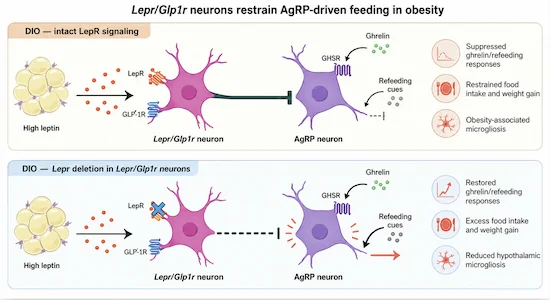

## Introduction

Leptin is an adipocyte-derived hormone that signals through its receptor (LepR; encoded by *Lepr*) to communicate the repletion of body energy stores. Hypothalamic leptin action plays a central role in the regulation of energy balance by suppressing food intake and increasing energy expenditure (Zhang et al, 1994; Pelleymounter et al, 1995; Montague et al, 1997; Friedman and Halaas, 1998). Obesity chronically elevates leptin and promotes a diminished anorectic response to exogenous leptin – a phenomenon broadly referred to as ‘leptin resistance’. In contrast, leptin deficiency causes obesity far beyond that observed in diet-induced obesity (DIO) (Ottaway et al, 2015) and interfering with leptin signaling exacerbates hyperphagia and weight gain in DIO animals (Pelleymounter et al, 1995; Friedman, 2016). It remains unclear whether specific neuronal populations respond to increased leptin to restrain hyperphagia and weight gain in DIO, however.

The canonical targets of leptin action in the arcuate nucleus (ARC)—leptin-inhibited orexigenic agouti-related peptide (*Agrp*)- and neuropeptide Y (*Npy*)-expressing neurons and leptin-activated anorexigenic pro-opiomelanocortin (*Pomc*)-expressing neurons (Pinto et al, 2004; Cowley et al, 2001)—both appear refractory to the direct effects of leptin in DIO (Tan et al, 2025; Enriori et al, 2007). Previous work has shown that at least some *Lepr*-positive cell populations in the dorsomedial hypothalamus (DMH) continue to respond to leptin during obesity, however (Simonds et al, 2014; Enriori et al, 2011). Moreover, even in the obese state, elevated leptin continues to restrain *Agrp* neuron activity through mechanisms that are not cell-intrinsic, suggesting that an upstream leptin-responsive population escapes the general loss of leptin sensitivity. Recent work identified a population of leptin-responsive DMH neurons that co-express glucagon-like peptide-1 receptor (*Glp1r*). While these GABAergic *Lepr/Glp1r* neurons project onto and inhibit *Agrp* neurons (Webster et al, 2024) and are required for the normal control of body weight by leptin in chow-fed mice (Xu et al, 2018), their function in obesity is unknown. We hypothesize that these neurons maintain their leptin responsiveness to tonically inhibit *Agrp* neurons and reduce hyperphagia in obesity.

Here, we use spatial transcriptomics, single-nucleus RNA sequencing (snRNA-seq), and molecular genetic mouse models to understand leptin action in DIO. We demonstrate that most *Lepr* neurons respond poorly to increased leptin in DIO, and that *Lepr/Glp1r* neurons represent the principal neural population that exhibits increased leptin responses in DIO. We show that these neurons project onto and control *Agrp* neuron gene expression and activity during DIO. Furthermore, removing leptin signaling from this single *Lepr/Glp1r* neuron population restores the sensitivity of *Agrp* neurons to orexigenic stimuli, amplifies the hyperphagic response to palatable diet, and attenuates hypothalamic microglial activation in DIO. These findings reframe our understanding of leptin action in obesity: rather than globally impairing leptin action, DIO progressively engages a specific neuronal population to limit hyperphagia and weight gain.

## Results

### A uniquely leptin-responsive hypothalamic neuronal population in obesity

**Figure 1:**
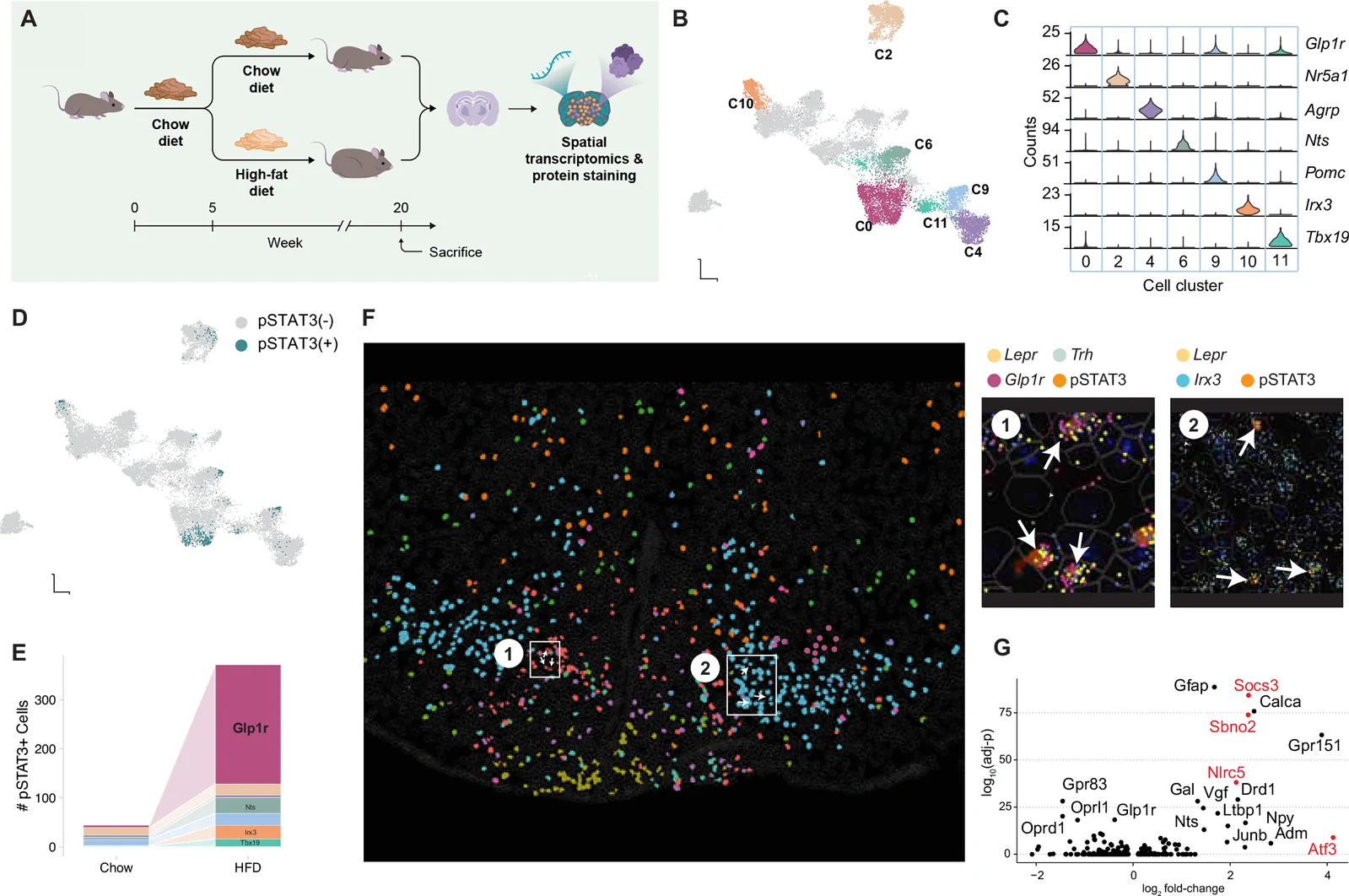
Elevated LepR signaling in hypothalamic *Lepr/Glp1r* neurons from DIO mice. (**A**) Schematic of the combined Xenium spatial transcriptomics and pSTAT3 immunofluorescence workflow used to quantify LepR signaling across hypothalamic cell types in chow‑fed and diet‑induced obese (DIO) mice. (**B**) UMAP embedding of neurons (17 populations; *n* = 6 mice total) across all spatially profiled sections, colored by transcriptionally defined Lepr cell clusters. (**C**) Marker gene expression across *Lepr* neuron subtypes (*n* = 6 mice), highlighting canonical cell-type markers. (**D**) UMAP embedding showing pSTAT3‑positive *Lepr* neurons (green). (**E**) Numbers of pSTAT3-positive cells across *Lepr* neuron subtypes in chow-fed and HFD-fed mice (*n* = 3 mice per group; 2–3 sections per mouse). DIO increased pSTAT3 positivity nearly tenfold overall (4.45 ± 0.65% vs. 0.47 ± 0.2%, *P* < 0.001; generalized linear mixed-effects model with binomial distribution, animal as random effect). This was driven predominantly by *Lepr/Glp1r* neurons, which showed a > 20-fold increase (21.1 ± 1.1% vs. 0.33 ± 0.2%, *P* < 1 × 10^−10^) and accounted for >60% of all pSTAT3-positive *Lepr* neurons in DIO. *P* values adjusted by Benjamini–Hochberg. (**F**) Representative distribution of each population of *Lepr* neurons across a single coronal section, color-coded as in (**B**, **C**). Right panels show representative Xenium and immunofluorescence images showing spatial colocalization of *Lepr, Glp1r, Trh*, and pSTAT3 in the boxed areas of the larger panel. (**G**) DIO‑induced transcriptional changes in *Lepr/Glp1r* neurons, including increased expression of canonical leptin target genes (*Socs3, Nlrc5, Sbno2, Atf3*; red) and immediate‑early genes (e.g., *Junb, Vgf*). Differentially expressed genes were identified from pseudobulk profiles using edgeR with Benjamini–Hochberg correction (adjusted *P* < 0.05; *n* = 3 chow, 3 DIO). Source data are available online for this figure.

**Figure EV1:**
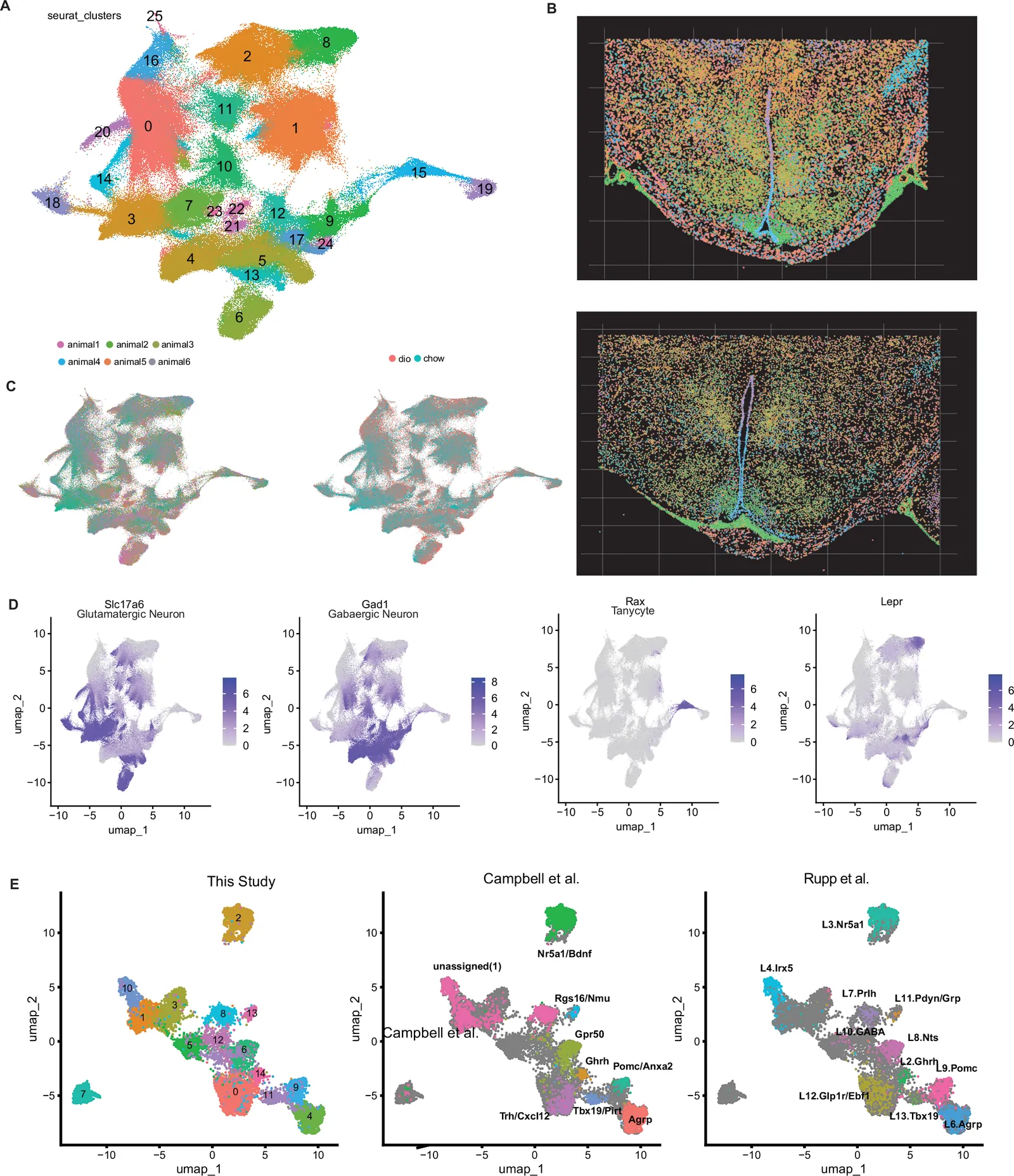
Characterization and annotation of hypothalamic cell populations by spatial transcriptomics. (**A**) UMAP embedding of all cells from Xenium in situ sequencing annotated by Seurat cluster numbers. Colors illustrate clusters. (**B**) Representative images from Xenium in spatial context. Colors match clusters in panel a. Top image from Xenium slide 1 and bottom image from Xenium slide 2. (**C**) UMAP embeddings of all cells from Xenium in situ sequencing annotated by animal (left panel) or by diet; 15 weeks HFD (DIO) and chow (right panel). (**D**) UMAP embeddings of all cells from Xenium in situ sequencing colored by expression of *Slc17a6* (glutamatergic neurons), *Gad1* (GABAergic neurons), *Rax* (tanycytes) and *Lepr*. (**E**) UMAP embedding of all neurons from Xenium in situ sequencing colored by Seurat cluster (left panel), by Campbell et al label transfer annotations (middle panel), and by Rupp et al label transfer annotations (right panel). Spatial cell clusters correspond to the following named populations: cluster 0, *Lepr/Glp1r*; cluster 2, *Nr5a1*; cluster 4, *Agrp*; cluster 6, *Nts*; cluster 9, *Pomc*; cluster 10, *Irx3*; cluster 11, *Tbx19*.

**Figure EV2:**
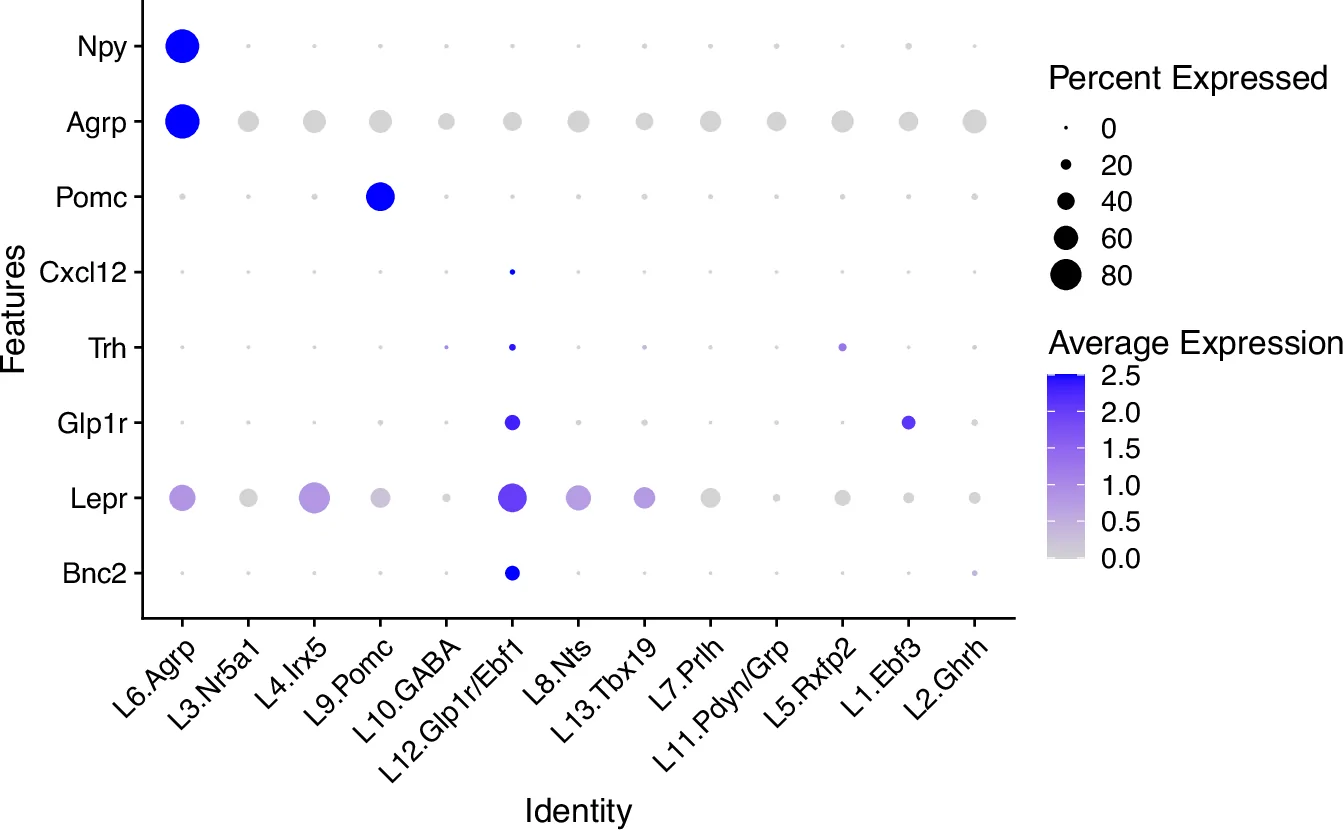
Expression of *Bnc2*, *Cxcl12*, *Glp1r*, *Lepr*, and *Trh* across mediobasal hypothalamic neuronal populations identified in Rupp et al. The canonical markers *Agrp*, *Pomc*, and *Npy* are shown for reference. The L12.Glp1r/Ebf1 cells correspond to the *Lepr/Glp1r* population. Dot size denotes the percentage of cells expressing each gene within a population, and color intensity reflects scaled average expression.

To identify hypothalamic neurons that remain responsive to leptin in obesity, we combined phosphorylated STAT3 (pSTAT3) immunoreactivity (pSTAT3-IR; a readout of cellular LepR signaling) (Vaisse et al, 1996; El-Haschimi et al, 2000), with spatial transcriptomics on the same tissue sections (Fig. 1A). Using a custom 300-gene panel, we profiled >312,000 hypothalamic cells from chow-fed lean and DIO mice (Table EV1; Fig. EV1A–C). We identified 17 distinct neuron populations (Figs. 1B,C and EV1D) that contained 7 previously described *Lepr* populations, including ARC *Agrp*-, *Pomc*-, and *Tbx19*-expressing neurons, as well as a large GABAergic *Glp1r*-expressing population that spans the ventral DMH and caudal ARC. Label transfer confirmed correspondence of this latter population with previously transcriptionally described *Lepr/Glp1r/Bnc2/Trh/Cxcl12* co-expressing hypothalamic neurons (Figs. EV1E and EV2) (Rupp et al, 2023; Webster et al, 2024; Tan et al, 2024).

**Figure EV3:**
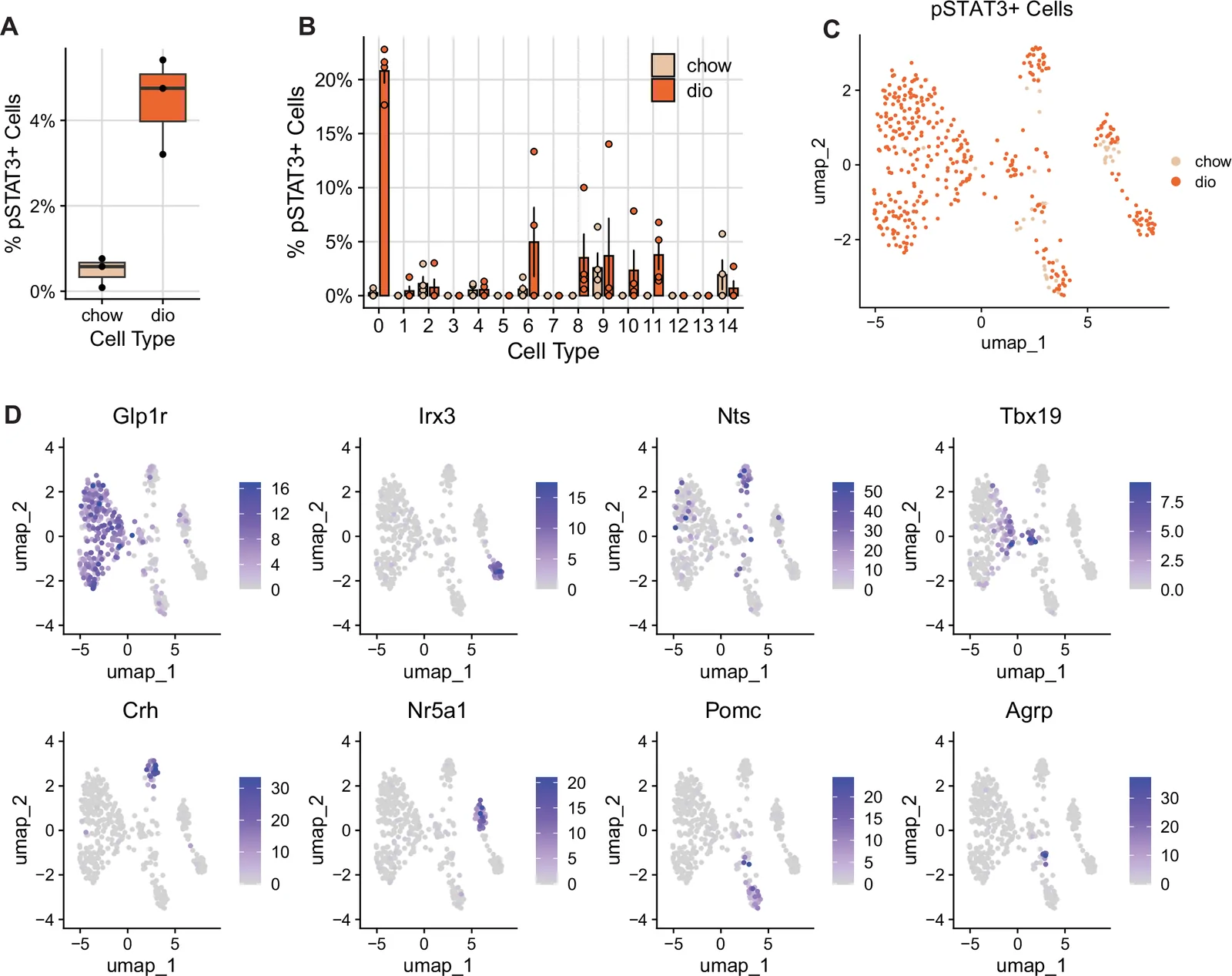
Lepr/Glp1r neurons are the principal pSTAT3-immunoreactive population in diet-induced obesity. (**A**) Quantification of pSTAT3-immunoreactive (pSTAT3-IR) cells in hypothalami of chow-fed and high-fat diet (HFD)-fed mice (*n* = 3 mice per group; 2–3 sections per mouse). (**B**) Distribution of pSTAT3-IR cells across identified neuronal clusters from Fig. 1 in HFD-fed (red) and chow-fed (light orange) mice. (*n* = 3 mice per group). Data are mean ± SEM. (**C**) UMAP visualization of Xenium in situ sequencing data showing pSTAT3-IR cells, colored by diet condition (HFD: red; chow: light orange). (**D**) UMAP visualization showing expression patterns of key neuronal marker genes (*Glp1r, Irx3, Nts, Tbx19, Crh, Nr5a1, Pomc*, and *Agrp*) in pSTAT3-IR cells.

We detected pSTAT3-IR in the seven *Lepr* neuron populations in the murine MBH (“Methods”, Fig. 1D). DIO increased the overall fraction of pSTAT3-IR *Lepr* neurons nearly 10-fold relative to chow controls (4.45 ± 0.65% *vs*. 0.47 ± 0.2%, *P* < 0.001, *n* = 3 mice/group; Figs. 1E,F and EV3A,B). This pSTAT3-IR increased only slightly in canonical ARC leptin targets (i.e., *Agrp* or *Pomc* neurons), however, while it increased >20-fold in *Lepr/Glp1r* neurons, which accounted for more than 60% of all pSTAT3-positive neurons in DIO (Figs. 1E and EV3C,D).

DIO increased the expression of canonical LepR transcriptional targets (e.g., *Socs3, Nlrc5, Sbno2*) in *Lepr/Glp1r* neurons. Notably, the strong induction of *Socs3*—a well-characterized negative feedback regulator of LepR signaling—failed to terminate the transcriptional response of these cells to leptin, indicating that *Lepr/Glp1r* neurons remain leptin sensitive despite ongoing feedback inhibition. Immediate early genes associated with neuronal activation (*Junb, Vgf*) were also increased by DIO in these neurons, suggesting their increased activity in obesity (Fig. 1G). While a couple of other *Lepr* populations showed evidence of increased leptin responses (increased pSTAT3) in DIO, the proportion of *Lepr/Glp1r* neurons that responded and the magnitude of their response identified these neurons the principal leptin-responsive neuron population.

### *Lepr/Glp1r* neurons uniquely respond to a spectrum of nutritional states

**Figure 2:**
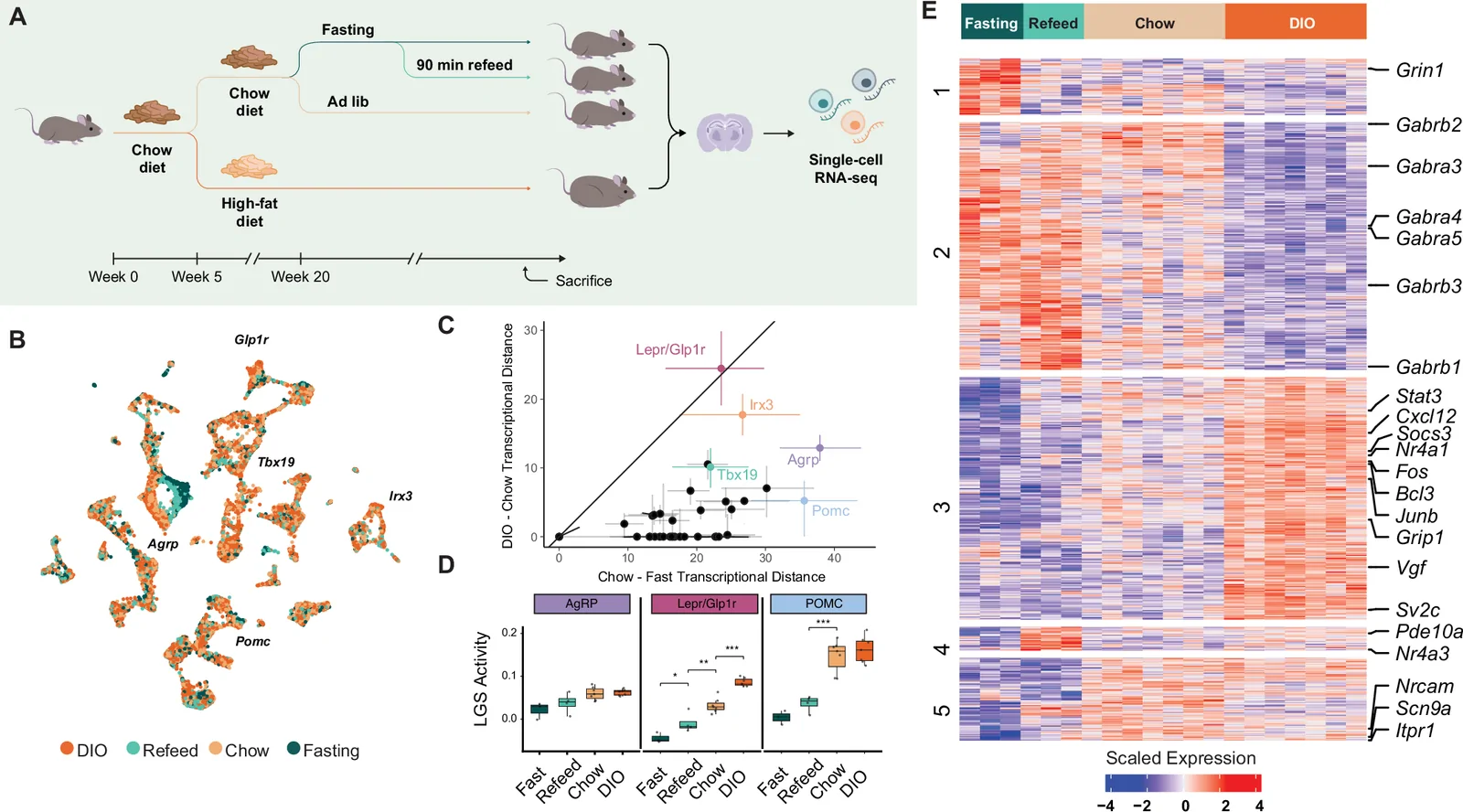
Uniquely elevated leptin signaling in *Lepr/Glp1r* neurons in DIO mice. (**A**) Experimental design for snRNA-seq profiling of hypothalamic neurons across metabolic states: chow-fed (Chow; *n* = 7), overnight-fasted (Fasting; *n* = 3), fasted and refed (90 min) (Refeed; *n* = 4), and DIO mice (DIO; *n* = 8). (**B**) UMAP embedding of MBH neurons (> 40,000 nuclei; mean 12,401 transcripts per nucleus), with *Lepr*-expressing neurons labelled by subtype. (**C**) Effects of nutritional manipulations on the total transcriptome of each neuronal population (chow *n* = 7, fasted *n* = 3, refed *n* = 4, DIO *n* = 8 mice). Expression distance estimate in response to fasting (*x* axis) and DIO (*y* axis). Data for *Lepr* neurons is highlighted in cell-type specific colors. Solid diagonal line indicates matched effects between perturbations. Error bars denote the 95% confidence interval of the expression-distance estimate (scDist). (**D**) Leptin gene signature (LGS) expression in for each nutritional manipulations across the indicated subsets of *Lepr* neurons; DIO (red, *n* = 8), refeed (light green, *n* = 4), chow (yellow, *n* = 7), fasting (dark green, *n* = 3). Box plots show the median (centre line), the 25th and 75th percentiles (box bounds) and whiskers extending to the most extreme value within 1.5× the interquartile range. Differences across nutritional states were tested with linear mixed-effects models with pairwise contrasts via estimated marginal means (Benjamini–Hochberg adjusted); +*P* < 0.1, *P < 0.05, **P < 0.01, ***P < 0.001. Exact adjusted *P* values for the annotated comparisons: Lepr/Glp1r neurons Fasting vs Refeed *P* = 0.084, Refeed vs Chow *P* = 2.1 × 10^–3^, Chow vs HFD *P* = 2.8 × 10^–4^; POMC neurons Refeed vs Chow *P* = 7.0 × 10^–4^; no comparison reached significance in AgRP neurons. All pairwise contrasts for all subtypes are provided in the source data. (**E**) Hierarchical clustering and grouping of differentially expressed genes identified in *Lepr/Glp1r* neurons. Genes associated with LGS, neuronal activity, and GABA receptor subunits are highlighted. Source data are available online for this figure.

**Figure EV4:**
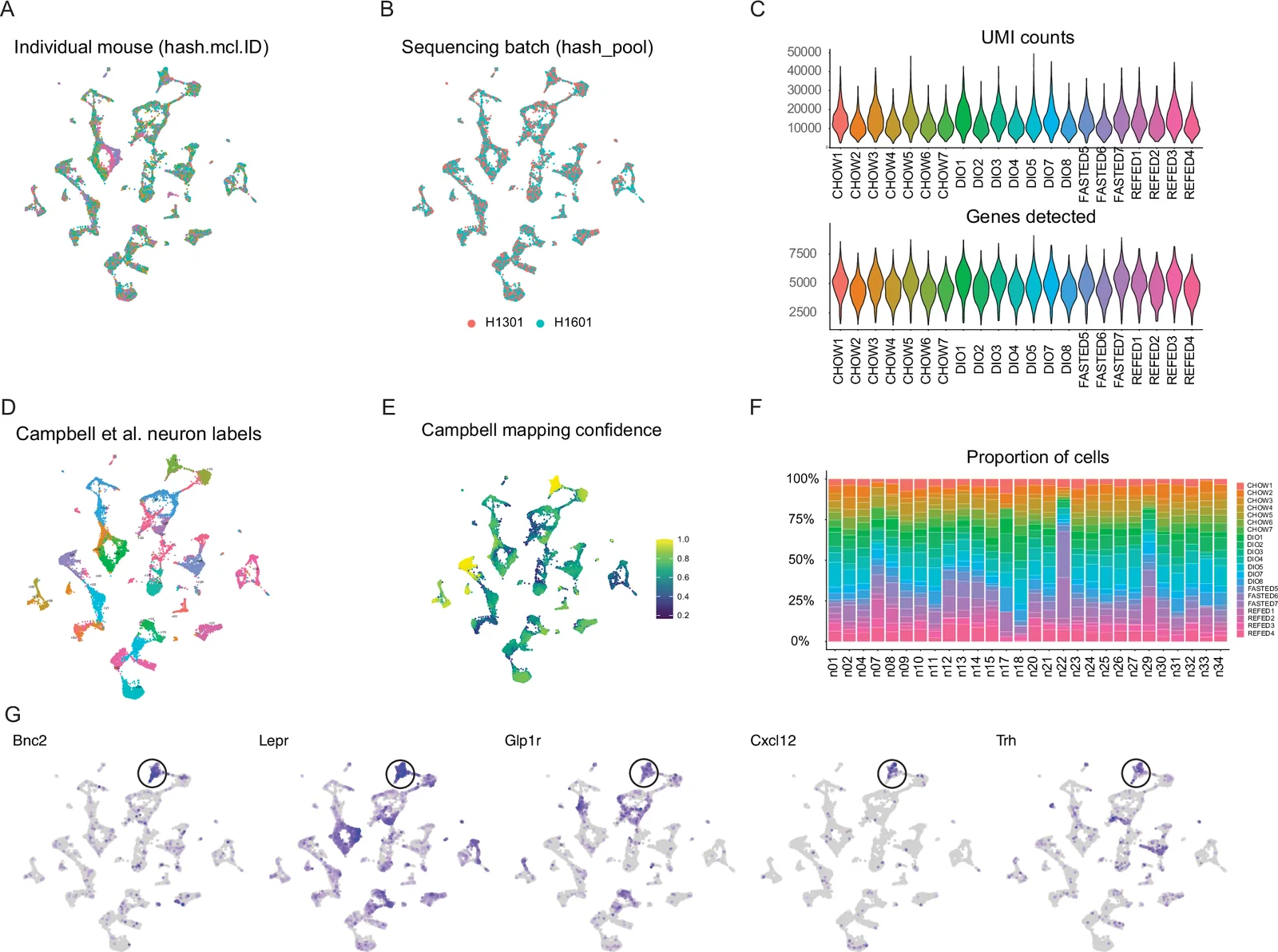
Quality control and cell type annotation of mediobasal hypothalamic snRNA-seq data. (**A**, **B**) UMAP embedding of mediobasal hypothalamic neurons colored by individual mouse (**A**) and sequencing batch (**B**). (**C**) Distribution of UMI counts (top) and genes detected (bottom) per cell across all samples (chow *n* = 7, DIO *n* = 8, fasted *n* = 3, refed *n* = 4). (**D**) UMAP colored by predicted Campbell et al neuron subtype. (**E**) Mapping confidence scores, with highest confidence in the *Lepr/Glp1r* population. (**F**) Proportion of cells assigned to each neuronal cluster (n01-n34) across individual samples, showing consistent cluster composition across mice and conditions. (**G**) UMAP colored by the expression of key markers genes of *Lepr/Glp1r* neurons to demonstrate overlap with previously described populations.

The strong pSTAT3 response of *Lepr/Glp1r* neurons to DIO prompted us to ask whether these neurons also respond to leptin across a broader range of nutritional states. We therefore used snRNA-seq to profile more than 40,000 hypothalamic neuron transcriptomes across four conditions: fasting, refeeding, ad libitum chow-fed, and DIO (Fig. 2A,B; *n* = 4–8 mice per group; Fig. EV4A–F). To identify which populations were most sensitive to these nutritional perturbations, we computed a transcriptional distance metric (*scDist*) that quantifies the shift in overall gene expression profile for each cell type relative to chow-fed baseline. Most populations responded robustly to fasting, consistent with the established sensitivity of hypothalamic *Lepr* neurons to energy deficit. In contrast, only *Lepr/Glp1r* neurons showed comparably strong responses to both fasting and DIO (Fig. 2C). Cross-referencing our *Lepr/Glp1r* population with independently generated hypothalamic single-cell atlases confirmed that these neurons correspond to the DMH *Lepr*/*Glp1r*-co-expressing neurons recently shown to impact leptin and GLP-1 receptor agonist efficacy on feeding and body weight (Fig. EV4G) (Webster et al, 2024; Kim et al, 2024; Aitken et al, 2024; Tan et al, 2024), though none of these studies specifically examined the regulation of this population in DIO.

**Figure EV5:**
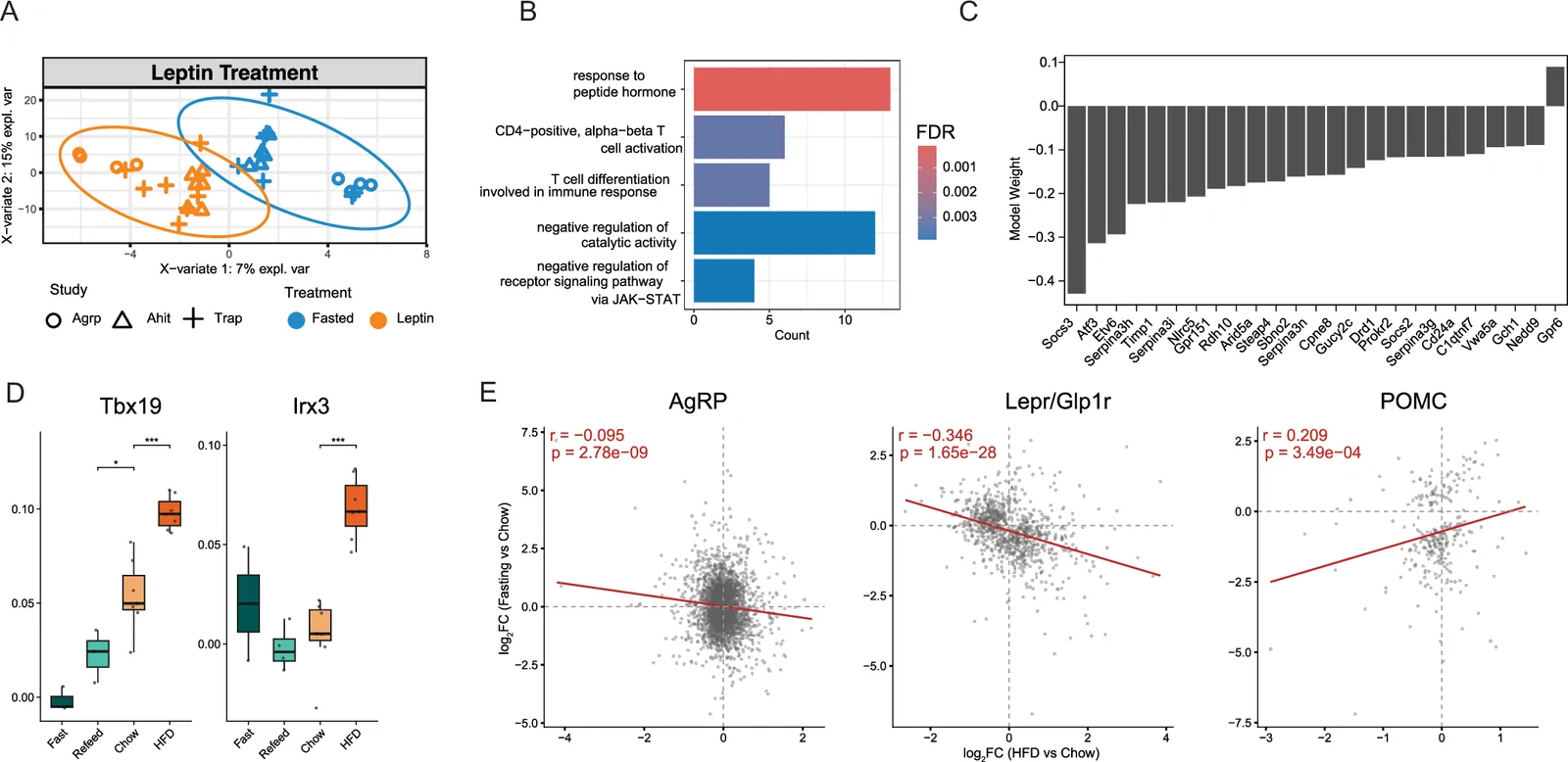
Derivation and validation of the leptin gene signature (LGS) across neuron populations and nutritional states. (**A**) MINT sPLS-DA projection of three independent leptin treatment transcriptomic datasets used to derive the leptin gene signature (LGS). Fasted/control (blue) and leptin-treated (orange) samples separate along the first two components. (**B**) Gene ontology enrichment of LGS genes, highlighting response to peptide hormone and JAK-STAT signaling among the top terms. (**C**) Model weights for individual LGS genes. (**D**) LGS expression across nutritional states in *Tbx19* and Irx3 neurons (*P < 0.05, **P < 0.01, ***P < 0.001; chow *n* = 7, fasted *n* = 3, refed *n* = 4, DIO *n* = 8). Box plots show the median (centre line), the 25th and 75th percentiles (box bounds) and whiskers extending to the most extreme value within 1.5× the interquartile range; points beyond the whiskers are plotted individually. (**E**) Correlation between fasting-induced (*y* axis) and DIO-induced (*x* axis) log₂ fold changes in *Agrp*, *Lepr/Glp1r*, and *Pomc* neurons.

Because these state-dependent responses could reflect the effects of multiple features of altered energy balance other than leptin, we next asked which component was attributable to LepR signaling. To isolate the component of transcriptional change attributable to direct leptin signaling in each cell type, we derived a leptin gene signature (LGS), a composite score reflecting the coordinated expression of genes consistently regulated by leptin across three independent treatment datasets (Allison et al, 2018; Inoue et al, 2019; Heyward et al, 2024). This signature includes canonical LepR targets such as *Socs3, Etv6*, and *Atf3* (among others) (Fig. EV5A–C; Tables EV2 and 3).

Consistent with our pSTAT3 finding, DIO significantly increased LGS expression in *Lepr/Glp1r* (and to some extent *Tbx19* and *Irx3/5* neurons), but not other cell types. Fasting reduced LGS expression in *Lepr/Glp1r, Pomc*, and *Tbx19* neurons (Figs. 2D and EV5D). Thus, among the populations examined, *Lepr/Glp1r* neurons showed the clearest bidirectional leptin sensing response, strongly increasing in DIO and decreasing during fasting. Interestingly, *Agrp* neurons displayed minimal LGS changes across conditions, despite their strong overall transcriptional differences between fasting and chow-fed conditions. This finding is potentially consistent with a predominantly indirect effect of leptin on gene expression in *Agrp* neurons. Note that no *Lepr* neuron population exhibited decreased LGS expression in DIO compared to chow-fed animals, however. Thus, DIO does not abrogate LepR signaling, but rather most *Lepr* cell types fail to increase LepR signaling in obesity.

This bidirectionality of the *Lepr/Glp1r* neuron transcriptional response extended beyond canonical leptin targets. In these cells, all genes that increased during fasting tended to decrease in DIO, and vice versa – a pattern of mirror-image regulation not seen in *Agrp* or *Pomc* neurons (Pearson’s *r* = −0.35, *P* = 1.65 × 10^–28^). This bidirectionality suggested that *Lepr/Glp1r* neurons actively track nutritional state across both energy deficit and surplus (Fig. EV5E).

Clustering of the 963 diet-regulated genes in *Lepr/Glp1r* neurons revealed a coherent transcriptional program (Fig. 2E). Genes associated with neuronal activation (*Fos, Vgf, Sv2c*) and cytokine signaling (*Stat3, Cxcl12, Socs3*) increased progressively from fasting through chow to DIO. In contrast, GABA receptor subunits showed the opposite pattern, including downregulation of several GABAA subunits (*Gabra3, Gabra4, Gabra5*) and all three GABAB subunits (*Gabrb1–b3*) from DIO through chow to fasting (Tables EV4–6). These data suggest that *Lepr/Glp1r* neurons become more active and less sensitive to inhibitory input with increased adiposity – a transcriptional profile consistent with their enhanced output onto downstream circuits.

**Figure 3:**
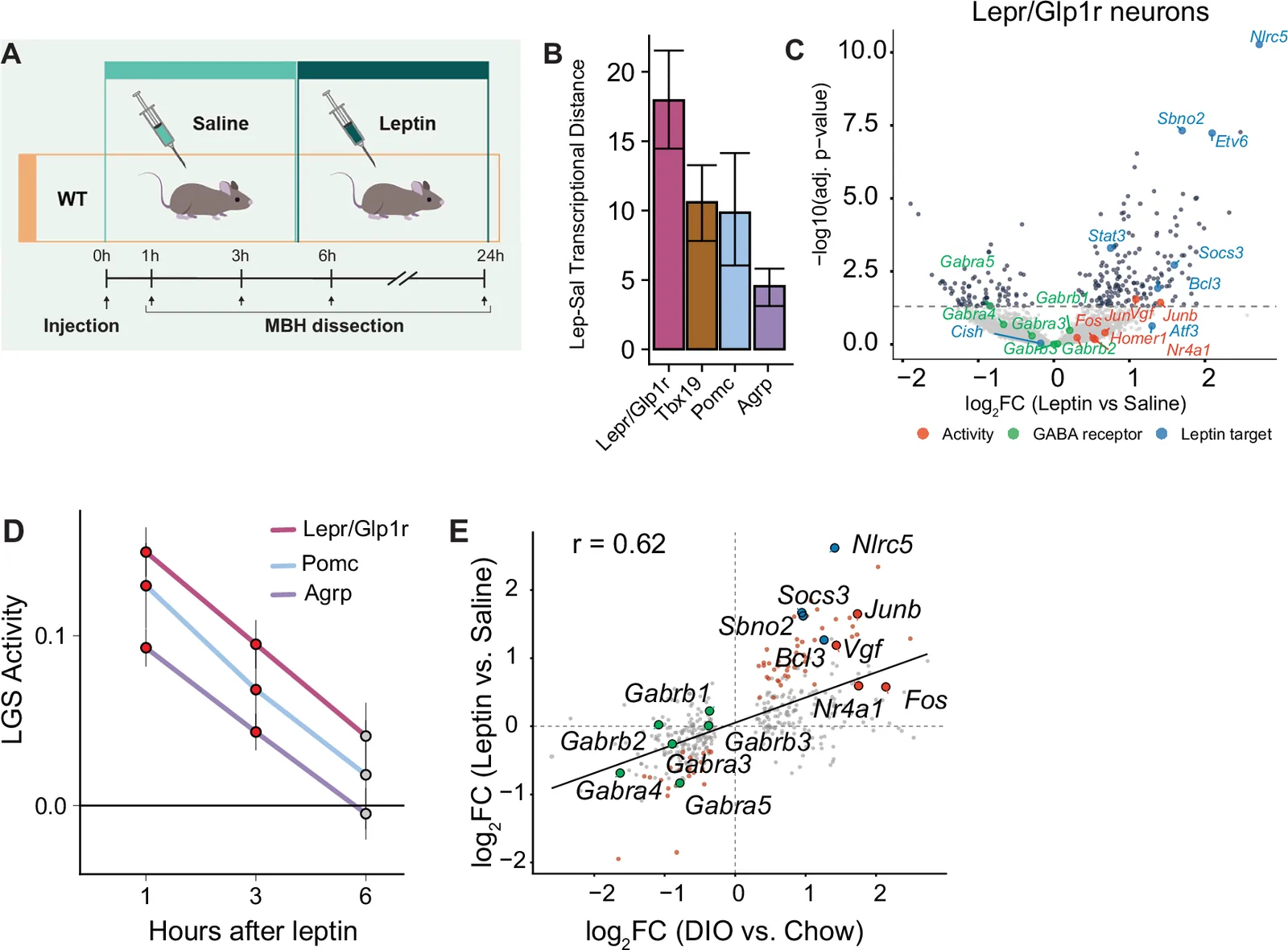
Acute leptin administration recapitulates the DIO transcriptional program in *Lepr/Glp1r* neurons. (**A**) Experimental design of wild-type (WT) mice treated with 3 mg/kg of leptin or saline and sacrificed 1, 3, 6, or 24 h after injection. MBH nuclei were collected for snRNA-seq. (**B**) Transcriptional distance between cells from leptin-treated and control mice (*n* = 4–6 per timepoint per group). Error bars denote the 95% confidence interval of the expression-distance estimate (scDist). (**C**) volcano plot showing leptin-regulated genes in *Lepr/Glp1r* neurons (*n* = 4–6 per timepoint per group), highlighting specific genes of interest. Differentially expressed genes were identified from pseudobulk profiles using edgeR, Benjamini–Hochberg adjusted *P* < 0.05. (**D**) LGS changes across *Lepr/Glp1r*, *Pomc* and *Agrp* neurons (*n* = 4–6 per timepoint per group). Data are mean ± SEM. (**E**) Overlap between genes induced by acute leptin treatment and those upregulated in DIO in *Lepr/Glp1r* neurons (93 shared genes; odds ratio = 185.3, *P* = 6.28 × 10^–24^, Fisher’s exact test), indicating that direct leptin action recapitulates a substantial portion of the DIO transcriptional program. Source data are available online for this figure.

**Figure EV6:**
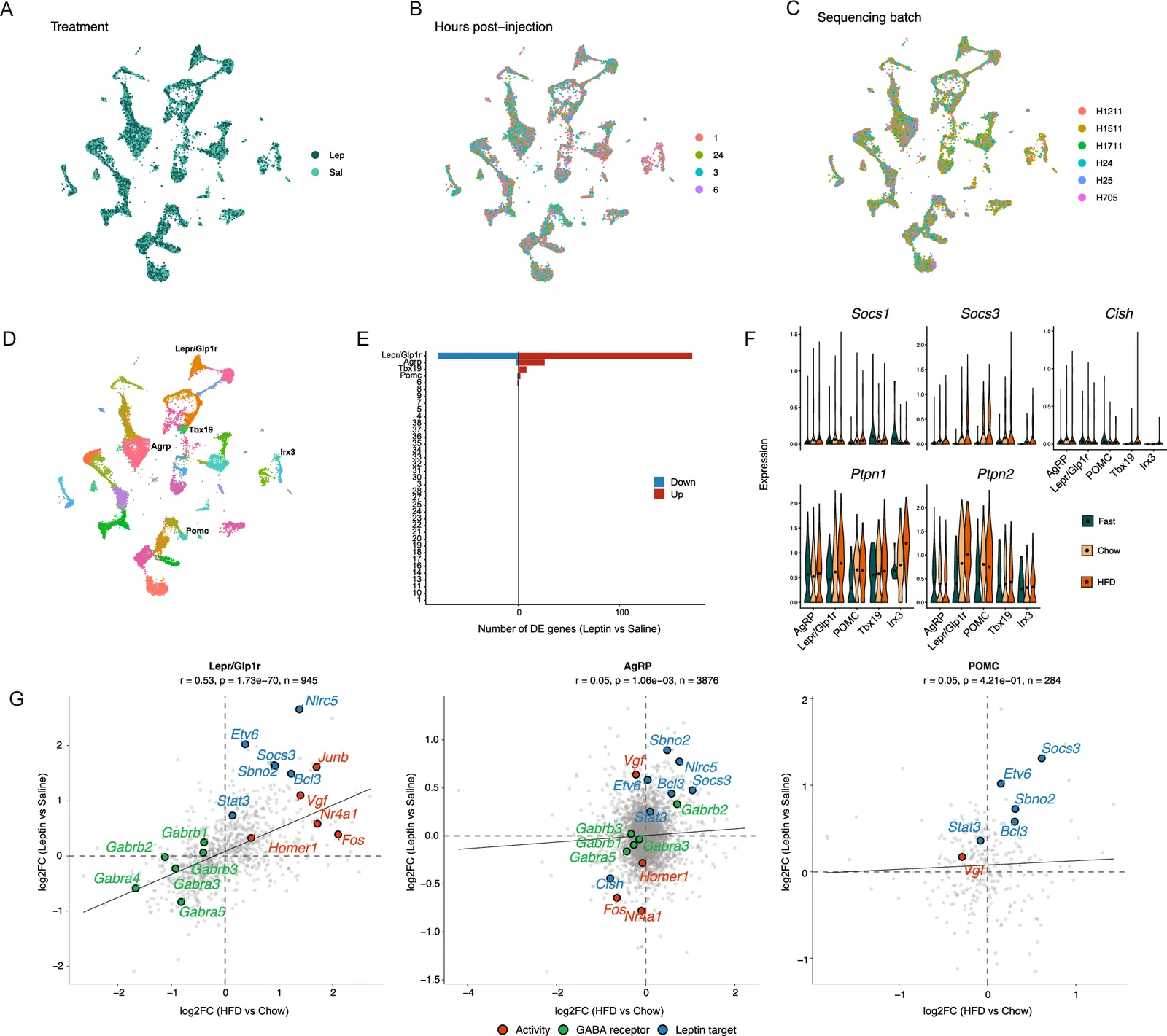
Acute leptin treatment selectively engages Lepr/Glp1r neurons and recapitulates the DIO transcriptional program. (**A**–**C**) UMAP embedding of mediobasal hypothalamic neurons from lean mice treated with leptin (3 mg/kg) or saline and harvested at 1, 3, 6, or 24 h post-injection (*n* = 4–6 per timepoint per group), colored by treatment (**A**), hours post-injection (**B**), and sequencing batch (**C**). (**D**) UMAP colored by neuronal cell type identity, with key *Lepr*-expressing populations labeled. (**E**) Number of differentially expressed genes (leptin vs saline; adjusted *P* < 0.05) per neuronal population. Red, upregulated; blue, downregulated. (**F**) Violin plots showing single-cell expression of JAK-STAT feedback inhibitors (*Socs1, Socs3, Cish, Ptpn1, Ptpn2*) across five *Lepr*-positive neuronal populations in fasted (teal), chow-fed (light orange), and DIO (red) conditions. Black dots indicate mean expression per group. (**G**) Scatter plots showing the relationship between HFD fold change (*x* axis) and leptin IP fold change (*y* axis) for all genes significantly regulated by diet (*F* test, FDR < 0.05) in *Lepr/Glp1r* (left), *Agrp* (center), and *Pomc* (right) neurons. Each point represents a gene; Pearson correlation coefficient and *P* value are indicated.

To test whether leptin itself sufficed to induce this nutritional sensing program, we profiled single nuclei from lean mice treated with vehicle or leptin (3 mg/kg, IP) and harvested the MBH at 1, 3, 6, or 24 h post-injection (*n* = 4–6 per timepoint per group; >100,000 nuclei total; Figs. 3A and EV6A–D). As with nutritional manipulations, *Lepr/Glp1r* neurons mounted the strongest response to leptin of any cell population (Figs. 3C,D and EV6E). Strikingly, leptin-regulated 251 genes in *Lepr/Glp1r* neurons but only 28 in *Agrp* neurons (Table EV7; Fig. EV6E). Together with the minimal overlap of the leptin-regulated genes from *Agrp* and *Lepr/Glp1r* neurons and the negligible LGS response in *Agrp* neurons, this near-complete absence of an overall transcriptional response to exogenous leptin in *Agrp* neurons (despite their expression of *Lepr*) is consistent with a predominantly indirect effect of leptin on *Agrp* neurons in vivo (i.e., suggesting that leptin mainly modulates *Agrp* neurons via upstream populations such as *Lepr/Glp1r* neurons). Genes induced by exogenous leptin significantly overlapped with those upregulated in DIO in especially *Lepr/Glp1r* neurons (Figs. 3E and EV6G), indicating that exogenous leptin administration can recapitulate a substantial component of the *Lepr/Glp1r* transcriptional program observed in DIO.

### *Lepr/Glp1r* neurons in the ARC and DMH directly innervate *Agrp* neurons

**Figure EV7:**
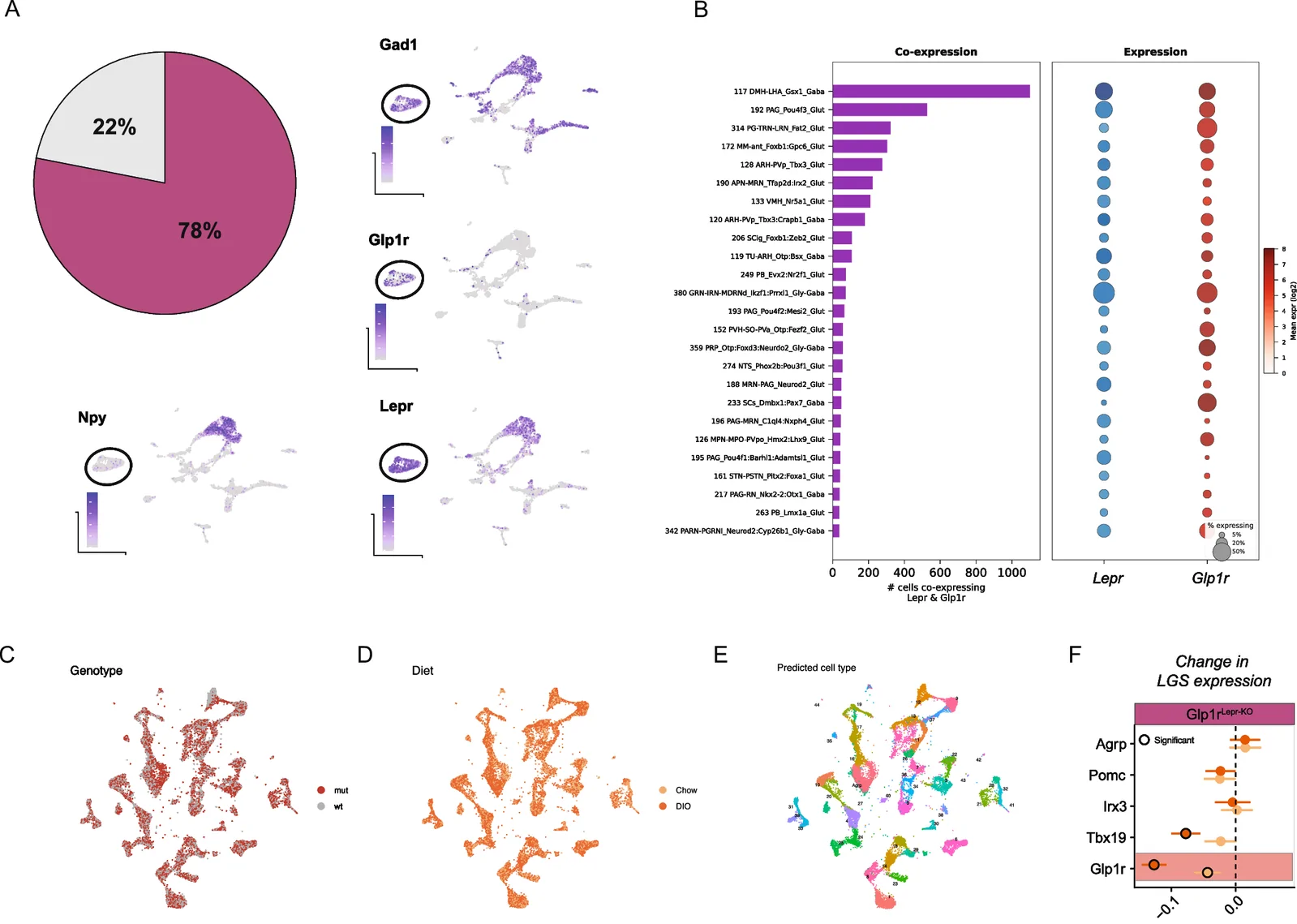
Lepr/Glp1r neurons dominate inhibitory LepR input to AgRP neurons and show cell-autonomous loss of leptin signaling in Glp1rLepr-KO mice. (**A**) *Lepr/Glp1r* neurons constitute ~78% of all *Lepr*-expressing GABAergic (*Gad1* + ) input to AgRP (*Npy* + ) neurons, based on reanalysis of published rabies-traced AgRP neuron afferents. (**B**) Whole-brain co-expression of Lepr and Glp1r in the Allen Brain Cell Atlas. Left, number of cells co-expressing both transcripts in the 25 cell subclasses with the highest co-expression. Right, dot plot of Lepr and Glp1r in the same subclasses: dot size denotes the percentage of cells expressing the gene and colour the mean log₂-normalised expression. (**C**–**E**) UMAP embedding of mediobasal hypothalamic neurons from Lepr^Glp1r^KO and control mice, colored by (**C**) genotype, (**D**) diet (chow vs DIO; *n* = 6–7 per group), and (**E**) predicted cell type identity (41 clusters) based on label transfer from the nutritional perturbation reference atlas. (**F**) Change in leptin gene signature (LGS) expression in Lepr^Glp1r^KO relative to control mice (*n* = 6–7 per group) across *Lepr* neuron subtypes (open circles denote *P* < 0.05; linear mixed-effects model). Error bars denote (95% CI).

**Figure 4:**
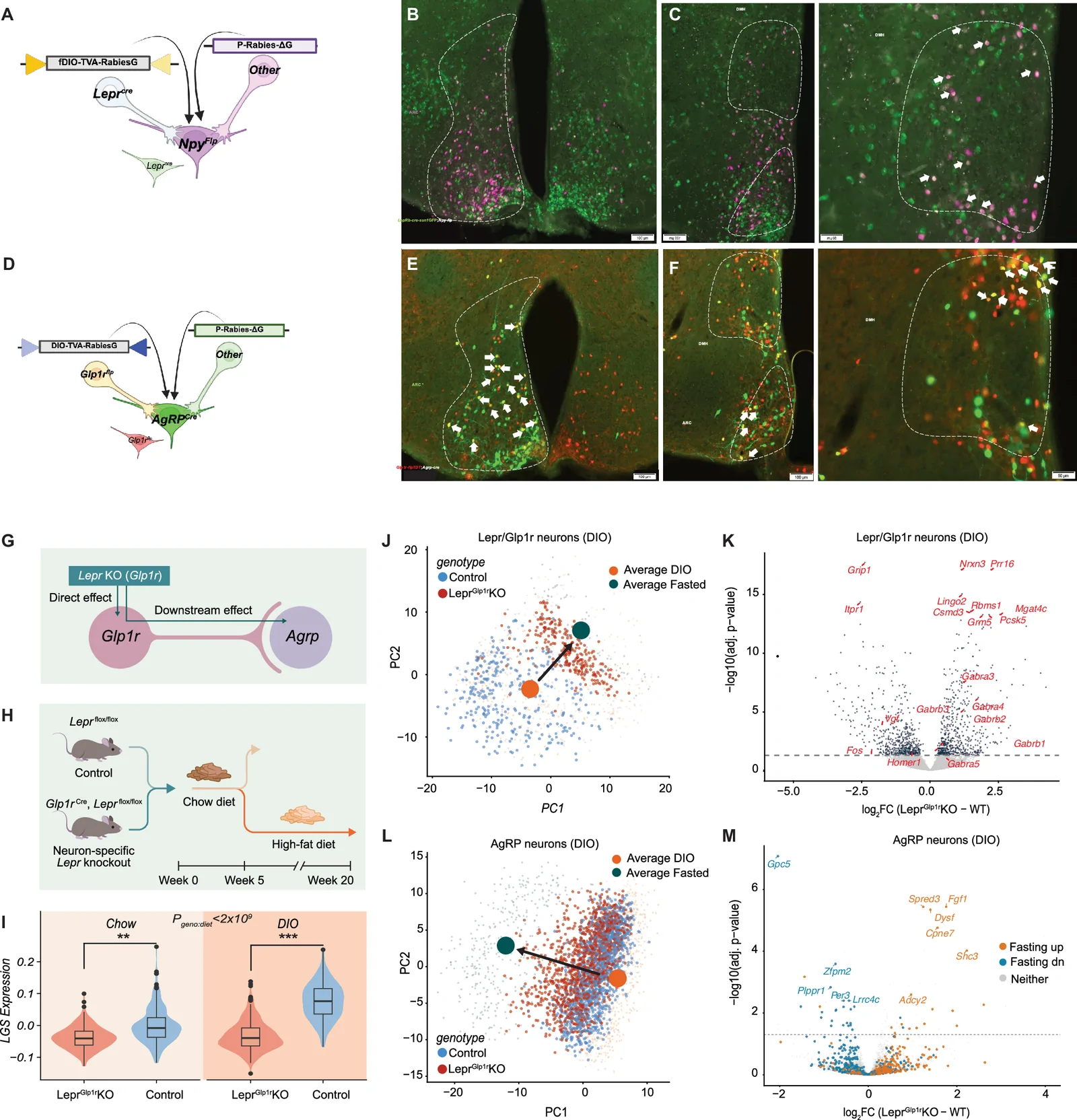
*Lepr/Glp1r* neurons project to *Agrp* neurons and LepR signaling via *Lepr/Glp1r* neurons controls *Agrp* neuron gene expression in obesity. (**A**) Schematic of retrograde rabies tracing from ARC *Npy* neurons in *Lepr*^*Cre-sun1Gfp*^*;Npy*^*Flp*^ mice. Starter cells (ARC) express TVA and rabies-G via *Npy*^*Flp*^; rabies-labeled cells are magenta, *Lepr*-expressing cells green, and co-labeled cells (*Lepr*-expressing afferents) show both signals. (**B**, **C**) Representative images of tissue sections from experiments as in (**A**). *Indicates viral hit site. Afferent DMH *Lepr* cells indicated by white arrows. (**D**) Schematic of retrograde rabies tracing from *Agrp* neurons in *Agrp*^*Cre;*^*Glp1r*^*Flp-TDT*^ mice. Rabies-labeled cells are green, *Glp1r*-expressing cells red, and co-labeled cells (*Glp1r*-expressing afferents) show both signals. (**E**, **F**) Representative images of tissue sections from experiments as in (**D**); *indicates viral hit site. Rabies-labeled *Glp1r* afferent cells indicated with white arrows. Scale bars: 200 µm (main images), 100 µm (insets). (**G**, **H**) Experimental paradigm: *Lepr* was ablated in *Glp1r* expressing neurons and animals were fed chow diet (*Agrp*, *n* = 7; *Glp1r*, *n* = 6 *Lepr*^*KO*^ and *n* = 6 control) and either sacrificed at 4–5 weeks of age switched onto a HFD for 15 weeks (*Agrp* *n* = 2; *Glp1r*, *n* = 7 *Lepr*^*KO*^ and *n* = 6 control) until sacrifice. Mediobasal hypothalami were collected for snRNA-seq. (**I**) Leptin gene signature (LGS) expression in *Lepr/Glp1r* neurons from for lean (Chow: *n* = 6 Lepr^Glp1r^KO, 6 control) or DIO mice (DIO: *n* = 7 Lepr^Glp1r^KO, 6 control). LGS was significantly reduced in KO neurons (β = −0.087, *P *< 1.0 × 10⁻⁹; linear mixed-effects model), with a significant genotype × diet interaction (*P* = 2 × 10⁻⁹). Box plots show the median (centre line), the 25th and 75th percentiles (box bounds) and whiskers extending to the most extreme value within 1.5× the interquartile range; points beyond the whiskers are plotted individually. (**J**) PCA projection of Lepr/Glp1r neurons from DIO Lepr^Glp1r^KO (red) and Control (blue) mice onto the nutritional perturbation embedding. Centroids for DIO (orange) and fasted (teal) conditions shown as large circles. Lepr^Glp1r^KO neurons cluster with fasted wild-type neurons (PC1 permutation test, *P* = 0.001), indicating LepR signaling is required to adopt the DIO transcriptional state. (**K**) Volcano plot of differentially expressed genes (edgeR, Benjamini–Hochberg adjusted *P* < 0.05) in *Lepr/Glp1r* neurons (KO vs WT, DIO; *n* = 7 KO, 6 control). (**L**) PCA projection of *Agrp* neurons from DIO *Lepr*^*Glp1r*^KO (red) and Control (blue) mice; centroids for DIO (orange) and fasted (teal) conditions shown as large circles, as in (**J**). *Agrp* neurons shift toward the fasted transcriptional state in *Lepr*^*Glp1r*^KO mice (PC1 permutation test, *P* = 0.001). (**M**) Volcano plot of differentially expressed genes (edgeR, Benjamini–Hochberg adjusted *P *< 0.05) in *Agrp* neurons (KO vs WT, DIO; *n* = 7 KO, 6 control; 128 genes). Genes colored by their response to fasting: orange, fasting-upregulated; blue, fasting-downregulated; grey, neither. Source data are available online for this figure.

Previous work established that neuron populations that overlap with *Lepr/Glp1r* neurons monosynaptically inhibit *Agrp* neurons (Webster et al, 2024), but the relative contribution of this input among all *Lepr* afferents has not been quantified. Reanalysis of the published snRNA-seq data from rabies-traced *Agrp* neuron afferents revealed that *Lepr/Glp1r* neurons comprise ~80% of all *Lepr*-expressing GABAergic input to *Agrp* neurons (Fig. EV7A,B). These results identify *Lepr/Glp1r* neurons as the predominant leptin-regulated inhibitory input onto *Agrp* neurons. While prior tracing mainly examined ARC *Lepr/Glp1r* neurons (Webster et al, 2024; Tan et al, 2024); our spatial data and previous findings (Duensing et al, 2026) show these cells also reside in the DMH. To determine whether *Lepr-* and *Glp1r-*expressing neurons in both locations project onto *Agrp* neurons, we performed two complementary monosynaptic rabies tracing experiments using *Npy*^*Flp*^ on the *Lepr*^*Cre*^ reporter background and *Agrp*^*Cre*^ on the *Glp1r*^*Flp*^ reporter background (Fig. 4A,D). In both strategies, starter cells were identified in the ARC (Fig. 4B,E) and rabies-labeled presynaptic neurons were traced to DMH *Lepr* and *Glp1r* neurons from ARC *Npy* and *Agrp* neurons, respectively (Fig. 4C,F). While the co-expression of *Lepr* and *Npy* in the ARC prevents the unequivocal demonstration that rabies-labelled ARC *Lepr* neurons lie upstream of *Npy*^*Flp*^ neurons (as opposed to representing ARC *Lepr/Npy* neurons), the distribution of many traced *Lepr* cells in the dorsal lateral ARC (which contains few *Npy* neurons) suggests the presence of such afferents. *Agrp* neurons do not express *Glp1r*, however, and ARC *Glp1r* neurons were labelled with rabies retrogradely from *Agrp* neurons. Approximately 36% and 48% of rabies-traced neurons in the ARC and DMH, respectively, were *Glp1r*-positive. Hence, *Lepr-* and *Glp1r*-expressing neurons in the DMH, as well as in the ARC, provide direct input onto *Agrp*/*Npy* neurons.

### Leptin action on *Lepr/Glp1r* neurons drives *Agrp* neuron transcriptional responses to obesity

Having established that *Lepr/Glp1r* neurons provide the predominant input to *Agrp* neurons from GABAergic *Lepr* neurons, we asked whether cell-autonomous leptin signaling drives their adaptation to obesity and, consequently, mediates the response of *Agrp* neurons to DIO. We profiled neurons from mice lacking *Lepr* specifically in *Glp1r*-expressing neurons (Lepr^Glp1r^KO mice) on chow and after 15 weeks of HFD, when leptin is chronically elevated and the consequences of removing leptin signaling should be most apparent (35,538 cells; Figs. 4G–I and EV7C–E).

As expected, LGS expression was significantly reduced in *Lepr/Glp1r* neurons from Lepr^Glp1r^KO mice, with a significant genotype × diet interaction – indicating that the effect was most pronounced under DIO conditions (Figs. 4J and EV7F). When projected onto the principal component embedding derived from the nutritional perturbation dataset (Fig. 2B), *Lepr/Glp1r* neurons from Lepr^Glp1r^KO mice clustered with those from fasted wild-type mice regardless of diet (Fig. 4J; PC1 shift permutation test, *P* = 0.001), indicating that cell-autonomous LepR signaling is required for these neurons to adopt the DIO-associated transcriptional state. Loss of *Lepr* abolished the DIO-associated induction of immediate early genes (*Fos, Vgf, Homer1*) and reversed the downregulation of GABA receptor subunits in *Lepr/Glp1r* neurons, suggesting that loss of LepR signaling decreases the activity of and increases inhibitory input to these cells (Fig. 4K; Tables EV8 and 9).

Loss of LepR signaling in *Lepr/Glp1r* neurons also altered gene expression in *Agrp* neurons in obese animals. While *Agrp* neurons from chow-fed Lepr^Glp1r^KO mice were transcriptionally indistinguishable from controls, 128 genes were differentially expressed in *Agrp* neurons from DIO Lepr^Glp1r^KO mice compared to DIO controls (Fig. 4L,M; Tables EV10 and 11). Notably, fasting-responsive genes were strongly enriched among the differentially expressed genes in *Agrp* neurons: those upregulated by fasting were overrepresented among genes increased in Lepr^Glp1r^KO mouse *Agrp* neurons, and those downregulated by fasting were overrepresented among genes with decreased expression in the DIO knockout animals (Fig. 4M). Projection of Lepr^Glp1r^KO *Agrp* neurons onto the diet PCA embedding confirmed a significant shift toward the fasting transcriptional state (PC1 shift permutation test, *P* = 0.001; Fig. 4L). In other words, *Agrp* neurons in Lepr^Glp1r^KO mice maintain a fasting-like activated transcriptional state despite obesity. Thus, direct leptin action on *Lepr/Glp1r* neurons is required to drive the response of *Agrp* neurons to DIO.

### Activation of *Lepr/Glp1r* neurons suppresses *Agrp* neuron-dependent feeding

**Figure EV8:**
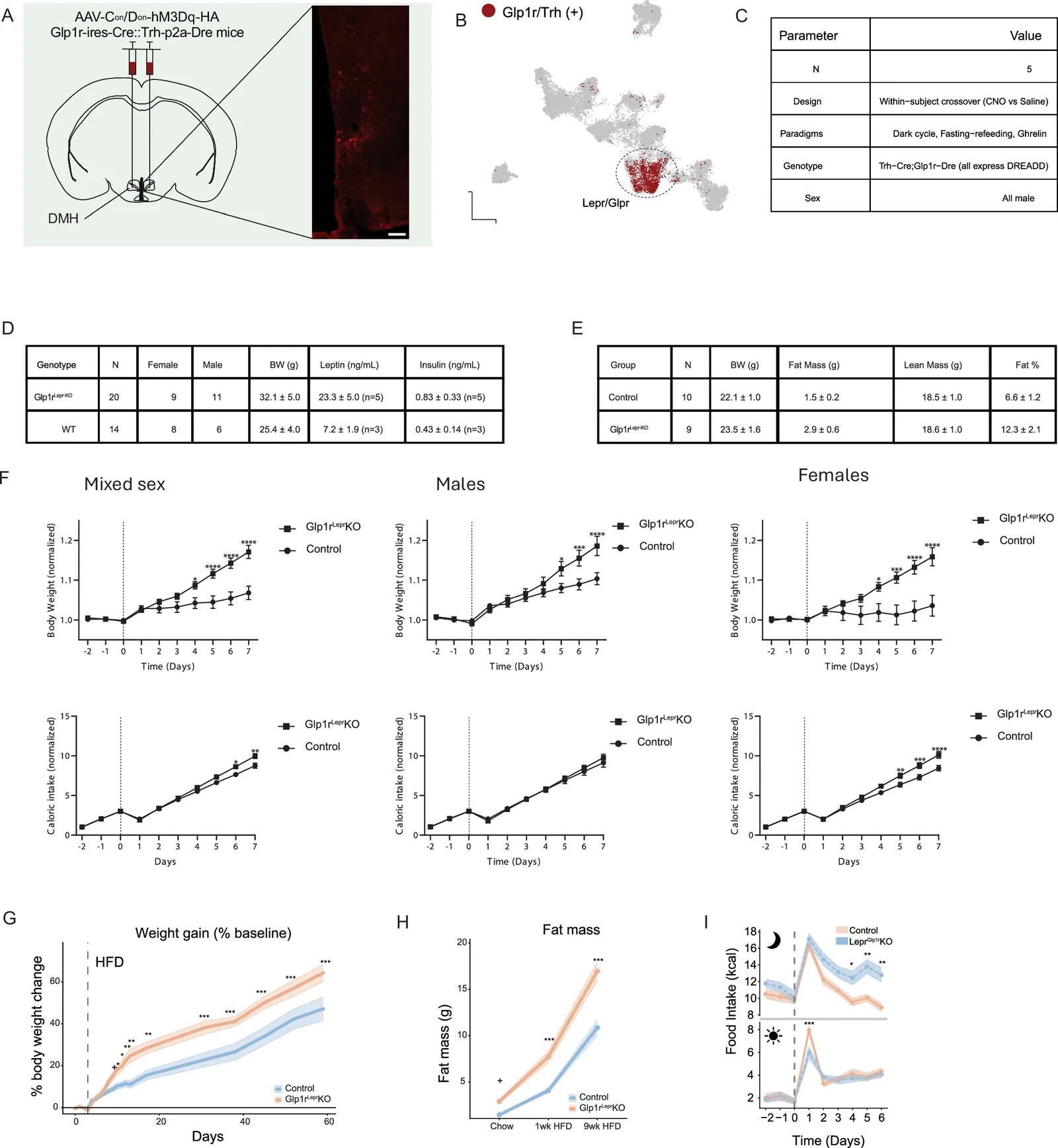
DREADD validation, baseline characterization, and HFD-induced weight gain and food intake in Glp1rLepr-KO mice. (**A**) Schematic and representative HA-IR (red) image from bilateral AAV-FLEX-FREX-hM3Dq-HA injection into the caudal ARC/ventral DMH of *Glp1r*-ires-Cre;*Trh*-p2a-Dre mice. Scale bar: 200 µm. (**B**) Spatial transcriptomics reference map with projected *Trh/Glp1r* neurons (red); *Lepr/Glp1r* neuron population is curled in dashed line. (**C**) Summary of the DREADD cohort (main text Fig. 5A–C). (**D**) Baseline characteristics of chow-fed Lepr^Glp1r^KO (*n* = 20) and control (*n* = 14) mice used for leptin-dependent feeding suppression experiments (main text Fig. 5D,E). (**E**) Baseline chow-period body weight and DEXA body composition of Lepr^Glp1r^KO (*n* = 9) and control (*n* = 10) mice prior to HFD exposure. Data are mean ± SD. (**F**) Cumulative food intake following HFD switch. Cumulative energy intake (kcal) in control (circle) and Lepr^Glp1r^KO (square) mice before and after introduction of HFD (dashed line) separated by sex. (**G**) Percent body weight change from baseline in control and Lepr^Glp1r^KO mice over 60 days of HFD feeding. (**H**) Fat mass measured by ECHO-MRI at baseline (chow), 1 week, and 9 weeks of HFD feeding. (**I**) Daily food intake (kcal) in control and Lepr^Glp1r^KO mice in the days immediately before and after introduction of HFD. Data are mean ± SEM. Asterisks denote BH-adjusted *P* values from within-timepoint contrasts: ^+^*P* < 0.1, *P < 0.05, **P < 0.01, ***P < 0.001.

**Figure 5:**
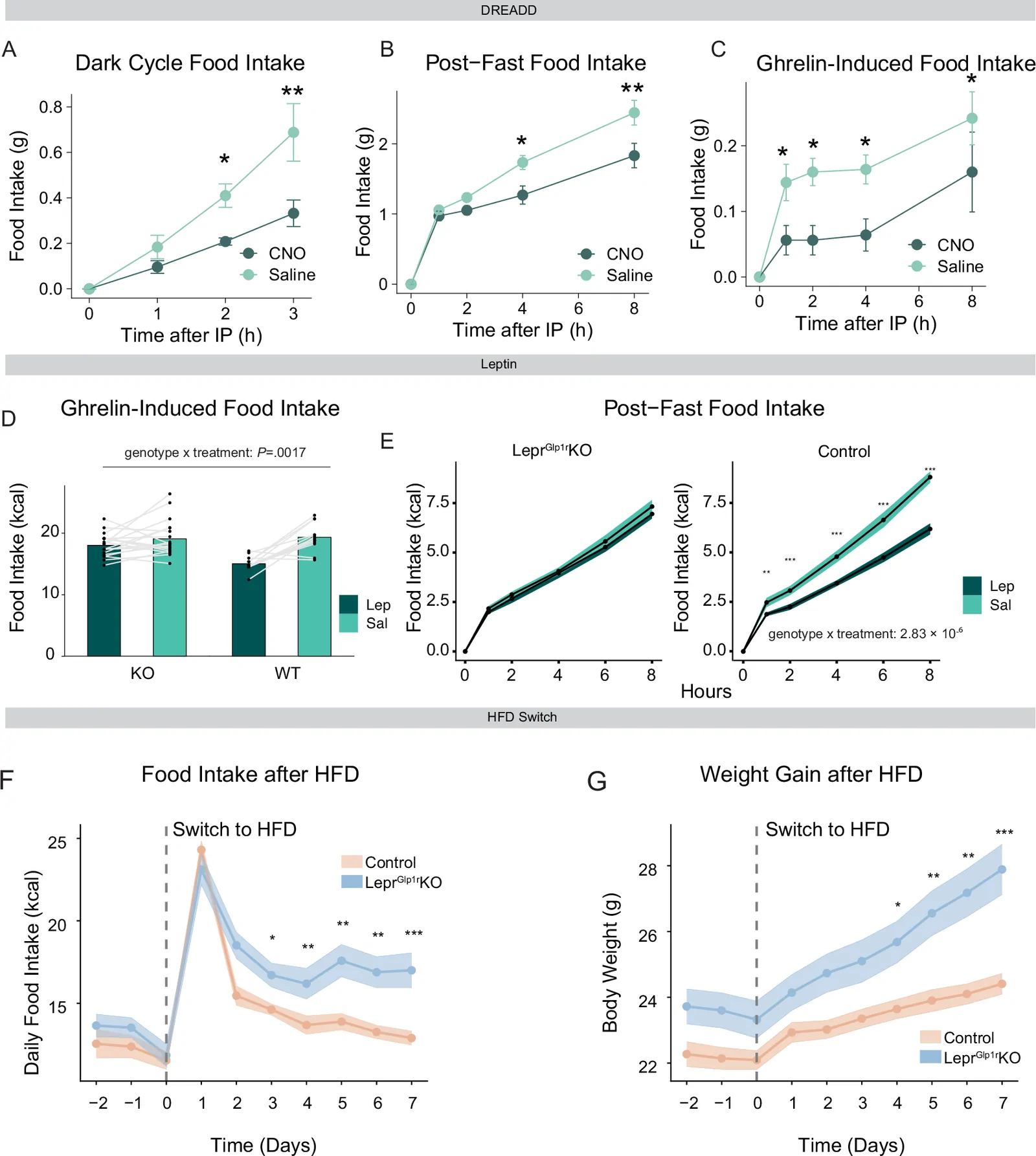
*Lepr/Glp1r* neurons restrain hyperphagic responses. (**A**–**C**) Mice containing activating DREADDs in *Glp1r/Trh* neurons were treated with saline (light teal) or CNO (dark teal) at the onset of the dark cycle, during refeeding following an overnight fast, or prior to ghrelin treatment. (**A**) Cumulative dark-cycle food intake at 0, 1, 2, and 3 h following IP CNO or saline in a within-subject crossover design (*P* = 8.2 × 10^–8^, *n* = 5). (**B**) Cumulative post-fast food intake at 0, 1, 2, 4, 6, and 8 h following IP CNO or saline (*P* = 5.8 × 10^–5^, *n* = 5). (**C**) Cumulative food intake following IP ghrelin with CNO or saline pre-treatment at 0, 1, 2, 4, 6, and 8 h (*P* = 1.4 × 10⁻⁸, *n* = 4). (**D**) Effect of leptin (dark teal) versus saline (light teal) pre-treatment on ghrelin-induced 24-h food intake in mixed sex lean *Lepr*^*Glp1r*^KO (KO) and control (WT) mice. Lines connect within-subject measurements (crossover design). Linear mixed-effects model, leptin × genotype: *P* = 1.7 × 10^–3^. (**E**) Cumulative post-fast food intake following leptin (dark teal) or saline (light teal) administration in mixed sex WT (left) and KO (right) mice (treatment × genotype: *P* = 2.8 × 10^–6^). (**F**, **G**) Daily food intake (**F**; genotype × time: *P *= 5.7 × 10^–9^) and body weight (**G**; genotype × time: *P* = 2.5 × 10^–28^) before and 7 days after HFD exposure in mixed sex Lepr^Glp1r^KO (blue; *n* = 9) and Control (peach; *n* = 10) mice. Dashed line indicates HFD switch. All panels: *P < 0.05, **P < 0.01, ***P < 0.001. (**A**–**C**, **E**–**G**) Plotted points represent mean values. Error bars (**A**–**C**) and shaded regions (**E**–**G**) denote SEM. Source data are available online for this figure.

If leptin acts through *Lepr/Glp1r* neurons to inhibit *Agrp* neurons, then direct activation of these neurons should suppress feeding responses that rely on *Agrp* neuron activity. To activate *Lepr/Glp1r* neurons selectively, we used an intersectional genetic approach requiring two recombinases (Cre and Dre) to restrict DREADD expression to neurons co-expressing *Glp1r* (via Cre) and *Trh* (via Dre)—markers whose combination identifies the *Lepr/Glp1r* population in the ARC and ventral DMH (Fig. EV8A,B). Using a within-subject crossover design, we found that chemogenetic activation of *Lepr/Glp1r* neurons suppressed dark-cycle food intake, reduced refeeding after an overnight fast, and blunted ghrelin-induced hyperphagia (Figs. 5A–C and EV8C). Thus, activation of *Lepr/Glp1r* neurons is sufficient to suppress these feeding processes.

We next used Lepr^Glp1r^KO mice (Fig. EV8D,E) to ask whether LepR signaling in *Lepr/Glp1r* neurons is required for exogenous leptin to suppress these feeding behaviors. Exogenous leptin failed to blunt ghrelin-induced feeding in Lepr^Glp1r^KO animals (Fig. 5D). Similarly, leptin suppressed refeeding following an overnight fast in wild-type mice but failed to reduce intake in Lepr^Glp1r^KO mice (Fig. 5E). Together, these findings identify *Lepr/Glp1r* neurons as the effector population through which exogenous leptin suppresses hyperphagic responses in which *Agrp* neurons participate.

### Leptin signaling in *Lepr/Glp1r* neurons constrains hyperphagia during the onset of DIO

Having established that exogenous leptin requires *Lepr/Glp1r* neurons to suppress the hyperphagia that results from fasting or ghrelin treatment, we asked whether this mechanism operates during the physiological rise in leptin that accompanies weight gain. A robust but transient hyperphagia accompanies the switch to HFD in WT mice. This hyperphagia typically peaks after 1–2 days and then normalizes over the following week as body weight is gained and leptin levels rise (Xu et al, 2012). Since *Lepr/Glp1r* neurons restrain food intake and represent the major cell population that responds to hyperleptinemia and DIO, then the progressive attenuation of HFD-driven hyperphagia should require intact leptin signaling in these neurons.

Immediately following the switch to HFD, both Lepr^Glp1r^KO and control mice exhibited a comparable hyperphagia (Figs. 5F and EV8F). While food intake in control animals rapidly declined and returned to baseline by day 7, feeding remained elevated in Lepr^Glp1r^KO mice (Fig. 5F). Consequently, Lepr^Glp1r^KO mice gained significantly more weight than controls (Fig. 5G). Following feeding and body weight for these animals over the subsequent 8 weeks revealed that the weight gain trajectory of Lepr^Glp1r^KO mice eventually slowed, thereafter paralleling that of control animals (Fig. EV8G–I). These data argue that the genetic lesion, rather than increased adiposity, drives the initial failure of Lepr^Glp1r^KO mice to rapidly normalize food intake and also suggest that non-*Lepr/Glp1r*-dependent mechanisms eventually normalize food intake, albeit only after the attainment of a much higher body weight/adiposity.

### *Agrp* neuron desensitization in obesity requires leptin signaling in *Lepr/Glp1r* neurons

**Figure EV9:**
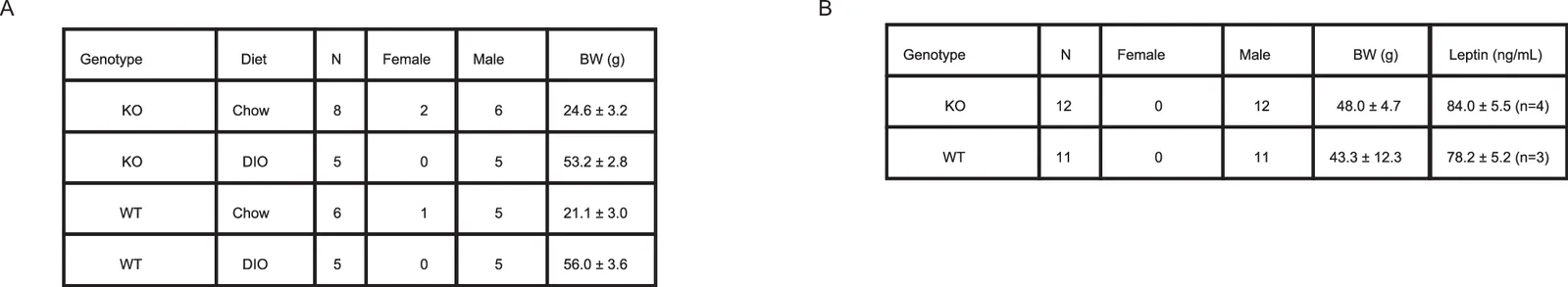
Baseline characteristics and plasma leptin levels of Glp1rLepr-KO and control mice used in fasting-refeeding experiments (**A**) Baseline characteristics of Lepr^Glp1r^KO and control mice used in the chow vs DIO fasting-refeeding experiment (main text Fig. 6A). Chow-fed and DIO cohorts are independent groups of animals. (**B**) Summary table including plasma leptin for a subset of mice (KO *n* = 4, WT *n *= 3). Data are mean ± SD (tables) or mean ± SEM (dot plots) with individual animals shown.

**Figure 6:**
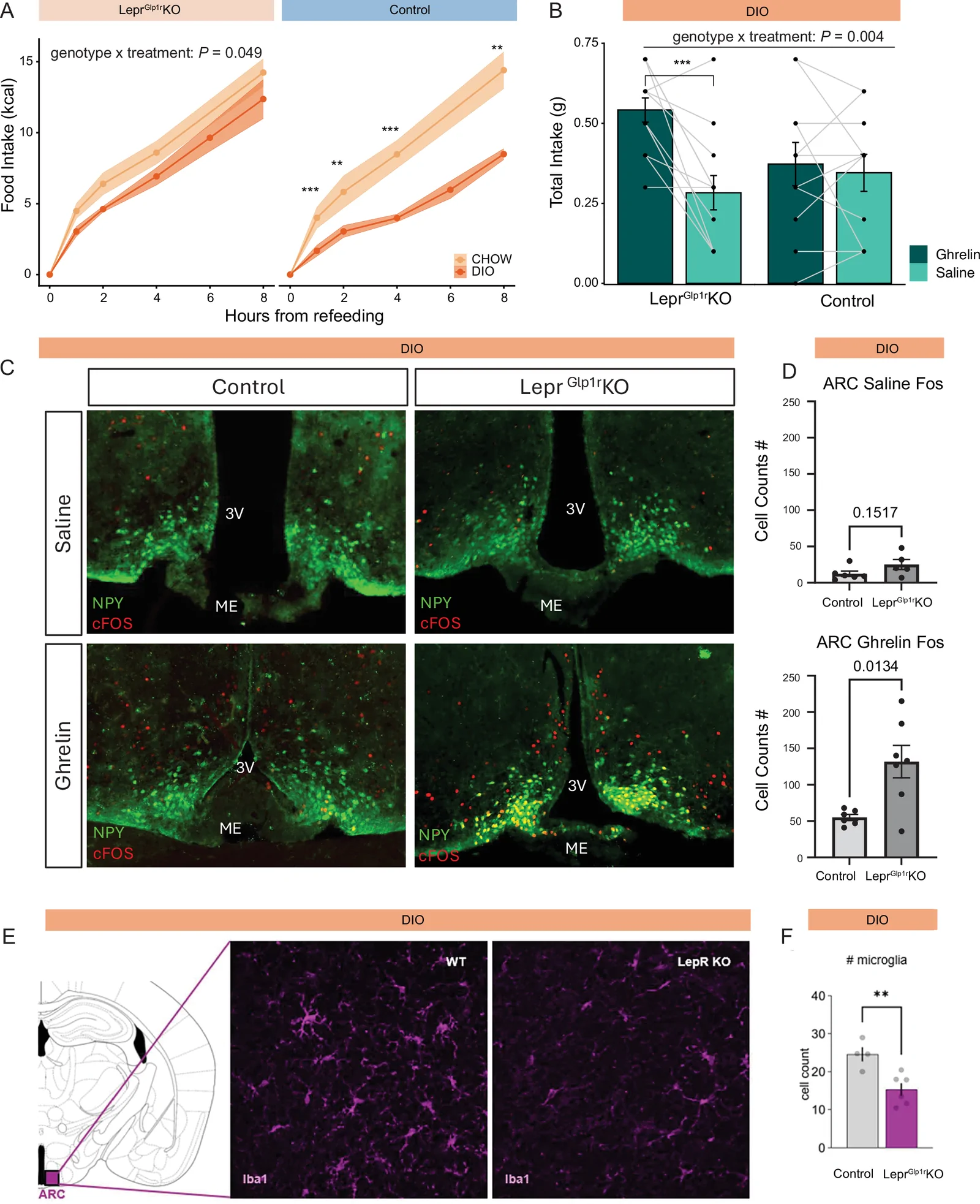
Leptin signaling in *Lepr/Glp1r* neurons mediates restraint of hyperphagia and *Agrp* neurons, and promotes hypothalamic microgliosis during DIO. (**A**) Cumulative post-fast food intake (kcal) at 0, 1, 2, 4, and 8 h in mixed sex control (WT, left) and *Lepr*^*Glp1r*^KO (right) mice on chow (light) or HFD (dark). Linear mixed-effects model, genotype × diet interaction: *P* = 0.049; within-genotype: WT: *P* = 5.5 × 10^–5^, KO: *P* = 0.074. *P < 0.05, **P < 0.01, ***P < 0.001. Shaded regions denote SEM. (**B**) Five-hour food intake following IP ghrelin (dark) or saline (light) in mixed sex DIO WT (*n* = 11) and *Lepr*^*Glp1r*^KO (*n* = 12) mice. Linear mixed-effects model, genotype × treatment *P* = 4.1 × 10^–3^; ghrelin vs saline: KO *P* = 1.7 × 10^–4^, WT *P* = 0.70. Lines connect within-subject measurements (crossover design). **P < 0.01, error bars denote SEM. (**C**) Representative FOS-IR (red) and GFP-IR (green) in the mbARC of DIO Control and *Lepr*^*Glp1r*^KO mice on the Npy-Gfp background following saline (top) or ghrelin (bottom) injection. 3V third ventricle, ME median eminence. (**D**) Quantification of FOS-positive mbARC neurons after saline (top; *P* = 0.152, *n* = 6 control, 5 KO) or ghrelin (bottom; *P* = 0.013, *n* = 6 control, 7 KO). Shown are mean−/+ SEM, along with individual data points. (**E**) Left: schematic indicating the mbARC region sampled. Right: representative IBA1 immunofluorescence in DIO WT and *Lepr*^*Glp1r*^KO (KO) mice. (**F**) Quantification of IBA1-positive microglia in the ARC of Control and *Lepr*^*Glp1r*^KO mice. Shown are mean−/+ SEM, along with individual data points. **P < 0.01 by unpaired *t* test. Source data are available online for this figure.

The reduced sensitivity of *Agrp* neurons to orexigenic stimuli, including fasting and ghrelin (Beutler et al, 2020), represents a hallmark of DIO. Because our data indicated that *Lepr/Glp1r* neurons mediate most of the transcriptional response of *Agrp* neurons to DIO, we asked whether loss of leptin signaling in this population would disinhibit *Agrp* neurons and restore hyperphagic responses in obese animals. We measured feeding responses to two canonical *Agrp* neuron-activating stimuli, fasting and ghrelin, in chow-fed and DIO Lepr^Glp1r^KO mice (Fig. EV9). Consistent with prior reports that obesity suppresses *Agrp* neuron responsiveness (Beutler et al, 2020; Briggs et al, 2010), refeeding after an overnight fast was blunted in DIO wild-type animals, which consumed roughly half the calories of lean controls (Fig. 6A). In contrast, DIO Lepr^Glp1r^KO mice consumed similar amounts of food as lean controls, indicating that the DIO-associated suppression of post fast refeeding was largely abolished. Lepr^Glp1r^KO mice are more obese than control animals; hence, increased leptin action on Lepr/Glp1r neurons (rather than some other effect of increased adiposity) must mediate the restraint of past-fast hyperphagia in DIO.

Similarly, ghrelin failed to stimulate feeding in DIO wild-type animals but significantly increased intake in DIO Lepr^Glp1r^KO mice (Fig. 6B), consistent with restored *Agrp* neuron ghrelin sensitivity in these animals. Indeed, although baseline FOS-IR in the mediobasal ARC (mbARC) region (which mainly represents FOS in *Npy* neurons) did not differ between genotypes, ghrelin elicited increased mbARC FOS-IR in DIO Lepr^Glp1r^KO mice compared to DIO controls (Fig. 6C). Hence, loss of LepR in *Lepr/Glp1r* neurons restores ghrelin-responsiveness to *Agrp* neurons in DIO animals. Together, these findings suggest that the normally blunted activation of *Agrp* neurons and hyperphagic responses in obesity reflect increased output from leptin-responsive *Lepr/Glp1r* neurons.

### Loss of leptin signaling in *Lepr/Glp1r* neurons reduces hypothalamic neuroinflammation in obesity

Unlike DIO mice, obese leptin-deficient animals do not exhibit the hypothalamic microgliosis that usually accompanies, suggesting that this hallmark of obesity requires intact leptin signaling (Briggs et al, 2014). Because the phenotype of Lepr^Glp1r^KO mice suggests that these neurons mediate many responses to DIO, we asked whether they might also contribute to this obesity-associated hypothalamic neuroinflammation. Indeed, immunohistochemical staining for the microglial marker, IBA1 (Fig. 6D), revealed that, despite their greater food consumption and body weight, the ARC of DIO Lepr^Glp1r^KO mice contains fewer microglia than DIO controls (*P* < 0.01). Hence, obesity-associated MBH neuroinflammation is driven at least in part by leptin action on *Lepr/Glp1r* neurons.

Notably, each of the DIO-associated phenotypes altered by loss of leptin signaling in *Lepr/Glp1r* neurons—blunted ghrelin-stimulated *Agrp* neuron activation and hyperphagia, attenuated fasting-induced refeeding, and hypothalamic microglial activation—is also absent in leptin-deficient *Lep*^*ob/ob*^ mice despite comparable or greater obesity (Briggs et al, 2014; Gao et al, 2014). Thus, these hallmarks of DIO are not consequences of obesity per se, but rather result from sustained leptin action through *Lepr/Glp1r* neurons.

## Discussion

The concept of ‘leptin resistance’ rests on two observations: food intake remains normal or elevated despite hyperleptinemia in obesity and exogenous leptin fails to reduce feeding and weight in DIO. Our findings reframe this phenomenon: while LepR signaling in most *Lepr* neuron types negligibly increases in DIO, rising leptin levels in obesity progressively engage a specific neuronal population—*Lepr/Glp1r* neurons—to limit hyperphagia and additional weight gain.

Our data also suggest that the pathogenesis of DIO involves multiple, distinct phases. Exposure to a palatable diet initially triggers a robust hyperphagia while circulating leptin remains low prior to the onset of substantial weight gain. This response begins to change as body weight, fat mass, and leptin levels increase, however. While most *Lepr* neurons do not respond to these second phase changes in DIO, rising leptin levels continue to augment LepR signaling in the uniquely leptin-responsive *Lepr/Glp1r* neurons. This leads to the near normalization of food intake over the 7–10 days following the initiation of HFD feeding. This sustained leptin action on *Lepr/Glp1r* neurons helps to establish a new, biologically defended body weight that, while higher than before, remains well below what would occur in the absence of the response of *Lepr/Glp1r* neurons to leptin. While the increased inhibition of *Agrp* neurons by *Lepr/Glp1r* neurons presumably contributes to these responses, *Lepr/Glp1r* neurons may also restrain other orexigenic circuits.

The reduced overall responsiveness to exogenous leptin in DIO likely reflects multiple processes, including the failure of hyperleptinemia to increase LepR signaling in most *Lepr* cell types. Furthermore, there likely exists an ultimate ceiling effect for the ability of *Lepr/Glp1r* neurons to suppress feeding; not only might *Lepr/Glp1r* neurons eventually reach maximal LepR signaling in DIO, but also *Agrp* neurons (and/or other downstream targets) may be fully inhibited (neuronal activity cannot go below zero).

**Figure EV10:**
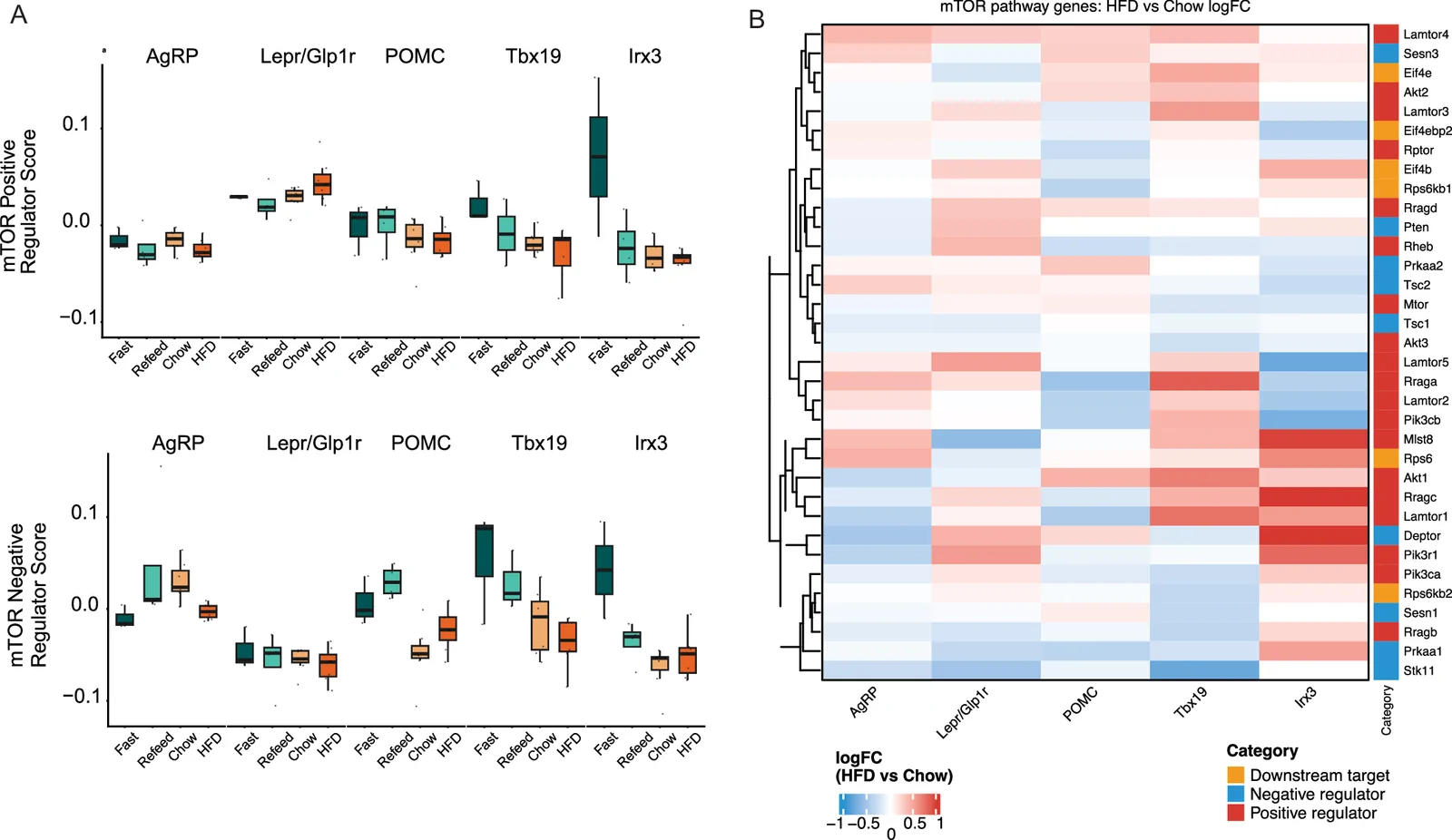
mTOR pathway gene expression across Lepr neuron populations does not explain diff erential leptin sensitivity in DIO. (**A**) Expression levels of selected mTOR pathway positive regulators (top) and negative regulators (bottom) across hypothalamic *Lepr* neuron populations from snRNA-seq data. *Lepr/Glp1r* neurons tend to express higher levels of mTOR-activating genes and lower levels of mTOR-inhibiting genes relative to other *Lepr* populations. Box plots show median, interquartile range, and whiskers extending to 1.5×IQR. (**B**) Heatmap of log_2_ fold-change (HFD vs Chow) for mTOR pathway genes across *Lepr* neuron populations. Genes are annotated by functional category: positive regulators (red), negative regulators (blue), and downstream targets (yellow) of mTOR signaling. Despite elevated baseline mTOR pathway gene expression, *Lepr/Glp1r* neurons retain robust leptin sensitivity in DIO, arguing against a simple model in which mTOR hyperactivation uniformly drives leptin resistance across all *Lepr* populations.

Notably, canonical feedback inhibitors do not explain why *Lepr/Glp1r* neurons escape the general loss of leptin sensitivity. SOCS3, a well-characterized terminator of LepR signaling (Bjørbæk et al, 1998), is strongly induced in *Lepr/Glp1r* neurons during DIO yet fails to block their transcriptional response. Conversely, *Agrp* neurons show minimal *Socs3* induction yet respond poorly to elevated leptin. Furthermore, our transcriptomic data do not reveal evidence of previously proposed mechanisms such as inflammatory or ER-stress signaling (Zhang et al, 2008; Thaler et al, 2012) in *Lepr* neurons during DIO. Similarly, although mTOR hyperactivation has been proposed to impair leptin signaling in DIO (Tan et al, 2025), *Lepr/Glp1r* neurons tend to express higher levels of mTOR activators and lower levels of mTOR inhibitors than other *Lepr* populations, consistent with increased mTOR activity in these very leptin-responsive cells (Fig. EV10). Rather, it is possible that *Lepr/Glp1r* neurons operate across a wider dynamic range of leptin concentrations because they express more *Lepr* than other cell types, permitting their continued responsiveness to increased leptin concentrations. The resultant sustained output of *Lepr/Glp1r* neurons onto downstream targets (including *Agrp* neurons) thus drives the diminished responsiveness of these circuits to stimuli such as fasting and ghrelin.

Although *Agrp* and *Npy* expression was normal in Lepr^Glp1r^KO mice, our data show that indirect leptin action via *Lepr/Glp1r* neurons mediate the most prominent effects on Agrp neuron gene expression. Furthermore, animals lacking LepR on *Agrp* neurons exhibit mildly increased body weight, this phenotype is more modest than that of Lepr^Glp1r^KO mice (van de Wall et al, 2008). Although we cannot rule out the possibility that developmental effects impact the strength of these phenotypes, these findings are consistent with the emerging view that leptin’s effect on *Agrp* neurons is predominantly indirect, mediated through upstream GABAergic *Lepr/Glp1r* neurons that constitute the majority of inhibitory *Lepr* neuron input to this population (Garfield et al, 2016; Webster et al, 2024; Aitken et al, 2024). In DIO, this indirect pathway becomes the principal means by which additional leptin modulates *Agrp* neurons.

*Agrp* neurons respond to a host of stimuli other than leptin, including food presentation, nutrition-related signals from the gut, and circadian cues, among others (Chen et al, 2015; Garfield et al, 2016; Sayar-Atasoy et al, 2023; Douglass et al, 2024). Distributing leptin signaling to an upstream inhibitory node in addition to direct leptin signaling on *Agrp* neurons enables the energy state-dependent modulation of *Agrp* neurons through multiple mechanisms. Leptin action on *Lepr/Glp1r* neurons modulates inhibitory tone proportional to energy stores, while direct leptin action on *Agrp* neurons presumably modulates the intrinsic excitability of the neuron. Because increased leptin in DIO increases *Lepr/Glp1r*-dependent inhibitory tone on *Agrp* neurons without changing the direct leptin-regulated intrinsic parameters of *Agrp* neurons, *Agrp* neurons presumably remain somewhat responsive to activating stimuli in obesity.

Two additional consequences of sustained *Lepr/Glp1r* neuron activation merit comment. First, the well-documented insensitivity of *Agrp* neurons to ghrelin in DIO (Beutler et al, 2020; Briggs et al, 2010) is reversed by removing leptin signaling from *Lepr/Glp1r* neurons, suggesting that ghrelin resistance in obesity is not cell-intrinsic to *Agrp* neurons but reflects extrinsic inhibition by this upstream population. Second, the attenuation of hypothalamic microgliosis in Lepr^Glp1r^KO mice—mirroring the phenotype of leptin-deficient mice (Gao et al, 2014)—indicates that obesity-associated neuroinflammation is driven by leptin signaling in *Lepr/Glp1r* neurons, rather than by adiposity per se.

In sum, the selective preservation of leptin signaling in *Lepr/Glp1r* neurons reframes what has been termed leptin resistance as the combination of a ceiling effect for leptin action on most neuron types plus increased leptin action via a single crucial circuit. Understanding the mechanisms that prevent increased LepR signaling in other (non-*Lepr/Glp1r*) neurons in DIO and determining mechanisms by which to sensitize these neurons to elevated leptin in DIO might improve the therapy of obesity. Indeed, it is possible that the ability of some gut peptide-based obesity therapies to increase leptin sensitivity could flow from the restoration of leptin sensitivity to *Lepr* neurons that generally fail to respond to elevated leptin in obesity. Conversely, the selectivity of increased leptin action for *Lepr/Glp1r* neurons suggests the potential importance of not augmenting the leptin responsiveness of other *Lepr* neurons in DIO- perhaps because increased leptin action on these cells could promote adverse effects (e.g., hypertension due to increased sympathetic tone). Future work should also explore how increased leptin action on *Lepr/Glp1r* neurons in DIO might reshape the response to other feeding-relevant signals, including cold exposure (Deem et al, 2020) and gut-derived satiety signals (and their peptide mimetics) (Beutler et al, 2020). Resolving these questions will be essential for determining whether the circuit-specific leptin action described here can be harnessed therapeutically.

## Methods

**Table:** Reagents and tools table

Reagent/resource	Reference or source	Identifier or catalog number
**Experimental models: organisms/strains (mouse)**		
C57BL/6 N/J (wild-type)	The Jackson Laboratory	Cat# 005304
Glp1r-ires-Cre	The Jackson Laboratory	Cat# 029283
Trh-p2a-Dre	Grzelka et al, (2023) (see manuscript)	N/A
Lepr^flox (Lepr conditional allele)	Myers lab, University of Michigan	N/A
Lepr^Glp1rKO (Lepr^flox × Glp1r-ires-Cre)	This study	N/A
Glp1r^Flp (p2A-FlpO at endogenous Glp1r 3′)	This study	N/A
Npy^Flp	The Jackson Laboratory	Cat# 030211
Agrp^Cre	The Jackson Laboratory	Cat# 012899
Rosa26^FSF-tdTomato	The Jackson Laboratory	Cat# 032864
Rosa26^LSL-Sun1-sfGFP	The Jackson Laboratory	Cat# 021039
**Antibodies**		
Rabbit anti-pSTAT3 (1:200)	Cell Signaling Technology	Cat# 9145; RRID:AB_2491009
Goat anti-rabbit IgG AF594 (1:200) — pSTAT3 secondary	Invitrogen	Cat**#** A-11012
Rabbit anti-HA epitope tag (1:250)	Rockland Immunochemicals	Cat# 600-401-384
Anti-rabbit IgG Alexa Fluor 555 (1:1000) — HA secondary	Life Technologies/Thermo Fisher	Cat # A-21428
Rabbit anti-FOS (1:1000)	Cell Signaling Technology	Cat# 2250S; RRID:AB_2247211
Rabbit anti-IBA1 (1:1000)	FUJIFILM Wako	Cat# 019-19741; RRID:AB_839504
Donkey anti-rabbit IgG Alexa Fluor 568 (1:250)	Invitrogen/Thermo Fisher	Cat# A-10042
Donkey anti-rabbit IgG Alexa Fluor 647 (1:250)	Invitrogen/Thermo Fisher	Cat# A-31573; RRID:AB_2536183
TotalSeq-A anti-Nuclear Pore Complex Proteins hashtag antibodies 1–15 (A0451-A0465)	BioLegend	Cat# 682205–682219; RRID:AB_2861054 – AB_2861068
**Chemicals, peptides, and recombinant proteins**		
Mouse recombinant leptin (snRNA-seq IP)	R&D Systems	Cat# 498-OB
Recombinant Mouse Leptin Protein, CF	R&D Systems	Catalog # 498-OB
Ghrelin	Hello Bio	Cat# HB2942
Clozapine N-oxide (CNO)	Hello Bio	Cat# HB6149
Tris-HCl, 20mM, pH 8.0	Invitrogen/Thermo Fisher	Cat# 10434742
Nuclei EZ lysis buffer	Sigma-Aldrich	Cat# NUC101
OptiPrep (iodixanol)	Sigma-Aldrich	Cat# D1556
Protector RNase inhibitor	Roche	Cat# 3335399001
VECTASHIELD antifade mounting medium	Vector Laboratories	Cat# H-1000
VECTASHIELD antifade mounting medium with DAPI	Vector Laboratories	Cat# H-1200
Fluoromount-G mounting medium	SouthernBiotech	Cat# 0100-01
KAPA HiFi HotStart ReadyMix (2X)	Roche Diagnostics	Cat# 07958935001
SPRIselect beads	Beckman Coulter	Cat# B23317
EB buffer	Qiagen	Cat# 1014609
**Critical commercial assays/kits**		
Chromium Next GEM Single Cell 3′ kit, dual index, v3 / v3.1	10x Genomics	N/A
Qubit dsDNA assay	Invitrogen/Thermo Fisher	Cat# Q32851
TapeStation High Sensitivity D1000 DNA kit	Agilent	Cat# 5067-5584
Xenium In Situ Gene Expression (slides, probes, reagents)	10x Genomics	
**Recombinant DNA/viral vectors**		
AAV1/2-FLEX-FREX-hM3Dq	Viral Vector Facility, ETH Zurich	Cat# V892-1
Deposited data		
Raw single-nucleus RNA-seq (this study)	EMBL-EBI BioStudies/ ArrayExpress	E-MTAB-XXX
Processed single-cell & spatial data (this study)	Zenodo	
Analysis code (this study)	GitHub	github.com/perslab/Rausch-2025
TRAP-seq, leptin-treated hypothalamic Lepr neurons	Allison et al (see manuscript)	GEO: GSE162603
RNA-seq, hypothalamic Lepr neuron nuclei after leptin	Public dataset (see manuscript)	GEO: GSE112125
**Software and algorithms**		
R	R Core Team	v4.3.3; https://www.r-project.org
Xenium Analyzer onboard analysis software	10x Genomics	v2.0.1.0
QuPath	Bankhead et al, 2017	v0.5.0
bcl2fastq	Illumina	v2.19.01
Cell Ranger	10x Genomics	v7.0
CellBender	Fleming et al, 2023	N/A
kb-python/kallisto|bustools	Melsted et al, 2021	v0.24.4
Seurat	Hao et al, 2021	v4.3.0
scDblFinder	Bioconductor	N/A
scuttle	Bioconductor	v1.4
cellAnnotator	Kharchenko Lab	N/A
mclust (Gaussian mixture modelling)	CRAN	N/A
DESeq2	Bioconductor	v1.44.0
mixOmics (MINT-sPLS-DA)	Bioconductor	N/A
edgeR	Bioconductor	v4.0.16
limma / limma-voom	Bioconductor	v3.58.1
scDist	Nicol et al, (2024) (see manuscript)	v1.1.0
**Other (equipment/instruments)**		
Xenium Analyzer instrument	10x Genomics	N/A
Axioscan Z1 slide scanner (20x)	Zeiss	N/A
Slide scanner (40x)	Olympus	N/A
BX53 upright microscope (10x)	Olympus	N/A
Coronal mouse brain slicer matrix	Zivic Instruments/ASI Instruments	Cat# BSMAS005-1/RBM-2000C
KIMBLE 2 mL tissue Dounce homogenizer (pestle B)	Sigma-Aldrich (Kimble)	Cat# D8938
40 µm mini cell strainers	pluriSelect	Cat# 43-10040
SH800 cell sorter	Sony Biotechnology	N/A
SH800 70 µm sorting chip	Sony Biotechnology	Cat# LE-C3207
NovaSeq 6000 sequencer (SP & S2 flow cells)	Illumina	N/A
EchoMRI body composition analyzer	Echo Medical Systems	N/A
Stereotaxic frame	David Kopf Instruments	N/A
**Diets**		
Chow — Altromin 1310 (wild-type)	Altromin	1310
Chow — Purina 5LOD (Lepr^Glp1rKO^ & controls)	LabDiet/Purina	5LOD
Chow — ssniff V1154 (DREADD cohort)	ssniff Spezialdiäten	V1154
HFD — 58 kcal% fat & sucrose (wild-type DIO)	Research Diets	D12331i
HFD — 60 kcal% fat (Lepr^Glp1rKO DIO & controls)	Research Diets	D12492

### Mouse experiments

#### Animals and diets

C57BL/6N/J mice (Jackson Laboratory, #005304) were used as the wild-type group in the experiments. A *Glp1r*-specific knockout of *Lepr* (Lepr^Glp1r^KO mice) was produced by crossing the Lepr^flox^ mice with *Glp1r-ires-Cre* mice (Jackson Laboratory, #029283). For the DREADD experiments, *Glp1r-ires-Cre* (Williams et al, 2016); Jackson Laboratory, #029283) and *Trh-p2a-Dre* mice^35^ were utilized. *Npy*^*Flp*^, *Agrp*^*Cre*^, and the Flp- and Cre dependent reporter backgrounds (*Rosa26*^*FSF-tdTomato*^ and *Rosa26*^*LSL-Sun1-sfGfp*^ mice) were obtained from Jackson labs (Cat#: 030211, 012899, 032864, and 020139, respectively) and bred in our colony. *Glp1r*^*Flp*^ mice (a fusion of p2A-FlpO to the 3’ end of the endogenous *Glp1r* coding sequence) will be described elsewhere.

Throughout the studies, mice had *ad libitum* access to food (unless otherwise indicated) and water and were housed under specific pathogen-free conditions in temperature-controlled rooms with 75–80% humidity. Mice were maintained at a 12:12 h light cycle. All mice were fed the standard chow diets unless otherwise stated: the wild-type mice were fed Altromin 1310, Lepr^Glp1r^KO mice and their controls were fed Purina Diets 5LOD, and animals used for the DREADD experiment were fed ssniff®, V1154. The wild-type DIO mice were fed HFD with 58 kcal% fat and sucrose (Research Diets, D12331i), while DIO Lepr^Glp1r^KO mice and their controls were fed a 60 kcal% HFD (Research Diets, D12492). Only male mice were used for behavioral DREADD experiments. Experiments involving wild-type mice were conducted with approval from the Danish Animal Experiments Inspectorate under the permit numbers 2018-15-0201-01577 and 2020-15-0201-00767. Experiments with Lepr^Glp1r^KO mice and their controls were approved by the Institutional Animal Care and Use Committee at the University of Michigan (United States) under protocol number PRO00011066. The DREADD experiments were approved by local government authorities, Bezirksregierung Köln, Germany.

#### Xenium in situ gene expression analysis

Fresh frozen 10-12 μm tissue sections were placed on Xenium slides. The tissue was fixed and permeabilized according to the Xenium Fixation and Permeabilization Protocol (Demonstrated protocol CG000581). Probes were hybridized to the target RNA, ligated, and enzymatically amplified, generating multiple copies for each RNA target as described in Probe Hybridization, Ligation, and Amplification user guide (User guide CG000582). Xenium slides were then loaded for imaging and analysis on the Xenium Analyzer instrument, following the Decoding and Imaging user guide (User guide CG000584). Software analysis version 2.0.1.0 was used.

#### Immunostaining of brain tissue and image acquisition

Sections were brought up to room temperature (RT), air-dried, and slides were washed three times for 5 min each with phosphate-buffered saline (PBS) with Tween 20 (0.05%). Antigen retrieval was performed by incubating the slides in a solution of 0.3% NaOH and 1% H₂O₂ in PBS for 20 min. After two additional 5-min washes with 0.02 M potassium phosphate buffered saline (KPBS), slides were incubated in 0.03% glycine in KPBS for 10 min, followed by two more 5-min washes with KPBS. Blocking was carried out by incubating the tissues for 2 h at RT in a blocking solution containing 5% normal goat serum (NGS) and 0.3% Triton X-100 in KPBS, adjusted to a total volume of 700 µL per slide. Phosphorylated STAT3 antibody staining was performed in KPBS with 1% NGS and 0.3% Triton X-100 with rabbit anti-pSTAT3 (Cell Signaling Technology, #9145; 1:200) overnight at 4 °C. Slides were washed five times for 5 min each with 0.02 M KPBS. They were then incubated for 2 h at RT with the secondary antibody, goat anti-rabbit AF594, diluted 1:200 in KPBS containing 0.4% Triton X-100. The samples were then counterstained with DAPI (0.5 µg/mL in KPBS) and then coverslipped using 80 µL of VECTASHIELD antifade mounting medium (Vector Laboratories, #H-1000) before being stored at 4 °C. Images were acquired using a Axioscan Z1 scanning microscope with ×20 magnification. Individual sections were segmented and analyzed in QuPath (version 0.5.0) (Bankhead et al, 2017). Nuclei were segmented on DAPI signal and classified as pSTAT3(+) after training an object classifier on individually identified positive cells.

#### Fasting/refeeding experiment for snRNA-seq

Wild-type mice were fed a chow diet until 5 weeks of age and subsequently divided into a HFD and chow-fed group. At 20 weeks of age the chow-fed group was split into a group being fasted overnight and a fed group. The fasting started at 6 p.m. The next morning the mice either stayed fasted or were refed chow diet between 6.00 and 8.00 a.m for 90 min before they were sacrificed together with the other study groups. In summary, this study consisted of the groups: a DIO (15 weeks HFD), a chow-fed, a chow overnight fasted, and a chow refed group (90 min).

#### Leptin intraperitoneal injection experiments for snRNA-seq

For leptin intraperitoneal (IP) injections, 6-week-old wild-type mice were acclimatized for 2 weeks before injection. Mouse recombinant leptin (R&D Systems, #498-OB) was reconstituted to 1 mg/ml in 20 mM Tris-HCl pH 8.0 (Invitrogen, #10434742). IP injections were conducted with 3 mg leptin/kg mouse or a corresponding volume of 20 mM Tris-HCl pH 8.0. All mice were group housed and leptin and vehicle injections were randomized across cages and litters. To allow dynamic, temporal assessment of the transcriptional leptin response in neurons, arcuate nuclei were collected from mouse brains 1, 3, 6 and 24 h after leptin treatment. Mice were food deprived for 2 h prior to injections in the 1 and 3 h experiment.

#### MBH dissections

At sacrifice, brains were dissected and immediately placed in an ice-chilled coronal mouse brain slicer (Zivic Instruments, #BSMAS005-1, or ASI Instruments, RBM-2000C). A 1–1.5 mm thick coronal section was obtained between the rostral and caudal ends of Circle of Willis and placed in ice-cold PBS. Nuclei from the MBH of the left and right hemispheres were combined in a tube pre-cooled on dry ice and subsequently stored at –80 °C. All dissections were conducted between 8 and 12 a.m. to avoid transcriptional effects from circadian rhythm and feeding patterns.

#### DREADD stereotaxic surgical procedures

Male mice were anesthetized with isoflurane and received an IP bolus of Buprenorphine (0.1 mg/kg bodyweight), and were put into a stereotaxic frame (David Kopf Instruments). A local anesthetic agent (Lidocaine) was applied to the skin, the skull surface was exposed through a skin incision, and a small drill hole was made. For virus injections, the AAV was unilaterally delivered through a pulled glass micropipette into the caudal ARC/ventral DMH (coordinates from bregma: AP: –1.5 mm, DV: –5.5 mm, ML: ± 0.4 mm; AP: –2.1 mm, DV: –5.5 mm, ML: ± 0.4 mm;). Before waking up, mice received analgesic treatment (subcutaneous injection of Meloxicam, 5 mg/kg) for pain relief and were carefully monitored to ensure regain of pre-surgery weight. All animals were allowed 4 weeks for virus expression before starting the experiment.

#### Food intake studies in *Glp1r*^*Cre*^*;Trh*^*Dre*^ mice

AAV1/2-FLEX-FREX-hM3Dq (ETH Zurich, V892-1) was injected into the caudal ARC/ventral DMH of *Glp1r-ires-Cre;Trh-p2a-Dre* double transgenic mice at 8 weeks of age. Behavioral experiments were conducted four weeks post-injection. Food intake during the dark cycle was measured at 1, 2, and 3 h after the onset of the dark cycle. Thirty minutes before the dark cycle, mice received an IP injection of either 3 mg/kg Clozapine N-oxide (CNO, Hello Bio, Cat# HB6149) or saline. For the fast-refeed experiment, mice were housed in fresh cages without food at the onset of the dark cycle and were subjected to a 16-h fasting period. Food was reintroduced after 16 h of fasting and manually measured. CNO or saline was injected 30 min prior to food reintroduction. To investigate the orexigenic effects of ghrelin, CNO or saline was administered to fed mice at the onset of the light cycle. Thirty minutes later, 1 mg/kg of ghrelin was injected IP immediately before food measurement.

#### Immunohistochemistry of *Glp1r*^*Cre*^*;Trh*^*Dre*^ mice

For organ collection, mice were deeply anesthetized with ketamine and xylazine, and euthanized with transcardial perfusion of PBS, followed by 4% paraformaldehyde (PFA) in PBS (PFA-PBS). Brains were dissected, post-fixed at 4 °C in PFA-PBS for 16–22 h and then transferred to 20% sucrose in PBS. Brains were cut in 20 μm sections for immunostaining using a microtome. To cover the rostro-caudal axis of the brain region of interest, every fourth section was further processed (see below). The residual sections were collected in cryoprotectant and stored at −20 °C. Sections were blocked with 2% normal donkey serum in 0.4% Triton X-100 in PBS (NDS-PBST) for 1 h at RT and incubated with primary antibodies against HA (Rabbit HA Epitope Tag Antibody, ThermoFischer Scientific, 600-401-384) diluted 1:250 in NDS-PBST overnight at RT. Sections were washed with PBST and then incubated with secondary antibody anti-Rabbit IgG Alexa Fluor 555 (Life Technologies) diluted 1:1000 in PBS for 1 h at RT. After several washing steps with PBS, sections were mounted and counterstained with DAPI containing mounting medium (VECTASHIELD® Antifade Mounting Medium with DAPI, Cat# H-1200, Vector Laboratories). Brain sections were imaged by an slidescanner (Olympus) with 40x magnification using the Cy3 filter.

#### Lepr^Glp1r^KO in vivo experiments

DIO male and female Lepr^Glp1r^KO (19 weeks on HFD) and age-matched chow-fed male and female Lepr^Glp1r^KO and appropriate littermate controls, were used to analyze the role of leptin-mediated control of ghrelin-induced feeding. Mice were allowed at least 3 days of recovery between experiments. To assess body composition, mice were placed in an EchoMRI (Echo Medical Systems). % mass was calculated in respect to individual body weight. Statistical analyses were performed using two-way ANOVA. Probability values < 0.05 were considered statistically significant.

#### Lepr^Glp1r^KO ghrelin ± leptin feeding experiment

For the ghrelin ± leptin experiment, a cross-over study was performed in chow-fed male and female Lepr^Glp1r^KO and control mice where order of exposure to leptin (Novo Nordisk, 5 mg/kg) or an equivalent volume of 0.9% saline was randomized. 3 h after the onset of the light cycle, food was removed, and mice were injected with either leptin or saline. 1 h later, mice were injected with 1 mg/kg ghrelin IP (Hello Bio, HB2942) and food was returned. Food intake was measured at 24 h post-refeed.

#### Lepr^Glp1r^KO fast/refeed ± leptin experiment

A cross-over study was performed in chow-fed male and female Lepr^Glp1r^KO and control mice where order of exposure to leptin (Novo Nordisk, 5 mg/kg) or an equivalent volume of 0.9% saline was randomized. 3 days of baseline body weight and food intake were measured. On day −1, food was removed at the onset of the dark cycle and mice were injected with leptin or saline. The next morning, 3 h after the onset of the light cycle, mice received a second injection. One hour later, food was returned (after a 16-h fast), and food intake was measured at 24 h post-refeed.

### Lepr^Glp1r^KO ghrelin chow to HFD feeding experiment

Eight-week-old male and female Lepr^Glp1r^KO mice on a chow-fed diet were single housed, and twice daily food intake (following a 12:12 h light cycle) and body weight were measured for 3 days to establish a metabolic baseline. Mice were then switched to HFD and caloric intake and body weight were followed for an additional 10 days. Body composition was measured prior to onset of the study, and 1 week and 2 weeks post-switch to HFD. Bodyweight was then followed weekly to determine onset of DIO prior to subsequent experiments.

#### DIO Lepr^Glp1r^KO ghrelin feeding experiment

DIO male and female Lepr^Glp1r^KO and DIO control mice were injected with 0.9% saline (0.5 U/g) 4 h after the onset of the light cycle. Food intake was measured after 5 h. The following day, the animals were injected with 1 mg/kg ghrelin (IP) (Hello Bio, HB2942) and food intake was again measured after 5 h.

#### DIO Lepr^Glp1r^KO fast/refeed experiment

This study was performed in DIO male and female Lepr^Glp1r^KO or 8-week-old chow-fed male Lepr^Glp1r^KO mice, and appropriate diet-matched littermate controls. 3 days of baseline body weight and food intake were measured. On day -1, food was removed at the onset of the dark cycle. The next morning, food was returned after a 16-h fast and food intake was measured at 0, 1, 2, 4, (6), and 8.

### Immunohistochemistry of Lepr^Glp1r^KO mice

For immunohistochemical analysis of neuronal activation after stimulation with ghrelin, *ad libitum* fed DIO male and female Lepr^Glp1r^KO and DIO control mice were injected i.p. with 1 mg/kg ghrelin (Hello Bio, HB2942) or 0.9% saline (0.5 U/g). Ninety minutes post-injection, mice were anesthetized via isoflurane drop jar and euthanasia through transcardial perfusion of PBS followed by 10% formalin was performed. Brains were dissected and post-fixed in 10% formalin at RT for 4 h prior and then transferred to 30% sucrose in PBS. Brains were mounted on a freezing microtome and sectioned at 35 μm. Hypothalamic sections were rinsed 3× in PBS prior to blocking in 3% normal donkey serum in 0.3% Triton-X 100 in PBS (PBST) for 1 h at RT. Sections were then incubated overnight at RT in primary FOS (Cell Signaling Technology, 2250S 1:1000) or IBA1 (Fujifilm Wako, 019-19741 1:1000) antibody. Sections were then washed 3× in PBS and incubated in secondary Donkey anti-Rabbit 568 or 647 (Invitrogen, A-11057, A-31573 1:250) for 2 h at RT. Following a final 3x PBS wash, sections were mounted and coverslipped with Flouromount-G Mounting Medium (Southern Biotech, 0100-01). Imaging was performed on an Olympus BX53 microscope at 10x magnification using the cy3 or cy5 filter.

### Single-cell sequencing

#### Nucleus isolation

Frozen MBH samples from animals treated as detailed above were recovered in an ice-cold Nuclei EZ lysis buffer (Sigma, #NUC101), lyzed and homogenized using a KIMBLE 2 ml tissue dounce with pestle B (Sigma, #D8938) 10 times. The homogenate was incubated for 5 min including pipette mixing 1–2 times. Cell debris and myelin were removed by filtering the homogenate through 40 µm mini cell strainers (Pluriselect, #43-10040) followed by centrifugation of the filtrate (1000 rcf, 5 min, 4 °C). The pellet was resuspended in a 1:1 solution of EZ lysis buffer and 50% OptiPrep™ iodixanol (Sigma, #D1556). Subsequently, the 25% iodixanol solution with nuclei was layered on a 29% OptiPrep™ iodixanol solution (Sigma, #D1556). Nuclei in the iodixanol gradient were centrifuged (14,000 rcf, 22 min, 4 °C). The pellet was resuspended in nuclei buffer, PBS with 1% BSA, 2 mM Mg^2+^ and 40 U/µl Protector RNase inhibitor (Roche, #3335399001), and incubated for 15 min, before centrifugation (1000 rcf, 10 min, 4 °C). The final pellet was resuspended in 100 μl nuclei buffer for nuclei hashing.

#### Nuclei hashing and sorting

To enable multiplexing of samples, a unique hashtag antibody was selected for each sample in a multiplexing pool. TotalSeq™ anti-Nuclear Pore Complex Proteins Hashtag antibody 0.5 µg/µl (BioLegend, A0451-A0465, #AB_2861054 to #AB_2861068) was added to the 100 ul nuclei suspensions and incubated for 30 min. The nuclei were washed in nuclei buffer (1000 rcf, 10 min, 4 °C) and resuspended in nuclei buffer with 0.5 µg/ml DAPI. Single DAPI^+^ nuclei were sorted on a SH800 cell sorter (Sony Biotechnology) using the 70-μm Sorting Chip (Sony Biotechnologies, #LE-C3207) directly into the Chromium Next GEM Single Cell 3’ v3 or v3.1 reverse transcriptase reagent (10x Genomics) and were used immediately for library preparation. We aimed to overload the Chromium chip with approximately 28,000 nuclei per reaction to optimize the capture rate of nuclei.

#### Library preparation and RNA-sequencing

Single-nucleus RNA-sequencing libraries were generated using the Chromium Next GEM Single Cell 3’ kit, dual index, v3 or v3.1, with associated protocols (10x Genomics). The 10x Genomics protocols were followed up to cDNA amplification, where 1 μl 0.2 μM hashtag oligo primers (see Table EV7) were added to the cDNA amplification mastermix. The cDNA amplification and the following SPRI bead clean-up was performed as described in the 10x protocol. However, the pellet and the supernatant were saved in separate 0.2 ml tubes. The 10x protocol was continued without changes for the pellet to generate libraries from endogenous RNA. To generate libraries of the hashtag oligos, 70 µl SPRI beads (Beckman Coulter, #B23317) were added to the supernatant. The solution was mixed ten times and incubated at RT for 5 min. The tube was placed on a magnetic rack (10x Genomics) and the supernatant was discarded. The beads were washed twice on the magnet with 200 µl 80% ethanol, with 30 s incubation per wash. After the second wash, the tube was briefly centrifuged and placed on the magnetic rack again. Any remaining ethanol was removed and the beads were air-dried for 2 min. The tube was removed from the magnet and 40 µl EB buffer (Qiagen, #1014609) was added immediately. The solution was pipette mixed thoroughly and incubated at RT for 2 min. The solution was placed on the magnetic rack again and 39 µl supernatant was transferred to a new tube and the DNA concentration was quantified using a Qubit Fluorometer (Invitrogen, #Q32851). To add sample indices to the hashtag oligo library, 50 µl Kapa HiFi HotStart ReadyMix 2X (Roche Diagnostics, #07958935001), 2.5 µl 10 µM SI PCR primer (see Table EV7) and 2.5 µl 10 µM Illumina TruSeq BioL_D70X_s (see Table EV7) were added to 20 ng hashtag oligo DNA. A library preparation PCR was performed (a. 2 min 98 °C, b. 20 s 98 °C, c. 30 s 64 °C, d. 20 s 72 °C, repeated steps b.–d. 15 cycles in total, f. 5 min 72 °C, g. 4 °C hold). After the library preparation PCR, 120 μl SPRI was added to the sample, pipette mixed thoroughly and incubated for 5 min. The tube was placed on the magnetic rack and the supernatant was discarded. The beads were washed twice on the magnet with 200 µl 80% ethanol, with 30 s incubation per wash. After the second wash, the tube was briefly centrifuged and placed on the magnetic rack again. Any remaining ethanol was removed and the beads. The tube was removed from the magnet and 40.5 µl EB buffer was added immediately, pipette mixed and incubated for 2 min. The tube was returned to the magnet, 39 µl supernatant was transferred to a new tube and the DNA concentration was estimated with the Tapestation High Sensitivity 1000 DNA kit (Agilent, #5067-5584) and Qubit Fluorometer (Invitrogen, #Q32851).

#### RNA sequencing

Single-cell libraries of endogenous RNA and hashtag oligos were sequenced on SP and S2 flow cells using the Illumina NovaSeq 6000 platform (Illumina) with Read1 length of 28 bp, Index1 length 8 bp and Read2 length of 94 bp, to a depth of ~40,000 reads per cell for the endogenous RNA library and 10,000 reads per cell for the hashtag oligo libraries.

### Data processing and analysis

#### Sequencing and pre-processing

BCL files were first demultiplexed into FASTQ files using bcl2fastq v.2.19.01. RNA reads were first aligned to an optimized mm10 transcriptomic reference gtf file using Cell Ranger version 7.0 including intronic sequences (10x Genomics). CellBender (Fleming et al, 2023) was used to mitigate the effects of contaminating ambient RNA on our analysis. Hash tag oligo (HTO) libraries were quantified with kb count, Kallisto Bustools version 0.24.4 (Melsted et al, 2021). Quantified libraries were subsequently processed with Seurat (Hao et al, 2021). HTOs were normalized using the NormalizeData function with the normalization method set to CLR. A Gaussian mixture model with two components was fitted to each HTO distribution and a droplet was called to be positive for this HTO if it was predicted to belong to the cluster with the higher mean expression. Droplets positive for multiple HTO were classified as doublets. Inter HTO doublets were found using the recoverDoublets function from the scDblFinder package. Cells negative for HTO library as well as doublets were removed. Quality Control measures were calculated with AddPerCellQC(), Scuttle v1.4 (Bioconductor). Cells with too high or too low unique molecular identifiers (UMI) counts and intra- and intersample doublets were removed from the dataset. Genes expressed in less than 10 cells were removed.

#### Single-nucleus RNA-seq data analysis—initial processing

Downstream analysis was carried out in Seurat v4.3.0. For each 10X lane, cells with outlier UMI counts were identified and removed. Raw counts were normalized with the NormalizeData function, and principal component analysis (PCA) was performed with the RunPCA function using the top 3000 variable genes as input. Subsequently, clustering was performed with the FindNeighbors and FindClusters functions using the top 30 PCs as input. Cells were split into neurons and non-neuronal cells based on the expression of known marker genes using cellAnnotator (Kharchenko Lab). For the neuronal and non-neuronal datasets from the diet perturbation, 10X lanes were integrated using the FindTransferAnchor and IntegrateData functions using 30 PCs. PCA and clustering were then run as described above for the neuronal and non-neuronal atlas separately using the top 40 PCs.

#### Label transfer from reference single-cell datasets

To annotate cell types in our spatial and single-nucleus transcriptomics data, we performed label transfer from two independent reference datasets: a rabies-based hypothalamic cell atlas (Campbell et al, 2017) and a *Lepr*-expressing neuron dataset (Rupp et al, 2023). Both reference datasets were preprocessed using standard Seurat workflows, including normalization, variable feature selection, scaling, and principal component analysis (PCA). Label transfer was performed using Seurat’s FindTransferAnchors and TransferData functions, utilizing the first 30 principal components. For each reference dataset, we computed prediction scores representing the confidence of cell type assignments. Cells were assigned a particular identity only if their prediction score exceeded 0.6; cells below this threshold were marked as unclassified. This conservative approach ensured high-confidence cell type annotations. We validated the consistency of transferred labels by examining their correspondence with our independently derived spatial transcriptomics clusters. The resulting annotations provided two complementary perspectives on cell type identity, leveraging both the comprehensive coverage of the AgRP neuron afferent rabies-based atlas and the specialized focus of the LepR neuron dataset.

#### UMAP projection and cell type label transfer

To compare cell states across datasets while preserving the global structure of the reference data, we projected all datasets into the reference space defined by our nutritional perturbation atlas. We used the FindTransferAnchors() and MapQuery() functions to project query cells onto the reference UMAP embedding while simultaneously transferring cell type annotations.

#### Marker gene detection in spatial transcriptomics data

To classify cells as positive or negative for specific marker genes in our spatial transcriptomics dataset, we employed Gaussian mixture modeling. First, we performed centered log-ratio (CLR) normalization on the expression data to account for compositional bias. For each gene of interest, we analyzed the distribution of non-zero expression values using a two-component Gaussian mixture model implemented through the Mclust algorithm. This approach assumes that gene expression follows a bimodal distribution, with one component representing background/technical noise and the other representing genuine biological signal. Cells were classified as positive for a given gene if they belonged to the component with the higher mean expression level, as determined by the maximum likelihood estimates of the mixture model parameters. To validate the classification, we generated diagnostic plots showing the expression distribution overlaid with the fitted Gaussian components for each gene. This classification approach provided a systematic framework for identifying distinct cell populations in our spatial transcriptomics data while accounting for technical noise and probe diffusion.

#### Analysis of pSTAT3-positive cells

To assess differences in the proportion of pSTAT3-positive cells between dietary conditions across distinct spatial clusters, we employed a generalized linear mixed-effects model with a binomial distribution. The model included diet and cluster identity as fixed effects, with their interaction term, while accounting for animal-specific variation as a random effect. For each cluster, we computed total cell counts and the number of pSTAT3-positive cells. Estimated marginal means were calculated to test for diet-specific differences within each cluster, with *P* values adjusted for multiple comparisons using the Benjamini–Hochberg method.

#### Spatial differential expression analysis

To identify genes differentially expressed between dietary conditions, we performed statistical testing using the negative binomial test implemented in Seurat’s FindMarkers function. Raw counts were used for the analysis, with animal included as a latent variable to account for inter-individual variation. *P* values were adjusted for multiple testing, and genes were considered differentially expressed at an adjusted *P* value < 0.05.

#### Identification of a leptin gene signature using multi-study partial least squares analysis

To identify genes regulated by leptin across multiple experimental contexts, we performed an integrative analysis of three independent transcriptomic datasets profiling leptin responses. These included TRAP-seq data from leptin-treated hypothalamic *Lepr* neurons (GSE162603), RNA-seq from *Agrp* and *Pomc* neurons after leptin treatment, and RNA-seq data from nuclei of hypothalamic *Lepr* neurons after leptin treatment (GSE112125) (Inoue et al, 2019). For each RNA-seq dataset, we first performed quality control and normalization using DESeq2 v1.44.0. Raw count matrices were filtered to retain genes with at least 10 counts in a minimum of 2–3 samples (depending on the dataset). We then applied variance-stabilizing transformation (VST) to normalize the count data while accounting for the mean-variance relationship. To integrate data across studies while accounting for batch effects and technical variation, we employed multi-study partial least squares discriminant analysis (MINT-sPLS-DA) using the mixOmics R package. Expression matrices from all four studies were merged based on common gene names. The model was constructed using two components and selected the top 100 most discriminative features (genes) per component. Data were scaled and near-zero variance features were removed prior to model fitting. Sample groups (leptin-treated vs. control) and study of origin were provided as categorical variables to guide the analysis.

#### LGS scoring

LGS activity was computed using the AddModuleScore function in Seurat. For each leptin receptor-expressing neuronal population, we fit a linear mixed-effects models to test for differences in LGS activity across experimental conditions, while accounting for technical variation from cell hashing and sample preparation. The models included fixed effects for condition and random effects for sample ID and pool. Statistical comparisons between conditions were performed using estimated marginal means with Benjamini–Hochberg correction for multiple testing.

#### Quantification of perturbation effects

To quantify the effects of perturbations, we used scDist (v1.1.0), a statistical framework designed to identify changes in gene expression while accounting for technical and biological covariates. Prior to analysis, gene expression data was processed using the SCTransform (Seurat). We used the scaled expression matrix consisting of the top 5000 most variable genes as input and ran scDist with the following parameters: a minimum threshold of 5 counts per cell, treatment and hash-tag pool as fixed effects, and 10x lane identifier and sample as random effects. The analysis was conducted using 20 PCs.

#### Pseudo bulk differential expression analysis

We performed pseudo bulk differential expression analysis using EdgeR (v4.0.16) and limma-voom (v3.58.1) to identify transcriptional changes between experimental conditions while accounting for technical and biological variation. Raw count data from single-cell RNA sequencing was aggregated into pseudobulk samples for each animal cluster combination using the Seurat2PB() function.

Samples with library sizes below 10,000 counts were excluded. We retained genes that met minimum expression thresholds of at least 1 count in a minimum number of samples and a total count of at least 5 across all samples. Library sizes were normalized using the trimmed mean of M-values (TMM) method. For differential expression testing, we used limma-voom. The design matrix incorporated fixed effects for experimental group and other technical batch effects (hash pool). Statistical significance was assessed using moderated t-statistics with Benjamini–Hochberg correction for multiple testing. Results were compiled across all analyzed cell types.

#### Principal component analysis projection

To compare transcriptional states across datasets, we implemented a reference-based principal component analysis (PCA) projection approach. This method enabled direct comparison of cell states by projecting new data into a reference PCA space. For each cell type analyzed, we first identified shared genes between the reference and query datasets. The reference data was next log normalized and the top 5000 most variable genes were identified for PCA. The query dataset was independently normalized and scaled using the same set of variable features identified in the reference data. The scaled expression matrix from the query dataset was then projected into the PCA space defined by the reference data using matrix multiplication with the reference principal component loadings.

#### Diet classification

To determine whether ablation of *Lepr* altered the transcriptional response to HFD, we developed an approach to classify cells based on their transcriptional state. We first projected neurons from genetic knockout models into the principal component space defined by control animals fed either chow or HFD diets. We next trained a binomial logistic regression model using the first five principal components from the reference dataset to predict diet condition (HFD vs chow). The model was then applied to the projected data from knockout animals to generate probabilistic predictions of diet state for each cell. These cell-level predictions were aggregated by animal to generate a mean prediction score for each biological replicate. To assess the statistical significance of differences in prediction scores, we used a linear mixed-effects model. The model included fixed effects for dataset, genotype, and/or diet, and/or treatment. Post-hoc comparisons were performed using estimated marginal means with Tukey’s correction for multiple comparisons.

#### Statistical analysis

Statistical analyses were performed using R version 4.3.3 (R Core Team, 2018, https://www.rproject.org). Probability values < 0.05 were considered statistically significant. Unless otherwise mentioned, the Benjamini–Hochberg procedure was used to adjust for multiple tests.

### Experimental design and reproducibility

#### Sample-size determination

No statistical method was used to predetermine sample size. Sample sizes were selected based on previous studies using comparable experimental designs. Exact sample sizes are reported in the corresponding figure legends.

#### Blinding

Investigators performing image acquisition and quantification were blinded to experimental group. Blinding was not used for animal treatments or sequencing analyses.

## Supplementary information

Table EV1

Table EV2

Table EV3

Table EV4

Table EV5

Table EV6

Table EV7

Table EV8

Table EV9

Table EV10

Table EV11

Peer Review File

Source data Fig. 1

Source data Fig. 2

Source data Fig. 3

Source data Fig. 4

Source data Fig. 5

Source data Fig. 6

Expanded View Figures

## Data Availability

All processed single-cell and spatial transcriptomics data is available at https://zenodo.org/records/19564369. All raw single-nucleus RNA-seq data have been deposited in the EMBL-EBI BioStudies database under accession number E-MTAB-17544. All code can be found at https://github.com/perslab/Rausch-2025. The source data of this paper are collected in the following database record: biostudies:S-SCDT-10_1038-S44318-026-00914-w.
