# Supplementary material for: Leptin-sensitive hypothalamic Lepr+/Glp1r+ neurons limit hyperphagia and weight gain in diet-induced obesity
Source: EMBO J. 2026 Oct 2;45(21):7438–67. doi: 10.1038/s44318-026-00914-w (PMC13728182; doi:10.1038/s44318-026-00914-w)
Supplement: Supplementary file 1 [file 44318_2026_914_MOESM12_ESM.pdf]

THE EMBO JOURNAL

Leptin-sensitive hypothalamic Lepr+/Glp1r+ neurons limit hyperphagia and weight gain in diet-induced obesity

Dylan Belmont-Rausch, Benedicte Kapel, Abigail Tomlinson, Chao Ding, Alison Duensing, Bernd Coester, Cecilie Mathiesen, Leonie Cabot, Jenny Brown, Charlotte Kruse, Shad Hassan, Elisabeth Heaton, Christoffer Clemmensen, Michael Schwartz, Henning Fenselau, Martin Myers, and Tune Pers

Corresponding authors: Tune Pers (tune.pers@sund.ku.dk) , Martin Myers (mgmyers@med.umich.edu)

Review Timeline:

Submission Date:	6th May 26
Editorial Decision:	2nd Jul 26
Editor's Correspondence:	7th Jul 26
Revision Received:	18th Aug 26
Accepted:	4th Sep 26

Editor: Daniel Klimmeck

Transaction Report:

Please note that the manuscript was previously reviewed at another journal. As EMBO Press has a transfer agreement (including the identities of the referees) with that journal, revision was invited based on the reports from that previous external review.

(Note: With the exception of the correction of typographical or spelling errors that could be a source of ambiguity, letters and reports are not edited. Depending on transfer agreements, referee reports obtained elsewhere may or may not be included in this compilation. Referee reports are anonymous unless the Referee chooses to sign their reports.)

Point-by-point response to reviewers' comments:

Reviewer #1:

In this study the authors investigate the potential function of the lepR/Glp1r neuronal subpopulation suggesting that this population responds to leptin under diet-induced obesity and implying that leptin actively constrains further weight gain through its action specifically on this neuronal population. This is an important concept that can certainly impact how the action of leptin is interpreted in diet-induced obesity and that further support the original findings by Ottaway et al (Cell Metab 2015) showing that diet-induce obese (DIO) mice retain sensitivity to endogenous leptin.

Response: We thank Reviewer #1 for their insightful synposis.

Major:

1. This reviewer finds the first part of the manuscript with the molecular and associated bioinformatic analyses extremely elegant. It is less clear to this reviewer what new information is really brought by the in vivo studies attempting to relate the modulation of Lepr/Glp1r neurons with the modulation of AgRP neurons. The data provided through the chemogenetic study do not show that effects observed are due to the inhibition of AgRP neurons. This is implied, but there is no actual evidence provided by the authors in this sense. On the other side, the authors are certainly aware of recent literature showing that AgRP neurons are physiologically more active in the light rather than the dark phase (differently from what stated in the manuscript, please see Sayar-Atasoy et al., Nat Neurosci 2024 and also Douglass et al., Cell Metab 2025) and that this population is rapidly inhibited as soon as food becomes available (please see Chen et al., Cell 2015, but also the study by Garfiled et al., Nat Neurosci 2016, to which the authors refer), differently from what the authors suggest with the refeeding study. In particular, gabaergic DMH neurons (to which Lepr/Glp1r belong to) are known to be activated as soon as food becomes available and to inhibit AgRP neurons before actual food consumption (Garfiled et al., Nat neurosci 2016). It is unclear how the authors take into account the physiological regulation of AgRP neurons activity in response to light/dark cycle and fasting/refeeding in the context of the data provided in Figure 5 b-d.

Response: We thank the reviewer for these well-informed and thoughtful comments about two inter-related issues:

(1) Demonstrating the innervation and modulation of Agrp neurons by Lepr/Glp1r neurons. Given the wealth of published data demonstrating that *Lepr/Glp1r* neurons (by their various names: *Lepr/Trh* neurons, *Lepr/Bnc2* neurons, ARC/DMH *Glp1r* neurons, etc. (See Extended Data Fig. 4g)) innervate and inhibit *Agrp* neurons, we had not focused on demonstrating the innervation and inhibition of *Agrp* neurons by *Lepr/Glp1r* neurons in our previous submission. In addition to our previous DREADD

data using DREADDs demonstrating the restraint of *Agrp* neuron-associated feeding effects by the *Lepr/Glp1r* neuron population, we have now performed monosynaptic rabies tracing studies from ARC *Agrp/Npy* neurons to ARC and DMH *Lepr/Glp1r* neurons (Figure 4a-f). Additionally, to support our transcriptional data that suggests the leptin-mediated modulation of Agrp neuron activation state by *Lepr/Glp1r* neurons, we have now provided FOS studies, demonstrating the requirement for LepR signaling in *Lepr/Glp1r* neurons to restrain the ghrelin-mediated activation of *Agrp* neurons (Figure 6c-d).

(2) Understanding our findings with *Lepr/Glp1r* neurons fit with known the GABAergic DMH neuron-mediated control of Agrp neuron activity in response to circadian cues and food presentation. The reviewer correctly points out that *Lepr/Glp1r* neurons represent a subset of *Agrp*-innervation GABAergic DMH neurons. While many stimuli modulate the activity of *Agrp* neurons and *Lepr/Glp1r* neurons likely participate in the response to multiple stimuli, our analysis focuses specifically on understanding the role played by the unique leptin/DIO responsiveness of *Lepr/Glp1r* neurons- especially for the control of feeding. We have now restructured our manuscript to more clearly highlight this focus. Furthermore, we have explicitly placed our findings in the context of the overall control of *Agrp* neurons (Page 9).

2. Concerning the data presented in figure e-g, would the authors have evidence supporting constitutive increased activity of AgRP neurons by electrophysiology for instance? Are levels of AgRP mRNA significantly higher in the ko? Would then inhibition of AgRP neurons activity fully resolve the phenotype observed in these experiments?

Response: Our transcriptomic data reveal no change in the expression of *Agrp* or *Npy* in *Agrp* neurons from $Lepr^{Glp1r}$KO animals, suggesting that the expression of these genes may be controlled by direct LepR signaling in *Agrp* neurons, rather than indirect leptin action via *Lepr/Glp1r* neurons (now noted on Page 8). We have now provided FOS data to support the requirement for LepR signaling in *Lepr/Glp1r* neurons for the restraint of *Agrp* neuron activity, however (Figure 6c-d). We have also noted that we cannot rule out the possibility that *Lepr/Glp1r* neurons mediate their effects in DIO via other (non-*Agrp*) targets, however (Page 8).

3. This reviewer finds of particular interest the experiments attempting to link the function of Lepr/Glp1R neurons with the severity of DIO. Concerning the first set of experiments under fasting/refeeding, could the authors include the amount of weight loss of the animals? The body weight loss as % of body weight will be certainly higher in lean mice than in DIO control mice, which could help explain why lean animals are eating more in refeeding. Could the authors clamp the animals on the % of BW loss of DIO control mice and see if lean animals are still hyperphagic? And could the authors assess leptin levels in this body weight loss clamping experiment?

Response: The reviewer notes that DIO wild-type mice exhibit an attenuated hyperphagic response to overnight fasting compared to lean wild-type mice. The reviewer further suggests that the expected greater %body weight loss of lean animals in response to fasting could drive their increased hyperphagic response, given that DIO animals maintain higher adiposity and leptin concentrations than lean animals during a fast. As the reviewer

suggests, these factors likely limit the hyperphagic response to fasting in the DIO animals. The key observation here is that DIO fails to abrogate this hyperphagic response to fasting in the $Lepr^{Glp1r}$KO animals, demonstrating the requirement for direct leptin action on *Lepr/Glp1r* neurons for the DIO-mediated restraint of hyperphagia following a fast. Furthermore, because $Lepr^{Glp1r}$KO mice are more obese than wild-type animals, the restraint of post-fast hyperphagia by DIO requires the response to leptin but is not otherwise impacted by increased adiposity. We have now clarified these points in the text (page 7).

4. Data presented in figure 6 c-e: Based on the conclusion of the authors, would then the administration of a leptin receptor antagonist in association with the exposure to HFD fully recapitulate the phenotype of ko mice or not?

Response: LepR antagonists block leptin signaling in all Lepr neurons, not just *Lepr/Glp1r* cells. Although only *Lepr/Glp1r* neurons exhibit strongly increased LepR signaling during DIO, our pSTAT3 and transcriptomic data both demonstrate that DIO does not diminish LepR signaling relative to lean animals in any *Lepr* neuron population. Rather, most *Lepr* cell types exhibit a ceiling effect, such that DIO minimally augments LepR signaling in these cell types. Hence, the use of a LepR antagonist would block leptin action/LepR signaling in all *Lepr* neurons rather than uniquely preventing DIO-mediated increases in leptin action on *Lepr/Glp1r* neurons. Thus, LepR antagonist treatment would produce a global LepR-null like phenotype rather than specifically interrogating the effect of increased leptin signaling via *Lepr/Glp1r* neurons in DIO. In addition to adding clarifying language throughout, we now discuss this more fully in the manuscript (Page 4).

5. Figure panel 6d: the authors state in the discussion that if lepr/Glp1r neurons cannot detect and respond to rising leptin levels (as consequence of exposure to HFD) the rapid normalization of food intake is prevented. By looking at the data, however, one can conclude that the decrease in food intake on day 2 of HFD is blunted in the ko mice, rather than being prevented. This implies that mechanisms other than those hypothesize by the authors are relevant for the decrease in food intake in response to HFD after the initial hyperphagia in the ko mice. Could the authors comment on this?

Response: The reviewer is correct that while leptin signaling via *Lepr/Glp1r* neurons is required to fully blunt the initial hyperphagic response to HFD, the finding that food intake is partially reduced in response to progressive weight gain in $Lepr^{Glp1r}$KO mice suggests that additional processes must participate in this restraint of HFD-stimulated hyperphagia. We have now noted this, along with potential mechanisms that might underlie this finding, in the Discussion (Page 8):

6. Concerning the discussion section: why in the opinion of the authors this specific population of neurons remains sensitive to the action of leptin even in diet-induced obesity? Could the authors comment on the evolutionary value of preserving this sensitivity within this specific neuronal population?

Response: These questions represent crucial issues for our understanding of leptin action in obesity (and thus for future research). Framing the question in terms of why elevated leptin in DIO fails to increase LepR signaling in most *Lepr* neurons suggests some potential explanations. Certainly, we cannot blame LepR feedback inhibitors for the limited response

of most *Lepr* neurons to DIO, since these inhibitors are more strongly induced in *Lepr/Glp1r* neurons (consistent with the induction of these inhibitors by increased LepR signaling). *Lepr/Glp1r* neurons express more *Lepr* and exhibit greater responses to exogenous leptin than do other *Lepr* cell types, however, suggesting that *Lepr/Glp1r* neurons may sense and respond to increased leptin more effectively than other cell types (Page 8).

Regarding the potential evolutionary advantage of imposing a limit to the maximal leptin response in non-*Lepr/Glp1r* cell types: Leptin doesn't only restrain feeding but also increases sympathetic nervous system outflow (thereby increasing, e.g., blood pressure) and neuroendocrine function (thereby augmenting, e.g., thyroid function). Limiting the ability of leptin to increase these parameters beyond a certain point would prevent obesity and hyperleptinemia from promoting unwanted effects like hypertension and hyperthyroidism. In contrast, preserved leptin responsiveness in *Lepr/Glp1r* neurons augments the restraint of feeding in obesity. Indeed, the failure to appropriately restrain leptin action in obesity may underlie some obesity-related pathologies. We have now added this language to the Discussion (Page 8).

Additionally, it is possible that increasing only the indirect inhibition of *Agrp* neurons by leptin action on *Lepr/Glp1r* neurons (i.e., without increasing the effects of direct leptin action on *Agrp* neurons) in DIO might preserve some ability of important stimuli (e.g., cold exposure) to activate *Agrp* neurons and increase food intake during obesity This separation of function is consistent with the broader principle that distributed circuit architectures are more robust to perturbation than centralized ones (Marder, Nat Rev Neurosci. 2006, PMID: 16791145) (Page 9).

Minor:

In the methods: could the authors clarify in which experiments were females used? the impression is that most data were actually obtained in males. Also were the animals singly housed? group housed? please provide further details on housing.

Response: While our previous data were mostly obtained in males, we have now included females in all experiments except for the microgliosis experiment. Animals were group housed except during the feeding studies shown in Figure 5-6.

In the methods: the first sentence in the paragraph describing the nucleus isolation is unclear. What do the authors mean with "samples were selected in pools of 4-12 randomized samples across developmental time"? also at what time of the day were the tissues collected for the molecular analyses?

Response: This wording was carried over from the methods describing a different experiment (not part of this study). We have removed this wording. All collections were performed between 8 AM-noon.

In the methods: can the authors clarify for all fasting/refeedinge experiments, when exactly were the animals fasted/refed? When exactly in the dark/light cycle?

Response: Fasting began with food removal immediately prior to lights off and continued overnight (~14 hours), after which refed animals received the appropriate diet. For snRNA-seq, animals were euthanized for tissue collection 90 minutes after refeeding. For the measurement of refeeding, food intake was monitored over the subsequent 8 hours.

In the methods: what statistical approach was used for the analysis of the chemogenetic studies reported in Figure 5?

Response: We used a linear mixed model effects model with treatment as a fixed effect and mouse as a random intercept to account for the repeated-measures structure. Post hoc tests were performed to evaluate significance at each time point. We have updated the Figure Legend accordingly.

Reviewer #3:

Summary

Belmont-Rausch et al. combined spatial transcriptomics and single cells sequencing with pSTAT3 immunostaining in hypothalamic tissues to identify an Arc/DMH cell type marked by expression of Lepr and Glp1r. They then study the response and function of these and AGRP neurons expressing LepR in DIO mice and after leptin treatment or fasting. They report significant enrichment of pSTAT3 in these neurons relative to other Lepr+ cells in DIO mice at baseline and based on this, the authors hypothesize that these cells retain leptin sensitivity in DIO mice to restrain food intake. They suggest that this potentially explains why leptin-resistant DIO animals weigh less than db animals with a global LepR mutation.

Response: We agree that *Lepr/Glp1r* neurons exhibit increased leptin sensitivity (including in DIO), and that this feature augments the restraint of food intake in DIO. We apologize for causing confusion by not being clearer about the exact nature of impaired LepR signaling in non-*Lepr/Glp1r* neurons during DIO, however. LepR signaling (pSTAT3 and transcriptional responses to leptin) is not decreased in DIO compared to lean animals for any population of *Lepr* neurons. Rather, LepR signaling in all *Lepr* cell types tends to increase in DIO, even though this trend is not significant in most cell types, and the increased LepR signaling exhibited by *Lepr/Glp1r* neurons in DIO is stronger and more significant than for other neuron populations. Hence, baseline leptin signaling is preserved for all *Lepr* neurons in DIO, but the unique sensitivity of *Lepr/Glp1r* neurons to elevated leptin/DIO effectively increases LepR signaling specifically via this single circuit. Consistent with ongoing baseline LepR signaling in all Lepr neurons, DIO $Lepr^{Glp1r}$KO animals remain less obese than *db* animals (albeit more obese than DIO controls) and are protected from other *db*-like phenotypes such as infertility. We have clarified this issue throughout, explicitly noting this information on Page 4.

To further evaluate this, they first perform an enormous number of spatial transcriptomic set of single-nucleus RNA-seq experiments across a spectrum of metabolic conditions. They report that feeding mice on HFD alters gene expression in Lepr+/Glp1r+ cells to a greater extent than in leptin-responsive cell types with genes associated with increased neural activity being upregulated and genes associated with decreased neural activity being downregulated. The authors then used an intersectional strategy in Glp1r-Cre and Trh-Dre transgenic mouse model to introduce a Gq Dreadd and showed that activation of these cells reduced food intake after a fast and blunted ghrelin-induced food intake. Finally, they fed mice with a

Lepr knockout in Glp1r neurons a high-fat diet and showed that the animals were more hyperphagic with increased body weight and fat mass vs control mice fed a high-fat diet. The authors conclude that the Glp1r+/Lepr+ cells represent a key cell type through which leptin constrains further weight gain in DIO mice.

Response: We thank Reviewer 3 for the apt summary of these findings.

However, while the (voluminous) data in this paper is of interest, the functional tests of the authors' key conclusions are extremely limited and are insufficient to support their conclusions. Thus, the first four figures present dense data sets showing changes in gene expression under numerous different conditions in different animals. While these data are of interest, their main purpose is not to generate lists but rather to generate hypotheses that can then be tested rather than being an end unto themselves. However, the following functional studies in the last two figures are extremely limited with only a single new finding. These include a demonstration that activating GLP1R/LepR neurons suppresses feeding. However, a similar finding was reported in Webster et al (Nature Metabolism, 2024) for Trh neurons and also Bnc2 expressing neurons in the same regions of the hypothalamus that have many perhaps all of the same properties of GLPR/LepR neurons reported here. It seems likely that the papers are referring to the same population and if this is the case the data in this figure is not novel. Of course if these populations are different from the cells reported here, then the authors need to establish this and integrate their findings into these previous ones. The authors also show increased food intake and body weight in LepR KO in GLP1R neurons in chow and DIO mice. Increased weight in chow-fed animals with a LepR KO in GLP1R neurons was also reported previously from the same group, see Rupp et al (Journal of Clinical Investigation, 2023). Thus, the only new functional data in this paper is the demonstration that these animals consume more food and gain more weight on a HFD than do controls without the knockout (Fig. 6).

Response: The reviewer is correct that others and we previously showed that *Lepr/Glp1r* neurons (which are essentially the same as *Lepr/Trh* and *Lepr/Bnc2* neurons, as noted) are crucial for the control of energy balance by leptin and that activating these neurons suppresses food intake. The reviewer is also correct that our results provide new evidence in support of these conclusions. In Extended Data Fig. 1e and 2 (and below) we show, using label transfer and gene expression dot plots, that, that the *Lepr/Glp1r* Neurons described in our paper map onto neurons the L12.Glp1r/Ebf1 cells described in Webster et al. (Nat. Metab. 2024, PMID: 39578647), Rupp et al. (JCI 2023; PMID: 37581939) and the Bnc2 cells described in Tan et al. (Nature 2024, PMID: 39478220).

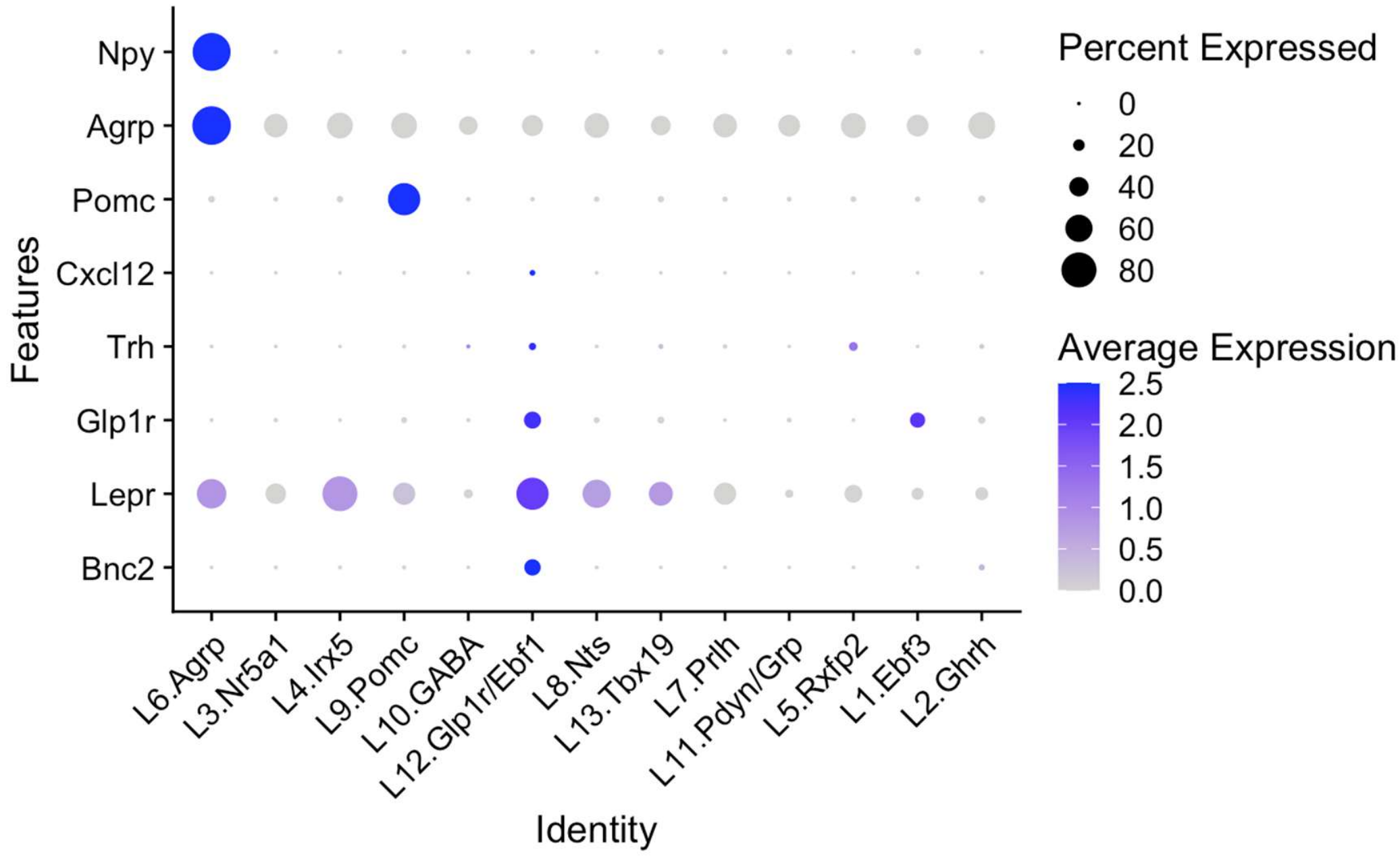

Extended Data Figure 2. Expression of *Bnc2*, *Cxcl12*, *Glp1r*, *Lepr*, and *Trh* across mediobasal hypothalamic neuronal populations identified in Rupp et al. The canonical markers *Agrp*, *Pomc*, and *Npy* are shown for reference. The L12.Glp1r/Ebf1 cells correspond to the *Lepr/Glp1r* population. Dot size denotes the percentage of cells expressing each gene within a population, and color intensity reflects scaled average expression.

We respectfully disagree with the reviewer that the known importance of the *Lepr/Glp1r* neuron population for the physiologic control of food intake and body weight negates the importance of our findings that: (1) *Lepr/Glp1r* neurons remain uniquely leptin-responsive in DIO; (2) that this property of *Lepr/Glp1r* neurons enables hyperleptinemia to blunt hyperphagic responses to fasting in ghrelin, including in DIO; and (3) that leptin responsiveness in *Lepr/Glp1r* neurons restrains the hyperphagic response to HFD.

Additionally, we now provide a new computational analysis of published connectomics data (Webster et al., Nat. Metab. 2024, PMID: 39578647), revealing that *Lepr/Glp1r* neurons represent the dominant leptin-sensitive inhibitory input to *Agrp* neurons. Furthermore, we provide new data showing that the leptin acts via *Lepr/Glp1r* neurons to blunt *Agrp* neuron activation in DIO (Figure 6c-d). We have also provided new data revealing that leptin action via *Lepr/Glp1r* neurons underlies potential maladaptive responses to DIO, including hypothalamic microgliosis (Figure 6e-f).

We hope that the reviewer will agree that these data represent more than a single novel finding and that together these findings provide a new understanding of leptin action in DIO.

But even this finding is limited insofar as the animals were monitored for only 7 days at which point the control mice weighed ~24g and the knockouts weighed ~27g. While there did appear to be a larger difference in food intake during the one week study, the weight of the groups at the start of the study is not properly with the wild type mice weighing ~ 2g less than the knockout mice (22g vs 24g) . The weight gain of ~2g in the controls on a HFD is not very different than the ~3g weight gain for the KO mice. In addition the

total weight gain in both groups seems lower than is generally seen perhaps because the diet was changed when the mice were relatively young. A disparity in the initial body weight could influence leptin sensitivity across multiple neuronal populations, including Glp1r/Lepr, AGRP, and POMC neurons, potentially confounding the interpretation of the results.

Response: The reviewer suggests that longer-term data are required to fully understand the mechanisms underlying the response to HFD in $Lepr^{Glp1r}$KO and control animals, including whether the increased baseline adiposity itself might prevent $Lepr^{Glp1r}$KO mice from responding appropriately to HFD. We have now provided these longer-term data (Extended Data Figure 8f-h).

While we agree that body weight differences can influence leptin sensitivity, several observations suggest that this mechanism does not underlie our results. First, the initial (1-2 day) hyperphagic response to HFD was identical between genotypes, arguing against a generalized difference in leptin sensitivity at baseline. Second, the phenotype we observe, a specific failure to normalize intake over days, is precisely what is predicted by removing the ability of *Lepr/Glp1r* neurons to respond to rising leptin, not by a nonspecific shift in leptin sensitivity. Third, this result is not interpreted in isolation: it converges with the DREADD sufficiency data, the leptin requirement experiments in DIO, the restored ghrelin sensitivity, and the FOS and transcriptomic evidence, all of which independently support the same conclusion.

Furthermore, our longer-term data reveal that approximately 2 weeks after the initial enhanced hyperphagia and weight gain trajectory provoked by HFD in $Lepr^{Glp1r}$KO mice, the weight gain trajectory of these animals flattens to parallel that of control mice. In contrast, control animals remain on a constant weight gain trajectory for the 60-day duration of the study, despite having exceeded the baseline adiposity of $Lepr^{Glp1r}$KO mice before the end of the first week of HFD feeding. If the baseline weight difference were driving the result, one would expect the groups to continue to diverge, or for control animals to exhibit accelerated weight gain once they achieve the threshold adiposity for accelerated weight gain.

Not only do these data argue that increased adiposity itself does not impact the trajectory of weight gain on HFD, but they reinforce the conclusion that the genetic lesion in $Lepr^{Glp1r}$KO mice underlies the failure of these animals to rapidly normalize food intake and the rate of body weight accretion following the switch to HFD.

Because the weight gain trajectory of $Lepr^{Glp1r}$KO mice eventually normalizes, increased leptin action via non-*Lepr/Glp1r* neurons and/or other mechanisms resulting from increased adiposity must ultimately suffice to restrain the response to HFD, albeit only following the establishment of a much-increased body weight and adiposity in the $Lepr^{Glp1r}$KO mice.

Moreover if the authors' hypothesis is correct, the weight of the knockout animals on a high fat diet should be similar to that of db/db mice which does not appear to be the case, at least at the time point when they terminated the study. If these knockout mice do not ultimately replicate the phenotype of db mice, it would by necessity indicate that other populations also restrict further weight gain in DIO mice. Thus, the limited experiments that are presented also do not exclude the possibility that other leptin-responsive cell types also retain activity and limit further weight gain in DIO mice. Feeding mice with LepR knockouts in other populations a HFD would be required to buttress this conclusion.

Response: Again, we apologize for not being clearer previously that we do not propose that *Lepr/Glp1r* neurons are the only neurons that exhibit LepR signaling in DIO, but rather that

they are unique in the sensitivity and magnitude of their response to elevated leptin in DIO. Other *Lepr* neuron populations continue to exhibit LepR signaling in DIO, but at levels similar to or only slightly higher than those observed in chow-fed mice. Hence, we would not expect $Glp1r^{Lepr}$KO mice to fully recapitulate the phenotype of db/db mice, which lack all LepR signaling in all cell types. We have clarified our language throughout, and explicitly noted this issue on Page 4.

Importantly, we claim not that *Lepr/Glp1r* neurons account for all leptin action in DIO, but rather that they are the principal population through which leptin can augment LepR signaling and the restraint of feeding in obesity. Hence, the effects of *Lepr/Glp1r* neurons play a disproportionately large role in the response to hyperleptinemia, as in DIO.

Other straightforward studies that could further support their hypothesis were not done. The intersectional targeting that the authors employed (ie Glp1r-CrexTrh-Dre) could also be used to inhibit GLP1R/LepR neurons with chemogenetics or ablate these neurons specifically in the adult DMH/ARC region. The authors' hypothesis suggests that this would increase the weight of mice fed a HFD and that they should ultimately resemble db/db mice. This experimental strategy would likely provide stronger support for their hypothesis compared to the genetic approach they used to knock out Lepr in Glp1r-expressing neurons, since the observed effects in that model could be due to developmental deficits or to GLP1R/LepR neurons located in other brain regions, such as the hindbrain, rather than specifically in the DMH/ARC.

Response: As noted above, roles for the unique leptin sensitivity of *Lepr/Glp1r* neurons (including in DIO), rather than the function of the neurons themselves, represent the focus of this manuscript. Hence, our limited hM3Dq-based studies focused on demonstrating that these neurons can promote the effects that we set out to examine in our leptin/DIO-focused analysis. The effects of manipulating leptin responsiveness in these neurons are quite dramatic.

We agree that it would be interesting to examine the effect of acutely inhibiting these neurons, however. Unfortunately, this requires developing and employing a system (that does not currently exist) with which to silence neurons using intersectional genetics to specifically target *Lepr/Glp1r* neurons via the Glp1r-Cre;Trh-Dre strategy. To this end, we generated a novel Cre/Dre-dependent inhibitory hGlyR (human glycine receptor) AAV for intersectional silencing. Unfortunately, despite extensive attempts at optimization, this virus failed to produce expression sufficient for reliable neuronal silencing, precluding its use in the experiment. Because this approach requires generating a novel viral tool that can overcome these technical challenges (rather than using an existing, validated construct as for the excitatory DREADD) we believe that this is beyond the scope of the current study.

There are also issues with the studies of feeding driven by AGRP neurons or ghrelin. In Fig. 5, the data show that activating Glp1r/Trh neurons suppresses feeding in the dark cycle, post-fasting, and after ghrelin injection. The claim in the text that "activation of Trh/Glp1r neurons blocks AGRP neuron-dependent feeding effects" is overstated. While AGRP neuron activation is known to drive feeding in these contexts, this does not equate to demonstrating that Glp1r/Trh activation directly opposes or blocks AGRP neuron-driven feeding. To substantiate this conclusion, additional experiments would be necessary—for example, co-activating Trh/Glp1r and AGRP neurons simultaneously to test whether the former

can attenuate AGRP-induced feeding, a scenario that would seem unlikely, as AGRP neurons are thought to function downstream of Trh/Glp1r neurons. Indeed, previous studies have shown that activation of TRH neurons does not block AGRP neuron-induced feeding behavior (Webster et al., 2024 Nature Metabolism). Similarly, the claim that "leptin acts via Lepr/ Glp1r neurons to limit AGRP neuron-driven feeding effects" (Figures 5e-g) is not adequately supported. As shown in Figure 5e, Glp1r(Lepr KO) mice are significantly more obese than controls, with a 3- to 4-fold increase in fat mass. It is thus possible that Agrp neurons in the KO mice are less sensitive to leptin as compared to control mice raising the possibility that the observed lack of leptin-induced suppression of food intake, or attenuation of ghrelin-induced feeding, in the KO mice could be due to intrinsic leptin resistance within AGRP neurons rather than a functional consequence of disrupting Glp1r/Lepr -AGRP circuits. To properly support the authors' conclusion, a pair-fed control group would be necessary to match body weight prior to leptin or ghrelin administration, thereby controlling for possible difference in leptin sensitivity secondary to differences in weight.

Response: We thank the reviewer for this important point. Dark-cycle intake, post-fast refeeding, and ghrelin-induced feeding each require Agrp neuron activation (Krashes et al., 2011, PMID: 21364220; Wang et al., 2013, PMID: 24567905; Douglass et al., 2025, PMID: 39719709).

While we now also show that LepR signaling in *Lepr/Glp1r* neurons is required to restrain the activation (measured by FOS) of *Agrp* neurons in DIO mice (Figure 6c-d), it remains possible that *Lepr/Glp1r* neurons mediate leptin responses via additional (non-*Agrp*) downstream circuits. Indeed, in other studies, we find that *Lepr/Glp1r* neurons innervate some non-*Agrp* neurons (data not shown). Hence, we have more carefully described the potential roles for *Agrp* neurons in the observed effects of *Lepr/Glp1r* neurons and explicitly state that the modulation of non-*Agrp* neurons may contribute to the effects of *Lepr/Glp1r* neurons (page 8).

Regarding potential impacts of leptin resistance non-*Lepr/Glp1r* neurons secondary to the baseline increased adiposity of $Lepr^{Glp1r}$KO mice: In addition to the arguments already presented above, please note that hypothetical obesity-induced leptin resistance in *Agrp* neurons does not represent the most parsimonious explanation for the phenotypes of $Lepr^{Glp1r}$KO mice (in which removal of LepR creates absolute leptin resistance in *Lepr/Glp1r* neurons). Furthermore, our transcriptomic data reveal no decrease in LepR signaling in *Agrp* neurons in DIO or in $Lepr^{Glp1r}$KO mice. Furthermore, while pair feeding of DIO $Lepr^{Glp1r}$KO mice to DIO control mice could be used to match their body weights, this would also place the $Lepr^{Glp1r}$KO mice in a state of chronic fasting. The fasted state would increase food intake in the $Lepr^{Glp1r}$KO mice during the measurement of feeding, exacerbating (rather than blunting) the already robust response to ghrelin in the DIO *Lepr/Glp1r* neurons.

The authors surmise that leptin signaling is retained in the GLPR/LepR neurons of DIO mice but do not directly test this. Do GLP1/LepR neurons respond to leptin ex vivo in hypothalamic slices prepared from DIO mice in contrast to AGRP or POMC neurons which no longer respond? In addition, the authors could follow the activity of these neurons using Ca++ imaging over time in control mice vs mice with a LepR KO in GLP (or other) neurons fed a high fat diet. If the author's hypothesis is correct, the GLPR/LepR neurons should retain full activity while the activity of AGRP neurons should

decrease.

Response: While this comment suggests that we have not directly tested leptin signaling, it appears to refer to neuronal activity, instead. Note that both our pSTAT3 data and our transcriptomic data demonstrate that LepR signaling is increased in *Lepr/Glp1r* neurons from DIO mice (Figures 1-2). Others have also demonstrated the continued leptin responsiveness of some DMH Lepr neurons in DIO (Enriori, J Neurosci, 2011, PMID:21849525; Rezai-Zadeh, J Neurosci, 2014, PMID:24048859).

While our transcriptomic data suggest the increased activity of *Lepr/Glp1r* neurons in DIO (Figure 2e), the reviewer is correct that we have not directly examined the activation state of these cells. While our transcriptomic data, the *Lepr/Glp1r* neuron-dependent restraint of *Agrp* neuron activity (Figure 6c-d), and the circuitry of Lepr/Glp1r neurons (Figure 4a-f) strongly suggest the increased activity of *Lepr/Glp1r* neurons in DIO, we have carefully avoided making definitive claims of this nature. Again, our experimental thrust in this manuscript focuses on roles for the unique leptin sensitivity of *Lepr/Glp1r* neurons for the control of food intake and body weight, rather than mechanisms that may control the firing of these cells.

In addition:

Numerous papers have shown that pre-treatment with gut peptides such as GLP1, a GLP1/glucagon dual agonist, amylin, CCK, and others restore leptin signaling and that coadministration yields synergistic effects. Even if the GLP1R neurons retain activity in DIO mice this suggests that these peptides can restore signaling in these or other neurons. This would appear to vitiate the authors' assertion that trying to resensitize other populations to leptin would be futile.

Response: We are somewhat confused by this comment, given that we previously asserted that it could be useful to resensitize the *Lepr* circuits that poorly respond to excess leptin in DIO. From page 10 of the previous version of the manuscript:

"In addition to reshaping our understanding of obesity pathogenesis, these results thus suggest that there may be little point to targeting the already appropriately active Lepr/Glp1r→Agrp neuron circuit for the therapy of obesity, but rather that it may be more effective to intervene at the level of the leptin-regulated circuits that fail to respond to increased leptin."

We have now attempted to clarify our meaning (Page 9), however.

The paper suggests that AGRP and other leptin-responsive neurons are more strongly activated in lean animals but that the GLP1R/LepR neurons play a greater role in DIO mice. This raises the possibility that these neurons may require higher leptin concentrations for leptin signaling (though they clearly contribute to the leptin response in chow-fed animals). The authors neither consider this possibility nor do they perform any experiments to explore it.

Response: We apologize for any confusion we may have caused with our previous wording. We do not claim that *Lepr/Glp1r* neurons respond poorly to leptin at lower leptin concentrations, just that they respond robustly to increased leptin concentrations. Indeed, several lines of evidence demonstrate that *Lepr/Glp1r* neurons actively respond to leptin at

physiological (chow) levels: (1) *Lepr/Glp1r* neurons exhibit significant pSTAT3 and leptin gene signature (LGS) expression in chow-fed animals; (2) LGS expression in *Lepr/Glp1r* shifts progressively across nutritional states (Fast → Refeed → Chow → HFD), indicating graded, not threshold-dependent, regulation; (3) LepR ablation in Glp1r^{Lepr}KO mice abolishes LGS (and other leptin-dependent changes in gene expression) in *Lepr/Glp1r* neurons in chow-fed mice, demonstrating ongoing LepR signaling in these neurons in lean mice. Hence, rather than suggesting a rightward-shifted dose–response for leptin signaling in *Lepr/Glp1r* neurons, our findings support a model in which *Lepr/Glp1r* neurons respond to endogenous (and exogenous) leptin across the full range of leptin concentrations tested. Their ability to maintain their responsiveness into DIO represents the unique property of this cell population.

The authors report increased pStat3 levels in GLP1/LepR neurons in DIO mice and assume but do not show that this is driven by leptin. What is the level of pStat3 in DIO mice with a LepR KO in GLP1R neurons?

Response: Over two decades of data from a host of laboratories has established that hypothalamic pSTAT3 in untreated lean and obese mice, as well as in leptin-treated animals, results from cell-autonomous leptin-LepR signaling (Vaisse, Nat Genet, 1996, PMID: 8782827; El-Haschimi, JCI, 2000, PMID: 10862798). Furthermore, we have previously shown the absence of pSTAT3 in Glp1r-expressing hypothalamic neurons in Glp1r^{Lepr}KO mice (Rupp, J Clin Invest, 2023, PMID:37581939), consistent with the requirement for LepR to promote pSTAT3 in these cells.

Numerous papers including from one of the authors, and others, have shown that prolonged exposure to leptin can feedback inhibit leptin signaling. For example, exposure of 293 cells expressing LepR to prolonged leptin treatment leads to a downregulation of leptin signaling, see Dunn et al Mol Endo 2005. In addition, a leptin response also becomes attenuated in NPY-expressing hypothalamic neurons expressing NPY treated with high-dose leptin, see Dhillon …. Belsham, Endocrinology 2012. Finally mutations in Tyr985 of the leptin receptor in vivo result in leanness (Bjornholm….. Myers, JCI 2007) which the authors suggest is a result of the loss of autoinhibitory signals. A core question is why don't the GLP1R/LepR neurons behave similarly to these other neurons. Why does SOCS3 which is still induced in these neurons in DIO mice not inhibit leptin signaling? Is Tyr985 no longer engaged? A further exploration of the mechanism would elevate the importance of this paper.

Response: To clarify, we do not argue that feedback inhibitors play no role in modulating the amplitude of LepR signaling (they clearly do). Indeed, we expect that they decrease the amplitude of LepR signaling in all *Lepr* neuron types, including *Lepr/Glp1r* neurons. Rather, because *Lepr/Glp1r* neurons express higher levels of these leptin-stimulated genes (e.g., *Socs1, Socs3, Cish, Ptpn1, Ptpn2*) than other *Lepr* cell types following leptin treatment or DIO, these inhibitors cannot account for the failure of non-*Lepr/Glp1r* neurons to respond to elevated leptin. As requested here and by the other reviewer, we have now commented potential mechanisms by which *Lepr/Glp1r* neurons may maintain their unique sensitivity to hyperleptinemia/DIO (Page 8).

Minor suggestions:

1. Fig. 1f, legends for each color code should be added either in the graph or the legend text. Additionally, it appears that the majority of Glp1r/LepR co-expressing neurons are located in the DMH, with relatively few in the ARC. The authors should provide quantification to support this observation—specifically, the number or percentage of these neurons found in the DMH versus the ARC. This is particularly important given that previous studies (Kim et al, Science, 2024. PMID: 38935778; Rupp et al, Journal of Clinical Investigation, 2023) have reported LepR- and GLP1R-expressing cells to be largely distinct within the ARC.

Response: We have previously shown that the *Lepr/Glp1r* population is distributed across the ARC and DMH (Rupp, J Clin Invest, 2023, PMID:37581939). The methods to detect *Lepr/Glp1r* neurons used here differ from our previous study, however, and may account for differences in the apparent balance of distribution for these neurons. The legend is shown on the right-middle part of the figure.

2. Fig. 2h, why are there significantly fewer genes for Pomc compared to the other two cell types?

Response: Fewer genes were regulated by diet in *Pomc* neurons than in other cells, so there were fewer to show in this graph. Note that we have moved this panel to Extended Figure 5 as part of the requested reorganization and streamlining of the revised manuscript.

3. Fig. 2i, a GO analysis for DEGs from each group-associated cluster should be included in the supplementary figure.

Response: Results from this GO analysis can be found in supplementary table 6

4. In Figure 3a, the figure appears to show analysis from the ARC region, while the text refers to the MBH (line 237). The authors should clarify which specific region was analyzed. If it is indeed the ARC, they should report how many cells were found to co-express LepR and Glp1r. This quantification is critical, especially in light of Figure 1f, where it seems that the majority of Glp1r/LepR co-expressing neurons are located in the DMH, with relatively few in the ARC.

Response: We thank the reviewer for noting this discrepancy. We have updated the schematic.

5. Fig. 3 e, f, a similar analysis should be performed for POMC cells.

Response: The reviewers both commented on the density of the transcriptomic data in our initial submission. We thus removed this analysis to streamline the manuscript.

6. Fig. 4a, an analysis of Glp1r+ and Lepr+ cells distribution in other brain regions should be included in the supplementary figure. The authors may consider using publicly available ABA atlas datasets https://portal.brain-map.org/atlases-and-data/bkp/abc-atlas.

Response: We have previously examined the distribution of *Lepr/Glp1r* co-expressing neurons across the brain (Rupp, J Clin Invest, 2023, PMID:37581939) and found no significant numbers of these cells outside of the hypothalamus

7. Fig. 5a, are there controls for Glp1r+, Trh- cells or Trh+, Glp1r- cells using a Cre-on, Dre-off strategy to express DREADDs? This is essential to demonstrate the cell type specificity.

Response: Cre-on/Dre-off exclusion controls are in our hands not currently feasible, validated reagents for these lines are not yet available, and our attempts to engineer them have not yielded reliable/selective expression. To address specificity, we combined Cre/Dre targeting with histological verification, a saline-CNO crossover, and the *Lepr* KO genetics showing convergent behavioral effects.

8. Fig. 5b-d, it is recommended to administer CNO injections in a separate group of mice expressing Con/Don-fluorescent protein (GFP/mCherry) since CNO itself has noticeable metabolic side effects.

Response: We and others have previously shown in a host of studies that CNO does not alter food intake in animals that do not express DREADDs in appetite-regulating neurons. See for example: Cheng, JCI Insight, 2020, PMID: 32191640.

9. Fig. 6b, ANOVA-based group effects significance levels should be denoted in the graph.

Response: We have added the overall interaction statistics from the linear mixed-effects models (Type II Wald χ^2 tests) directly to the relevant panels. For the fasting-refeeding experiment (Fig. 5d), the genotype × diet interaction ($\chi^2 = 3.88$, $p = 0.049$) is now annotated, confirming that diet-induced blunting of refeeding intake differs by genotype. For the ghrelin response in DIO (Fig. 5g), the genotype × treatment interaction ($\chi^2 = 8.22$, $p = 0.004$) is displayed alongside the post-hoc within-genotype contrasts. See revised Figure 5.

10. Line 639-641, the authors note that feedback inhibitors failed to block LepR signaling in Lepr/Glp1r cells. A recent study suggests that increased mTOR activity blocks leptin signaling in DIO mice. It is possible that these cells have relatively lower mTOR activity compared to other cells in DIO mice, rendering them sensitive to leptin. The authors should note this potential mechanism in the discussion. Additionally, it would be valuable to analyze gene expression related to the mTOR pathway and consider future experiments to probe mTOR activity in these cells under chow and DIO conditions.

Response: We thank the reviewer for this suggestion. We quantified mTOR-pathway gene expression across *Lepr*-expressing neuronal subtypes under chow and HFD conditions using AddModuleScore on curated gene sets of mTOR positive regulators (*Mtor, Rptor, Mlst8, Rheb, Rraga-d, Lamtor1-5, Akt1-3, Pik3ca/cb/r1*), negative regulators (*Tsc1, Tsc2, Pten, Ddit4, Deptor, Sesn1-3, Prkaa1/2, Stk11*), and downstream targets (*Rps6kb1/2, Eif4ebp1/2, Eif4e/b, Rps6*). We found that *Lepr/Glp1r* neurons tended to have higher expression of mTOR positive regulators and lower expression of mTOR negative regulators than other *Lepr* cell types (Extended Data Figure 10), suggesting that the preserved leptin sensitivity of *Lepr/Glp1r* neurons is unlikely to arise from decreased mTOR pathway activity. We have noted this finding in the Discussion (Page 8).

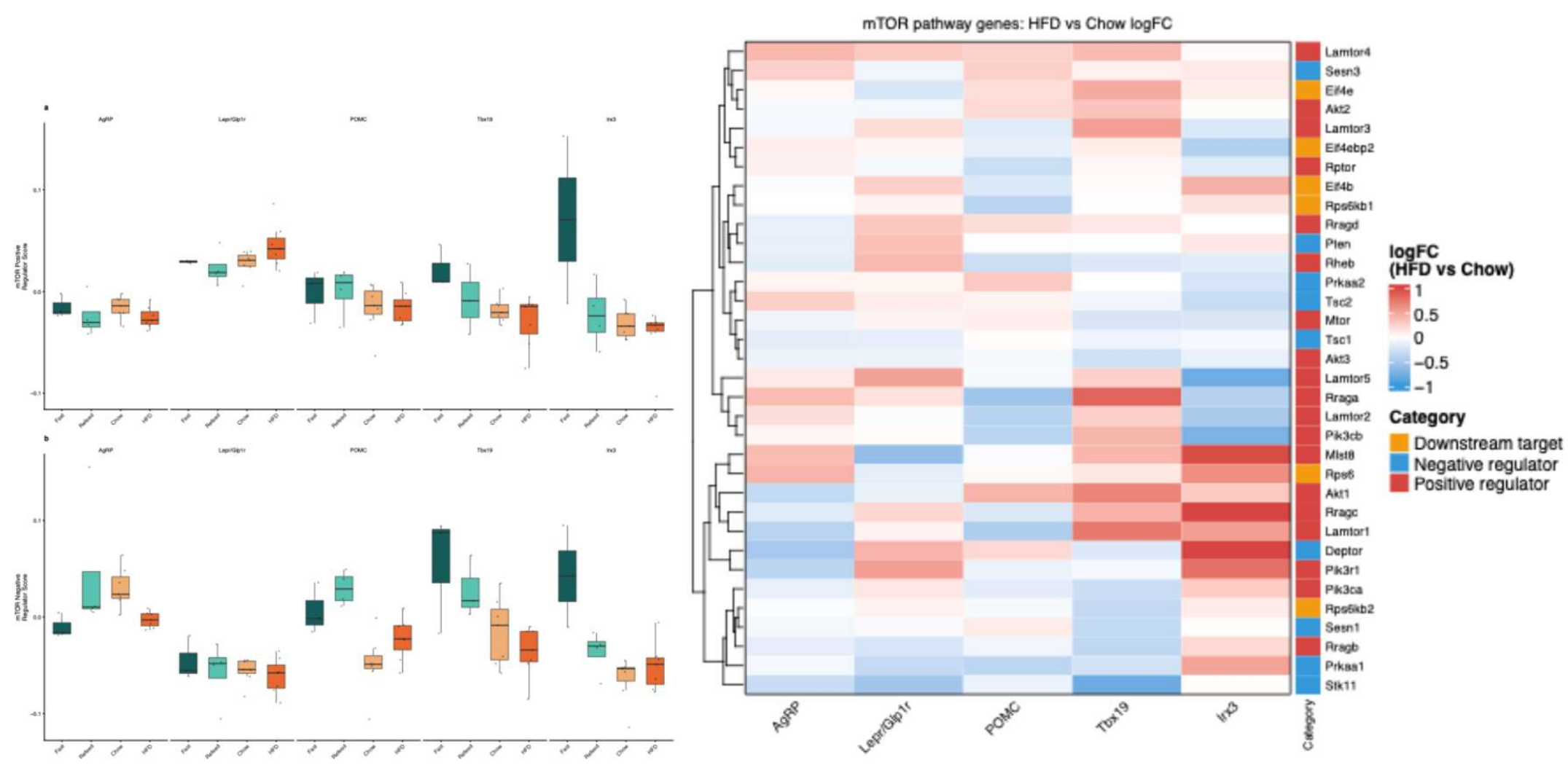

1st Editorial Decision **2nd Jul 2026**

Dear Dr Myers, dear Dr Pers,

Thank you again for the transfer of your revised manuscript (EMBOJ-2026-124542-T) to The EMBO Journal, as well as for your patience with our feedback from the arbitrating reevaluation process. We have carefully assessed your manuscript, and the point-by-point response provided to the referee concerns that were raised during peer-review at a different journal. In addition, and as mentioned before, we decided to send the revised version of your work back to seek original reviewer input with respect to technical robustness, conceptual advance and overall suitability of your work for publication in The EMBO Journal. We have now received a re-report by former referee #1 which I enclose below. Please note that while former referee #3 was at this time not able to recheck the study, we have evaluated your rebuttal response to this expert editorially and found the issues raised to be satisfactorily addressed. As you will see from the other expert #1's remarks, s/he states that the study is still consolidated by the amendments and is supportive of publication at our journal.

We are thus pleased to inform you that your study has been provisionally accepted for publication at The EMBO Journal.

We now need you to take care of a number of minor issues related to formatting and data annotation, as to the policies of our journal, which I will share shortly in a separate message, together with additional changes and requests by our production team for Source Data provision.

Please submit a revised version of the manuscript using the link enclosed below.

As you might have seen on our web page, every paper at the EMBO Journal now includes a 'Synopsis', displayed on the html and freely accessible to all readers. The synopsis includes a 'model' figure as well as 2-5 one-short-sentence bullet points that summarize the article. I would appreciate if you could provide this figure and the bullet points.

Please let me know any time should you have additional questions regarding above points.

Thank you again for giving us the chance to consider your manuscript for The EMBO Journal, I look forward to hearing from you and receiving your final revised version of the manuscript.

Kind regards,

Daniel Klimmeck

Daniel Klimmeck PhD
Senior Editor
The EMBO Journal

Please remember: Digital image enhancement is acceptable practice, as long as it accurately represents the original data and conforms to community standards. If a figure has been subjected to significant electronic manipulation, this must be noted in the figure legend or in the 'Methods' section. The editors reserve the right to request original versions of figures and the original images that were used to assemble the figure.

Referee #1:

The authors have satisfactorily addressed the concerns raised by this reviewer. They have done an excellent job in streamlining their main message throughout the manuscript. Data added for the revision further strengthen the paper. This reviewer does not have additional suggestions.

Dear Dr Myers, dear Dr Pers,

Further to below, I attach the mentioned list of formatting changes required for your final resubmission.

Please let us know any time should you have additional questions related.

Best regards,

Daniel Klimmeck

Daniel Klimmeck PhD
Senior Editor
The EMBO Journal

******

Formatting changes required for the revised version of the manuscript:

>> Authors: All corresponding authors need institutional email addresses entered into our online system and accounts need the ORCID number linked (M.G.M.). Please see below for additional information.

>> Author Contributions: Remove the author contributions information from the manuscript text. Note that CRediT has replaced the traditional author contributions section as of now because it offers a systematic machine-readable author contributions format that allows for more effective research assessment. and use the free text boxes beneath each contributing author's name to add specific details on the author's contribution.

More information is available in our guide to authors.
https://www.embopress.org/page/journal/14602075/authorguide

>> Provide a completed Author Checklist.

>> Adjust the title of the 'Competing Interests' section to 'Disclosure and Competing Interests Statement'.

>> Please provide the main manuscript text as .docx file with a size below 20MB.

>> Correct the order and headings of the manuscript sections to Abstract / Introduction / Results / Discussion / Methods / Data Availability / Acknowledgements / Disclosure and Competing Interests Statement / References / Figure Legends / Expanded View Figure Legends

>> Figure callouts: Please ensure the figures are called out in sequential order. Currently, Fig. 2 and Fig 3A-D are called out before Fig. 1G, etc. For the main figures, every panel needs to be cited in the manuscript text.

>> Dataset EV Legends: Rename Suppl. Tables 1, 2, 4 "Dataset EV1", "Dataset EV2", etc. Remove the legends from the manuscript text and add the corresponding legend to each EV dataset, either on a separate tab/worksheet, or by adding them to a simple readme .txt file that can be ZIPed to each dataset.

>>Remove the 'data not shown' statement on p.8, or add respective information.

>> Funding information: please add 'NNF18CC0033668' to the list of funders in our system.

>> References: please adjust reference format to EMBO Journal format, 10 authors et al.; In the text of the manuscript, a reference should be cited by author and year of publication; no more than two authors may be cited per reference; 'et al' should be used if there are more than two authors (i.e. Smith & Jones, 2003; Smith et al, 2000). In the reference list, citations should be listed in alphabetical order and then chronologically, with the authors' surnames and initials inverted; where there are more than 10 authors on a paper, 10 will be listed, followed by 'et al.'.

>> Figures in separate files: Figures should be removed from the manuscript text and uploaded as individual, high resolution figure files. Legends should be placed after the References.

>> Please add a Reagents and Tools table to the Methods section, as a separate file using the existing template in the Guide For Authors, listing key reagents, experimental models, software and relevant equipment.

Please compare also our Guide-to-Authors instructions:
https://www.embopress.org/page/journal/14602075/authorguide#structuredmethods

>> Artificial Intelligence annotation: please disclose use of AI tools in a separate section at the end of the Methods, including details on which parts of the article were assembled with the assistance of AI programs and details on the tools employed.

>> Please provide source data for the study as to the separate request e-mail. Source data should be uploaded as one (zipped) file per figure.

>> Data availability section: Provide the complete set of updated hyperlinks and make sure the datasets are made publicly accessible.

>> Consider additional changes and comments from our production team as indicated below:

- Data availability section:

Please note that the accession ID for the " EMBL-EBI BioStudies database (accession codes)" is not provided in the data availability statement.

- Figure legends:
1. Please note that the legend for figure Extended Data Figure 9C is mislabeled as Extended Data Figure 9B; and the figure panel Extended Data Figure 9B is not provided in the manuscript. This needs to be rectified.
2. Please note that the legend for figure panel Extended data figure 7F; is missing in the manuscript. This needs to be rectified.
3. Please define the annotated p values */**/***/**** as well as provide the exact p-values for the same in the legend of figure 2D; as appropriate.
4. Please note that the exact p values are not provided in the legends of figures 4I; 5A-C,E,F,G; 6A,B,F; Extended data figure 5D; Extended Data Figure 8F,G,H,I.
5. Please indicate the statistical test used for data analysis in the legends of figure 1G; 3C; 4I,K; Extended Data Figure 5D,E; Extended Data Figure 8F,G,H,I.
6. Please note that the box plots need to be defined in terms of centre, minima, maxima, and whiskers, bounds of box and percentile in the legends of figure 2D; 4I; Extended Data Figure 3A; Extended data figure 5D.
7. Please note that information related to n is missing in the legends of figures 1C; 2C,D; 3B,C,D; 4I,K,M; 6D,F; Extended Data Figure 3A,B; Extended data figure 5D; Extended data figure 6F; Extended data figure 7F; Extended Data Figure 8F; Extended Data Figure 10A.
8. Please note that the error bars are not defined in the legends of figures 2C; 3B,D; Extended Data Figure 3B; Extended data figure 7F.

Further information is available in our Guide For Authors: https://link.springer.com/journal/44318/submission-guidelines

The authors addressed the remaining editorial issues.

1st Revision - Editorial Decision 4th Sep 2026

Dear Dr Pers, dear Dr Myers,

Thank you for submitting the revised version of your manuscript. I have now evaluated your amended manuscript and concluded that the remaining minor concerns have been sufficiently addressed.

I am thus pleased to inform you that your manuscript has been accepted for publication in the EMBO Journal.

Your manuscript will be processed for publication by EMBO Press. It will be copy edited and you will receive page proofs prior to publication. Please note that you will be contacted by Springer Nature Author Services to complete licensing and payment information.

Please note that it is The EMBO Journal policy for the transcript of the editorial process (containing referee reports and your response letter) to be published as an online supplement to each paper. If you do NOT want this, you will need to inform the Editorial Office via email immediately. More information is available here: https://www.embopress.org/transparent-process#Review_Process

You may qualify for financial assistance for your publication charges - either via a Springer Nature fully open access agreement or an EMBO initiative. Check your eligibility: https://www.embopress.org/page/journal/14602075/authorguide#chargesguide

Should you be planning a Press Release on your article, please notify the production team via this form in order to coordinate publication and release dates: https://forms.cloud.microsoft/e/eH4A8JVP6g

If you have any questions, please do not hesitate to contact the Editorial Office. Thank you for your contribution to The EMBO Journal.

Thank you again for this contribution to The EMBO Journal and congratulations on a successful publication! Please consider us again in the future for your most exciting work.

Best regards,

Daniel Klimmeck

Daniel Klimmeck, PhD
Senior Editor
The EMBO Journal
EMBO
Postfach 1022-40
Meyerhofstrasse 1
D-69117 Heidelberg
contact@embojournal.org

*****
Please note that it is The EMBO Journal policy for the transcript of the editorial process (containing referee reports and your response letters) to be published as an online supplement to each paper. If you should prefer removal of any referee-only figures included in the point-by-point response(s), e.g. because they may still be used for future publication or because they have been reproduced from published work by others, please do let us know immediately via response email.

More information is available here: https://link.springer.com/partners/embo-press/editorial-policies#Peer%20review

** Click here to be directed to your login page: https://emboj.msubmit.net

EMBO Press Author Checklist

Corresponding Author Name: Myers and Pers	
Journal Submitted to: EMBO	
Manuscript Number: EMBOJ-2026-124542	

USEFUL LINKS FOR COMPLETING THIS FORM

The EMBO Journal - Author Guidelines
EMBO Reports - Author Guidelines
Molecular Systems Biology - Author Guidelines
EMBO Molecular Medicine - Author Guidelines

Repor
This
checkl
Please note that a copy of this checklist will be published alongside your article.

Abridged guidelines for figures
1. Data
The data shown in figures should satisfy the following conditions:

- the data were obtained and processed according to the field's best practice and are presented to reflect the results of the
- ideally, figure panels should include only measurements that
- plots include clearly labeled error bars for independent
- if n<5, the individual data points from each experiment should
- Source Data should be included to report the data underlying

2. Captions
Each
figure

- a specification of the experimental system investigated (eg cell
- the assay(s) and method(s) used to carry out the reported
- an explicit mention of the biological and chemical entity(ies)
- an explicit mention of the biological and chemical entity(ies)
- the exact sample size (n) for each experimental
- a description of the sample collection allowing the reader to understand whether the samples represent technical or
- a statement of how many times the experiment shown was
- definitions of statistical methods and measures:
  - common tests, such as t-test (please specify whether paired vs. unpaired), simple χ2 tests, Wilcoxon and Mann-Whitney
  - are tests one-sided or two-sided?
  - are there adjustments for multiple comparisons?
  - exact statistical test results, e.g., P values = x but not P
  - definition of 'center values' as median or average;
  - definition of error bars as s.d. or s.e.m.

Please complete ALL of the questions below.
Select "Not Applicable" only when the requested information is not relevant for your study.

Materials

Newly Created Materials	Information included in the manuscript?	In which section is the information available? (Reagents and Tools Table, Materials and Methods, Figures, Data Availability Section)
New materials and reagents need to be available; do any restrictions apply?	Not Applicable	

Antibodies	Information included in the manuscript?	In which section is the information available? (Reagents and Tools Table, Materials and Methods, Figures, Data Availability Section)
For **antibodies** provide the following information:<br>- Commercial antibodies: RRID (if possible) or supplier name, catalogue number and or/clone number<br>- Non-commercial: RRID or citation	Yes	Reagents and Tools Table

DNA and RNA sequences	Information included in the manuscript?	In which section is the information available? (Reagents and Tools Table, Materials and Methods, Figures, Data Availability Section)
**Short novel DNA or RNA including primers, probes**: provide the sequences.	Not Applicable	

Cell materials	Information included in the manuscript?	In which section is the information available? (Reagents and Tools Table, Materials and Methods, Figures, Data Availability Section)
**Cell lines:** Provide species information, strain. Provide accession number in repository **OR** supplier name, catalog number, clone number, and/**OR** RRID.	Not Applicable	
**Primary cultures:** Provide species, strain, sex of origin, genetic modification status.	Not Applicable	
Report if the cell lines were recently **authenticated** (e.g., by STR profiling) and tested for mycoplasma contamination.	Not Applicable	

Experimental animals	Information included in the manuscript?	In which section is the information available? (Reagents and Tools Table, Materials and Methods, Figures, Data Availability Section)
**Laboratory animals or Model organisms:** Provide species, strain, sex, age, genetic modification status. Provide accession number in repository **OR** supplier name, catalog number, clone number, **OR** RRID.	Yes	Reagents and Tools Table, Materials and Methods, Figures
**Animal observed in or captured from the field:** Provide species, sex, and age where possible.	Yes	Materials and Methods, Figures
Please detail **housing and husbandry conditions**.	Yes	Materials and Methods

Plants and microbes	Information included in the manuscript?	In which section is the information available? (Reagents and Tools Table, Materials and Methods, Figures, Data Availability Section)
**Plants:** provide species and strain, ecotype and cultivar where relevant, unique accession number if available, and source (including location for collected wild specimens).	Not Applicable	
**Microbes:** provide species and strain, unique accession number if available, and source.	Not Applicable	

Human research participants	Information included in the manuscript?	In which section is the information available? (Reagents and Tools Table, Materials and Methods, Figures, Data Availability Section)
If collected and within the bounds of privacy constraints report on age, sex and gender or ethnicity for all study participants.	Not Applicable	

Core facilities	Information included in the manuscript?	In which section is the information available? (Reagents and Tools Table, Materials and Methods, Figures, Data Availability Section)
If your work benefited from core facilities, was their service mentioned in the	Yes	Data Availability Section

Design

Study protocol	Information included in the manuscript?	In which section is the information available? (Reagents and Tools Table, Materials and Methods, Figures, Data Availability Section)
If study protocol has been **pre-registered, provide DOI in the manuscript**. For clinical trials, provide the trial registration number **OR** cite DOI.	Not Applicable	
Report the **clinical trial registration number** (at ClinicalTrials.gov or equivalent), where applicable.	Not Applicable	

Laboratory protocol	Information included in the manuscript?	In which section is the information available? (Reagents and Tools Table, Materials and Methods, Figures, Data Availability Section)
Provide DOI OR other citation details if **external detailed step-by-step protocols** are available.	Not Applicable	

Experimental study design and statistics	Information included in the manuscript?	In which section is the information available? (Reagents and Tools Table, Materials and Methods, Figures, Data Availability Section)
Include a statement about **sample size** estimate even if no statistical methods were used.	Yes	Materials and Methods
Were any steps taken to minimize the effects of subjective bias when allocating animals/samples to treatment (e.g. **randomization procedure**)? If yes, have they been described?	Yes	Materials and Methods, FIgures
Include a statement about **blinding** even if no blinding was done.	Yes	Materials and Methods
Describe **inclusion/exclusion criteria** if samples or animals were excluded from the analysis. Were the criteria pre-established?<br><br>If sample or data points were omitted from analysis, report if this was due to attrition or intentional exclusion and provide justification.	Not Applicable	
For every figure, are **statistical tests** justified as appropriate? Do the data meet the assumptions of the tests (e.g., normal distribution)? Describe any methods used to assess it. Is there an estimate of variation within each group of data? Is the variance similar between the groups that are being statistically compared?	Yes	Figures, Materials and Methods

Sample definition and in-laboratory replication	Information included in the manuscript?	In which section is the information available? (Reagents and Tools Table, Materials and Methods, Figures, Data Availability Section)
In the figure legends: state number of times the experiment was **replicated**	Yes	Figures
In the figure legends: define whether data describe **technical or biological**	Yes	Figures

Ethics

Ethics	Information included in the manuscript?	In which section is the information available? (Reagents and Tools Table, Materials and Methods, Figures, Data Availability Section)
Studies involving **human participants**: State details of **authority granting**	Not Applicable	
Studies involving **human participants**: Include a statement confirming that	Not Applicable	
Studies involving **human participants:** For publication of **patient photos**, include a statement confirming that consent to publish was obtained.	Not Applicable	
Studies involving experimental **animals**: State details of **authority granting ethics approva**l (IRB or equivalent committee(s), provide reference number for approval. Include a statement of compliance with ethical regulations.	Yes	Materials and Methods,
Studies involving **specimen and field samples:** State if relevant **permits** obtained, provide details of authority approving study; if none were required, explain why.	Not Applicable	

Dual Use Research of Concern (DURC)	Information included in the manuscript?	In which section is the information available? (Reagents and Tools Table, Materials and Methods, Figures, Data Availability Section)
Could your study fall under dual use research restrictions? Please check biosecurity documents and list of **select agents and toxins** (CDC): https://www.selectagents.gov/sat/list.htm	Not Applicable	
If you used a select agent, is the security level of the lab appropriate and reported in the manuscript?	Not Applicable	
If a study is subject to dual use research of concern regulations, is the name of the **authority granting approval and reference number** for the regulatory approval provided in the manuscript?	Not Applicable	

Reporting

The MDAR framework recommends adoption of discipline-specific guidelines, established and endorsed through community initiatives. Journals

Adherence to community standards	Information included in the manuscript?	In which section is the information available? (Reagents and Tools Table, Materials and Methods, Figures, Data Availability Section)
State if relevant guidelines or checklists (e.g., **ICMJE, MIBBI, ARRIVE, PRISMA**) have been followed or provided.	Not Applicable	
For **tumor marker prognostic studies,** we recommend that you follow the	Not Applicable	
For **phase II and III randomized controlled trials**, please refer to the **CONSORT** flow diagram (see link list at top right) and submit the CONSORT checklist (see link list at top right) with your submission. See author guidelines, under 'Reporting Guidelines'. Please confirm you have submitted this list.	Not Applicable	

Data Availability

Data availability	Information included in the manuscript?	In which section is the information available? (Reagents and Tools Table, Materials and Methods, Figures, Data Availability Section)
Have **primary datasets** been deposited according to the journal's guidelines (see 'Data Deposition' section) and the respective accession numbers provided in the Data Availability Section?	Yes	Data Availability Section
Were **human clinical and genomic datasets** deposited in a public access-controlled repository in accordance to ethical obligations to the patients and to the applicable consent agreement?	Not Applicable	
Are **computational models** that are central and integral to a study available without restrictions in a machine-readable form? Were the relevant accession numbers or links provided?	Yes	Materials and Methods
If publicly available data were reused, provide the respective **data citations in the reference list**.	Yes	Materials and Methods, Figures
