# Supplementary material for: Leptin-sensitive hypothalamic Lepr+/Glp1r+ neurons limit hyperphagia and weight gain in diet-induced obesity
Source: EMBO J. 2026 Oct 2;45(21):7438–67. doi: 10.1038/s44318-026-00914-w (PMC13728182; doi:10.1038/s44318-026-00914-w)
Supplement: Supplementary file 2 [file 44318_2026_914_MOESM19_ESM.pdf]

Expanded View Figures

Figure EV1. Characterization and annotation of hypothalamic cell populations by spatial transcriptomics.

(**A**) UMAP embedding of all cells from Xenium in situ sequencing annotated by Seurat cluster numbers. Colors illustrate clusters. (**B**) Representative images from Xenium in spatial context. Colors match clusters in panel a. Top image from Xenium slide 1 and bottom image from Xenium slide 2. (**C**) UMAP embeddings of all cells from Xenium in situ sequencing annotated by animal (left panel) or by diet; 15 weeks HFD (DIO) and chow (right panel). (**D**) UMAP embeddings of all cells from Xenium in situ sequencing colored by expression of *Slc17a6* (glutamatergic neurons), *Gad1* (GABAergic neurons), *Rax* (tanycytes) and *Lepr*. (**E**) UMAP embedding of all neurons from Xenium in situ sequencing colored by Seurat cluster (left panel), by Campbell et al label transfer annotations (middle panel), and by Rupp et al label transfer annotations (right panel). Spatial cell clusters correspond to the following named populations: cluster 0, *Lepr/Glp1r*; cluster 2, *Nr5a1*; cluster 4, *Agrp*; cluster 6, *Nts*; cluster 9, *Pomc*; cluster 10, *Irx3*; cluster 11, *Tbx19*.

 © The Author(s)

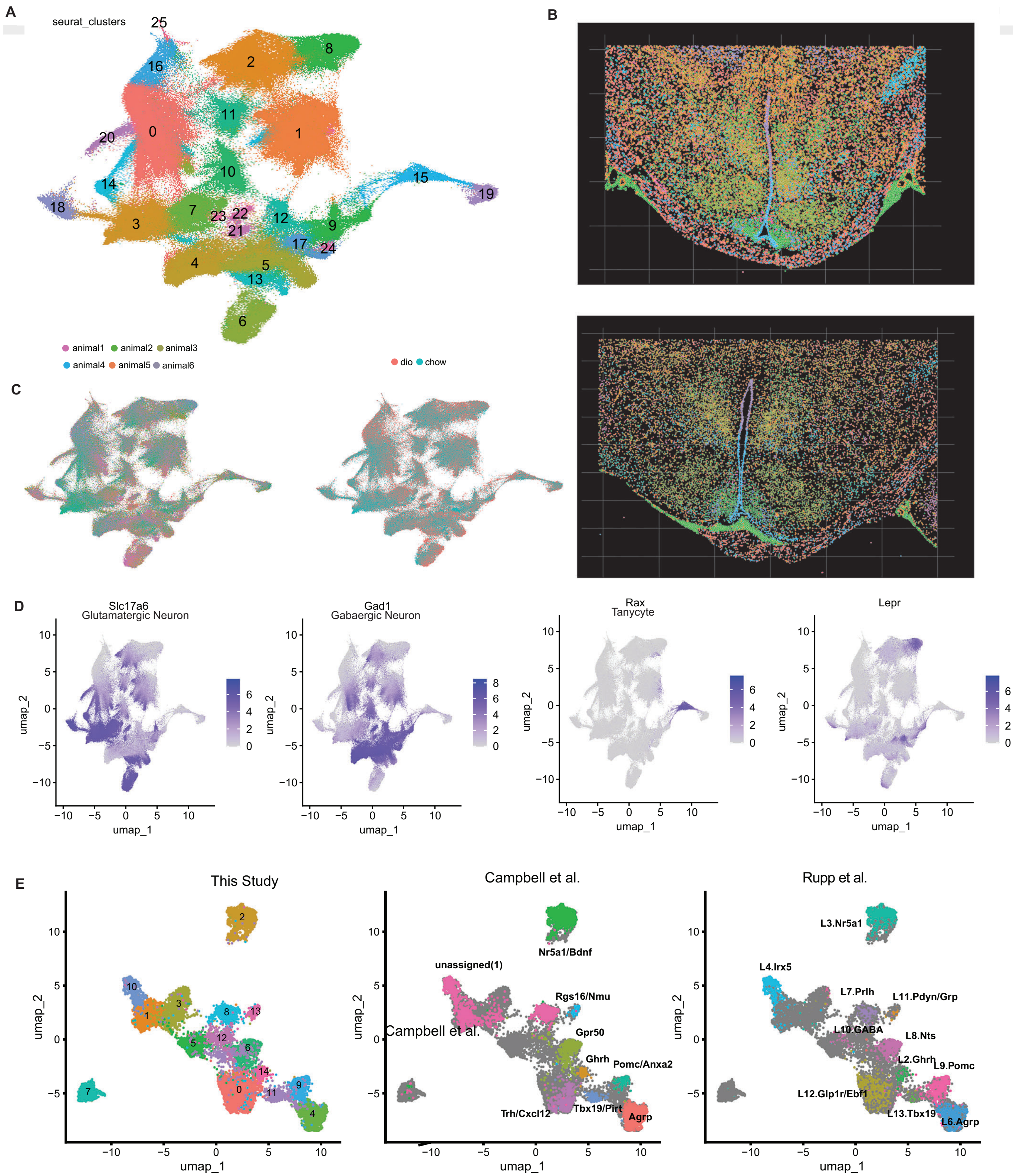
A
seurat_clusters
25
16
2
8
11
0
1
20
10
14
15
19
18
3
7
23
22
21
12
9
17
24
4
5
13
6
animal1 animal2 animal3
animal4 animal5 animal6
B
C
dio chow
D
Slc17a6
Glutamatergic Neuron
Gad1
Gabaergic Neuron
Rax
Tanycyte
Lepr
umap_1
umap_2
E
This Study
Campbell et al.
Rupp et al.
Nr5a1/Bdnf
unassigned(1)
Rgs16/Nmu
Gpr50
Ghrh
Pomc/Anxa2
Trh/Cxcl12
Tbx19/Pirt
Agrp
L3.Nr5a1
L4.Irx5
L7.Prlh
L11.Pdyn/Grp
L10.GABA
L8.Nts
L2.Ghrh
L9.Pomc
L12.Glp1r/Ebf1
L13.Tbx19
L6.Agrp

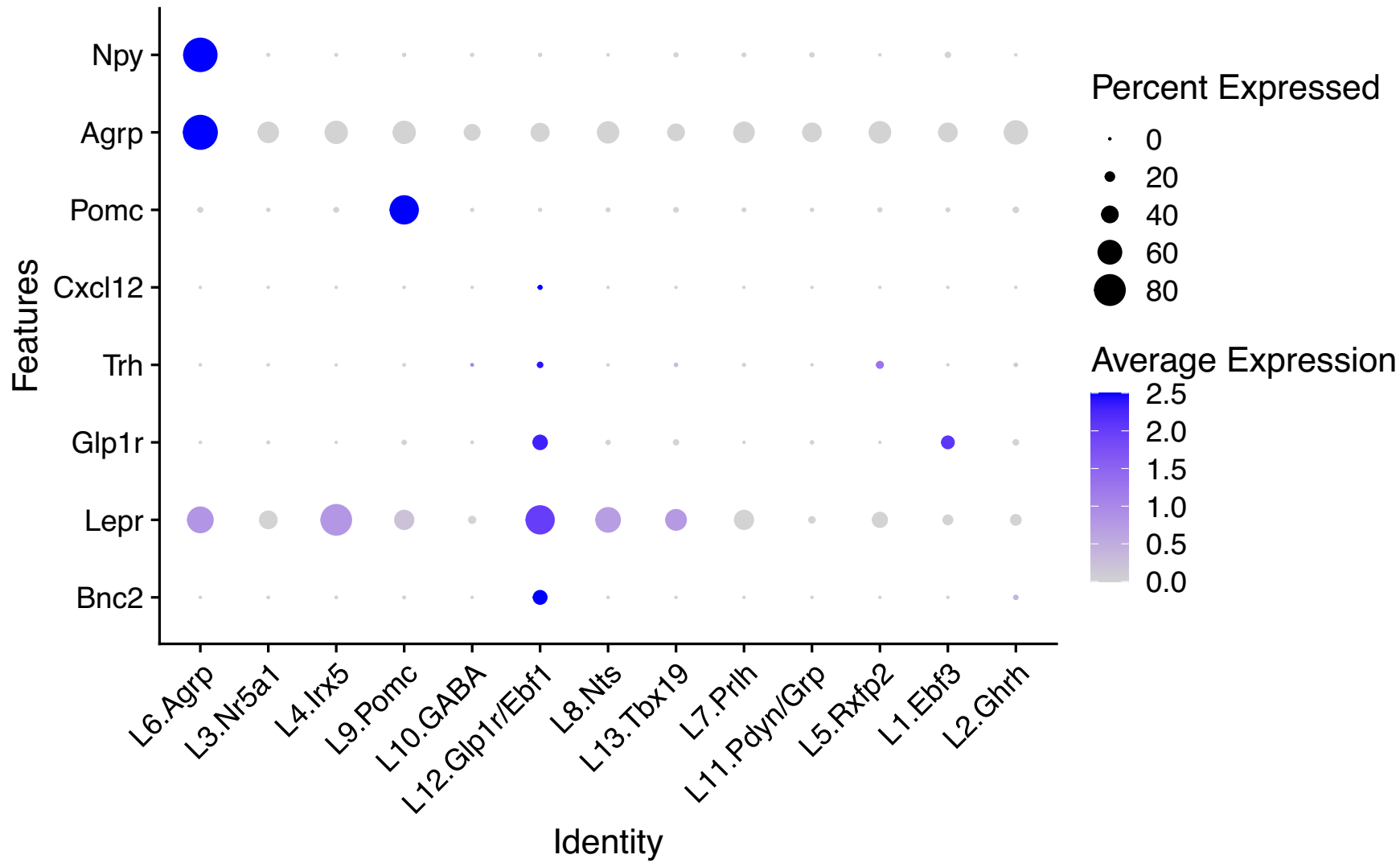

Figure EV2. Expression of *Bnc2*, *Cxcl12*, *Glp1r*, *Lepr*, and *Trh* across mediobasal hypothalamic neuronal populations identified in Rupp et al.

The canonical markers *Agrp*, *Pomc*, and *Npy* are shown for reference. The L12.Glp1r/Ebf1 cells correspond to the *Lepr/Glp1r* population. Dot size denotes the percentage of cells expressing each gene within a population, and color intensity reflects scaled average expression.

 © The Author(s)

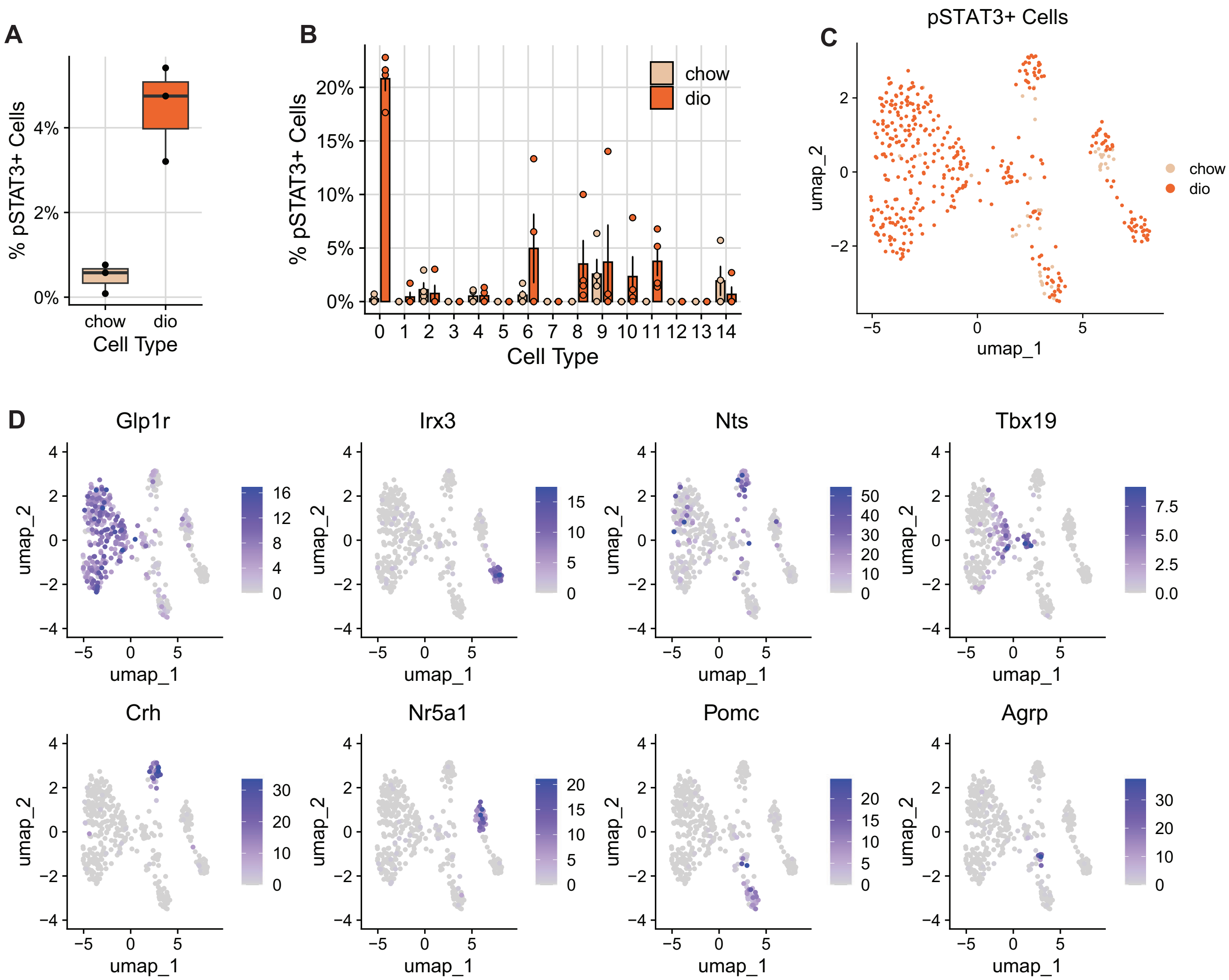

Figure EV3. Lepr/Glp1r neurons are the principal pSTAT3-immunoreactive population in diet-induced obesity.

(**A**) Quantification of pSTAT3-immunoreactive (pSTAT3-IR) cells in hypothalami of chow-fed and high-fat diet (HFD)-fed mice ($n = 3$ mice per group; 2–3 sections per mouse). (**B**) Distribution of pSTAT3-IR cells across identified neuronal clusters from Fig. 1 in HFD-fed (red) and chow-fed (light orange) mice. ($n = 3$ mice per group). Data are mean ± SEM. (**C**) UMAP visualization of Xenium in situ sequencing data showing pSTAT3-IR cells, colored by diet condition (HFD: red; chow: light orange). (**D**) UMAP visualization showing expression patterns of key neuronal marker genes (*Glp1r, Irx3, Nts, Tbx19, Crh, Nr5a1, Pomc*, and *Agrp*) in pSTAT3-IR cells.

© The Author(s)

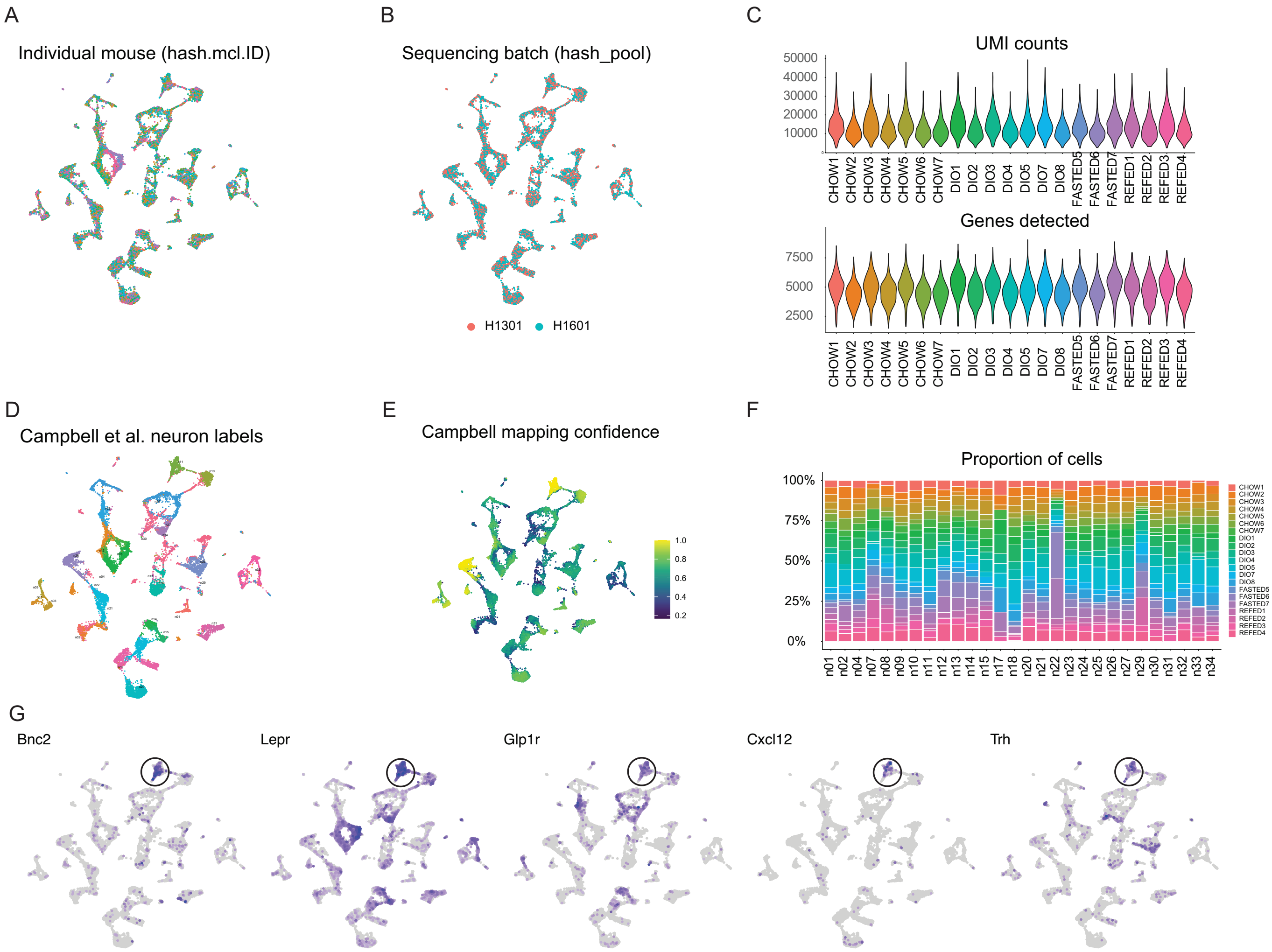

Figure EV4. Quality control and cell type annotation of mediobasal hypothalamic snRNA-seq data.

(**A**, **B**) UMAP embedding of mediobasal hypothalamic neurons colored by individual mouse (**A**) and sequencing batch (**B**). (**C**) Distribution of UMI counts (top) and genes detected (bottom) per cell across all samples (chow $n = 7$, DIO $n = 8$, fasted $n = 3$, refed $n = 4$). (**D**) UMAP colored by predicted Campbell et al neuron subtype. (**E**) Mapping confidence scores, with highest confidence in the *Lepr/Glp1r* population. (**F**) Proportion of cells assigned to each neuronal cluster (n01-n34) across individual samples, showing consistent cluster composition across mice and conditions. (**G**) UMAP colored by the expression of key markers genes of *Lepr/Glp1r* neurons to demonstrate overlap with previously described populations.

 © The Author(s)

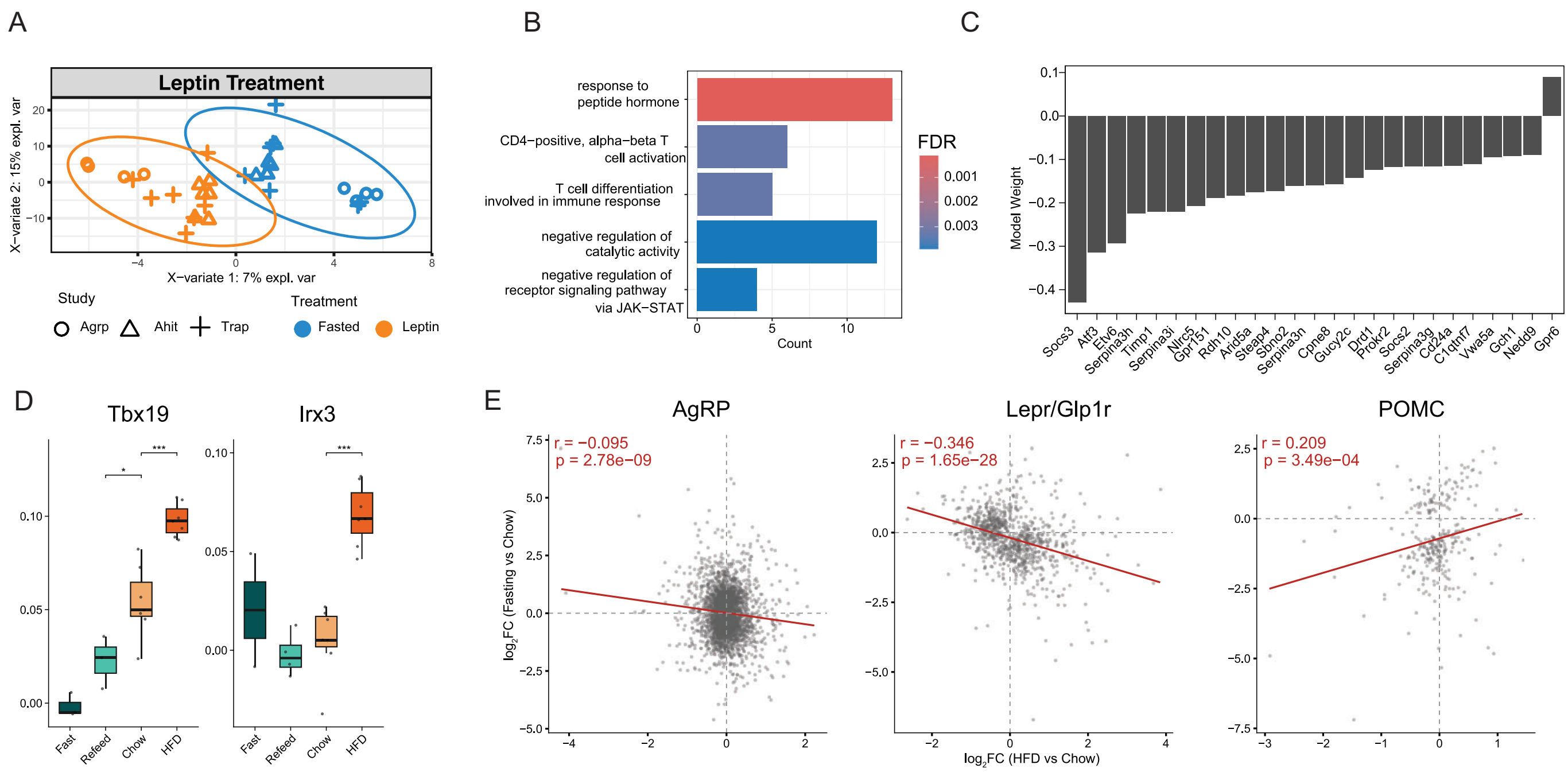

Figure EV5. Derivation and validation of the leptin gene signature (LGS) across neuron populations and nutritional states.

(**A**) MINT sPLS-DA projection of three independent leptin treatment transcriptomic datasets used to derive the leptin gene signature (LGS). Fasted/control (blue) and leptin-treated (orange) samples separate along the first two components. (**B**) Gene ontology enrichment of LGS genes, highlighting response to peptide hormone and JAK-STAT signaling among the top terms. (**C**) Model weights for individual LGS genes. (**D**) LGS expression across nutritional states in *Tbx19* and Irx3 neurons (*$P < 0.05$, **$P < 0.01$, ***$P < 0.001$; chow $n = 7$, fasted $n = 3$, refed $n = 4$, DIO $n = 8$). Box plots show the median (centre line), the 25th and 75th percentiles (box bounds) and whiskers extending to the most extreme value within 1.5× the interquartile range; points beyond the whiskers are plotted individually. (**E**) Correlation between fasting-induced (*y* axis) and DIO-induced (*x* axis) $\log_2$ fold changes in *Agrp*, *Lepr/Glp1r*, and *Pomc* neurons.

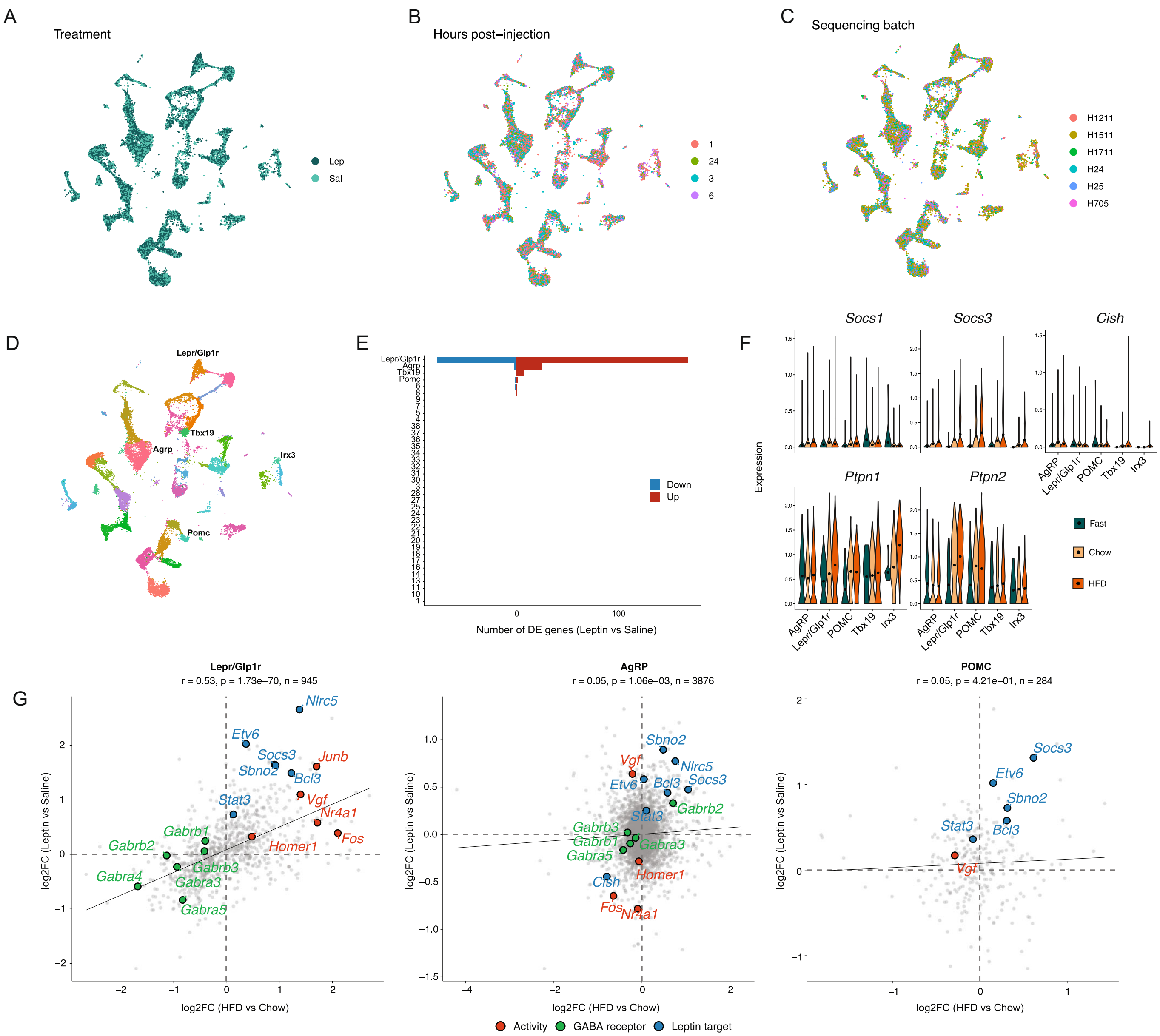

Figure EV6. Acute leptin treatment selectively engages Lepr/Glp1r neurons and recapitulates the DIO transcriptional program.

(**A–C**) UMAP embedding of mediobasal hypothalamic neurons from lean mice treated with leptin (3 mg/kg) or saline and harvested at 1, 3, 6, or 24 h post-injection ($n = 4$–6 per timepoint per group), colored by treatment (**A**), hours post-injection (**B**), and sequencing batch (**C**). (**D**) UMAP colored by neuronal cell type identity, with key *Lepr*-expressing populations labeled. (**E**) Number of differentially expressed genes (leptin vs saline; adjusted $P < 0.05$) per neuronal population. Red, upregulated; blue, downregulated. (**F**) Violin plots showing single-cell expression of JAK-STAT feedback inhibitors (*Socs1, Socs3, Cish, Ptpn1, Ptpn2*) across five *Lepr*-positive neuronal populations in fasted (teal), chow-fed (light orange), and DIO (red) conditions. Black dots indicate mean expression per group. (**G**) Scatter plots showing the relationship between HFD fold change (x axis) and leptin IP fold change (y axis) for all genes significantly regulated by diet (F test, FDR < 0.05) in *Lepr/Glp1r* (left), *Agrp* (center), and *Pomc* (right) neurons. Each point represents a gene; Pearson correlation coefficient and P value are indicated.

 © The Author(s)

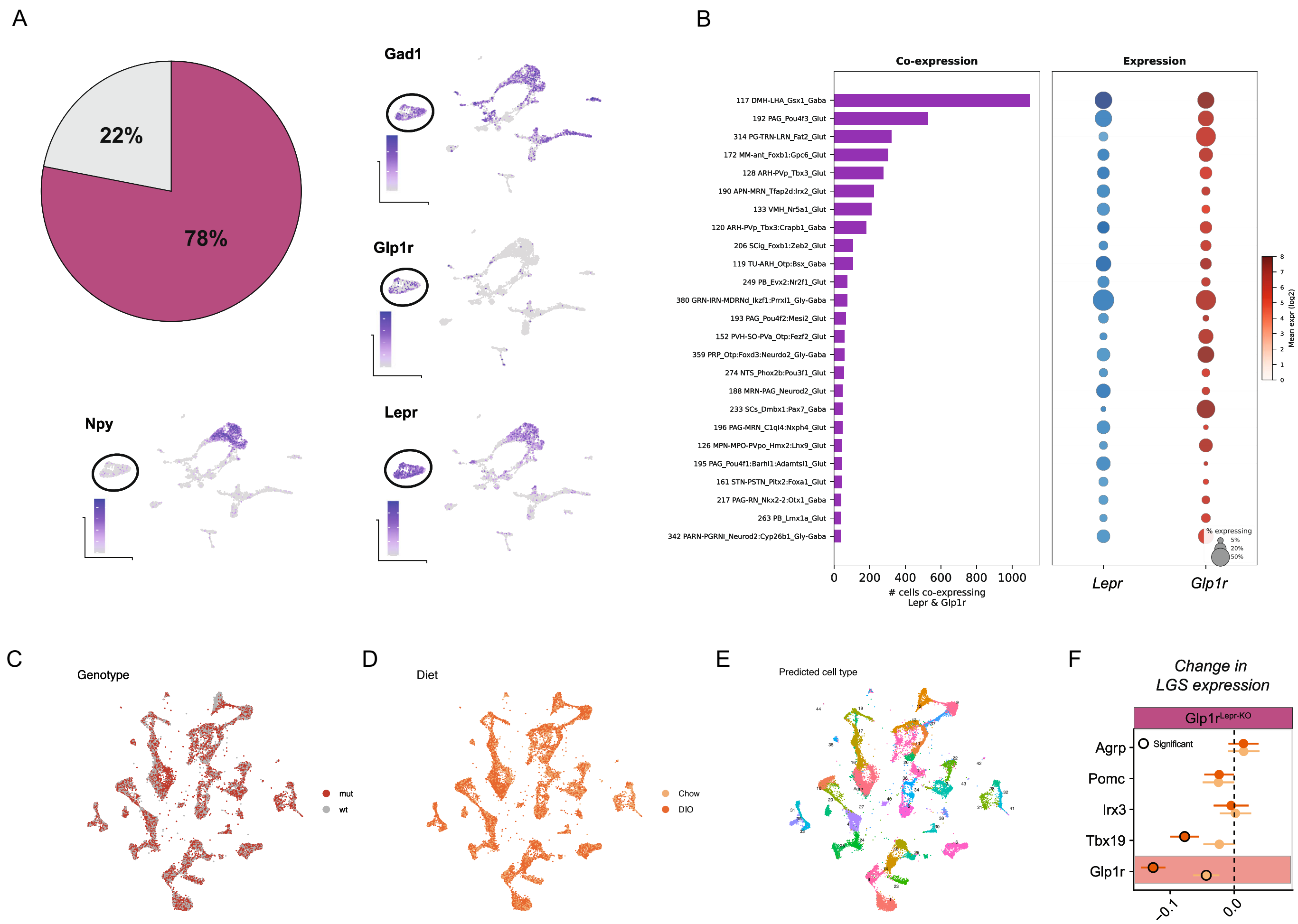

Figure EV7. Lepr/Glp1r neurons dominate inhibitory LepR input to AgRP neurons and show cell-autonomous loss of leptin signaling in Glp1rLepr-KO mice.

(**A**) *Lepr/Glp1r* neurons constitute ~78% of all *Lepr*-expressing GABAergic (*Gad1*+) input to AgRP (*Npy*+) neurons, based on reanalysis of published rabies-traced AgRP neuron afferents. (**B**) Whole-brain co-expression of Lepr and Glp1r in the Allen Brain Cell Atlas. Left, number of cells co-expressing both transcripts in the 25 cell subclasses with the highest co-expression. Right, dot plot of Lepr and Glp1r in the same subclasses: dot size denotes the percentage of cells expressing the gene and colour the mean log$_2$-normalised expression. (**C–E**) UMAP embedding of mediobasal hypothalamic neurons from $\text{Lepr}^{\text{Glp1r}}$KO and control mice, colored by (**C**) genotype, (**D**) diet (chow vs DIO; $n = 6$–7 per group), and (**E**) predicted cell type identity (41 clusters) based on label transfer from the nutritional perturbation reference atlas. (**F**) Change in leptin gene signature (LGS) expression in $\text{Lepr}^{\text{Glp1r}}$KO relative to control mice ($n = 6$–7 per group) across *Lepr* neuron subtypes (open circles denote $P < 0.05$; linear mixed-effects model). Error bars denote (95% CI).

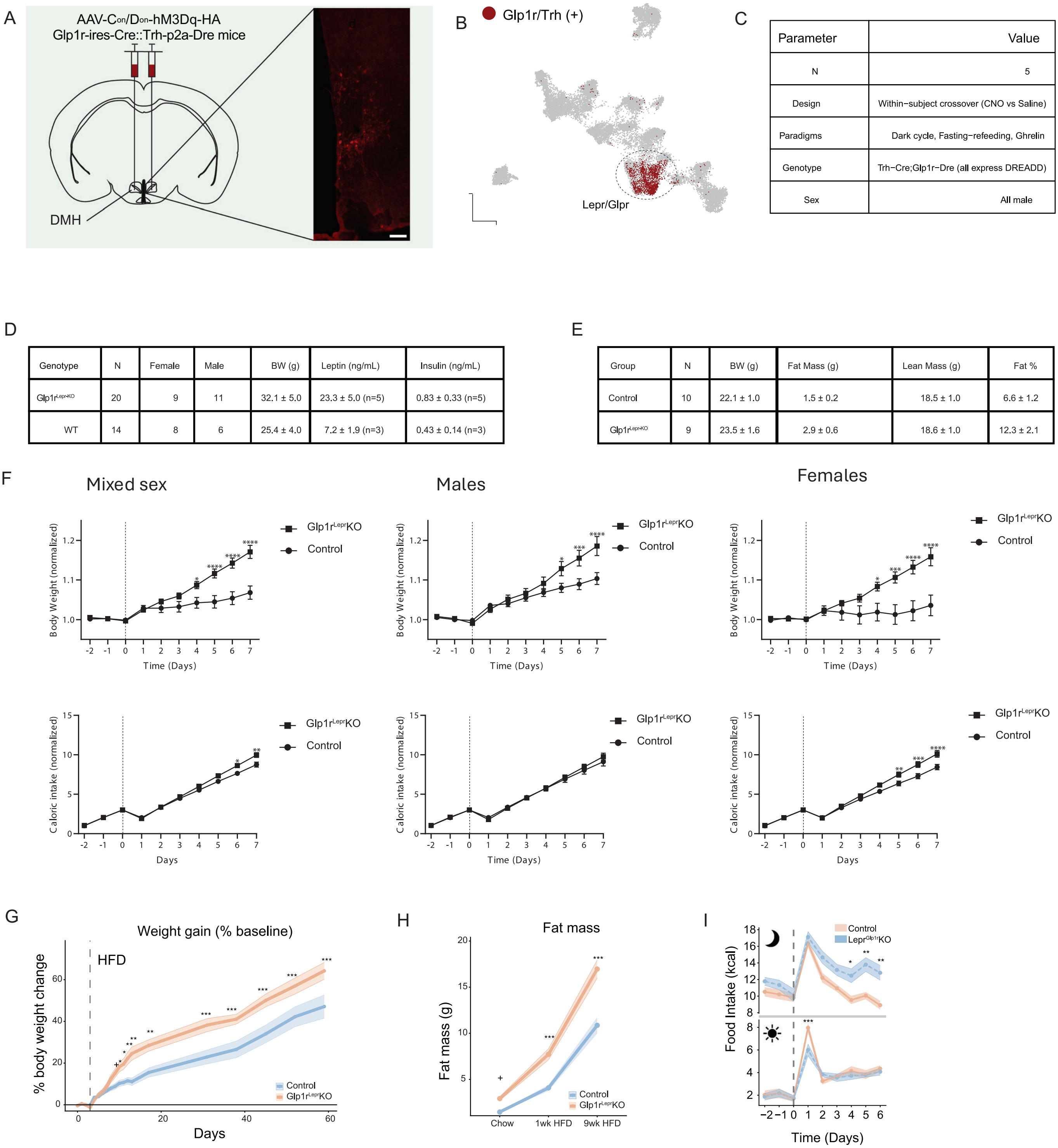

Parameter	Value
N	5
Design	Within-subject crossover (CNO vs Saline)
Paradigms	Dark cycle, Fasting-refeeding, Ghrelin
Genotype	Trh-Cre;Glp1r-Dre (all express DREADD)
Sex	All male

Genotype	N	Female	Male	BW (g)	Leptin (ng/mL)	Insulin (ng/mL)
Glp1rLeprKO	20	9	11	32.1 ± 5.0	23.3 ± 5.0 (n=5)	0.83 ± 0.33 (n=5)
WT	14	8	6	25.4 ± 4.0	7.2 ± 1.9 (n=3)	0.43 ± 0.14 (n=3)

Group	N	BW (g)	Fat Mass (g)	Lean Mass (g)	Fat %
Control	10	22.1 ± 1.0	1.5 ± 0.2	18.5 ± 1.0	6.6 ± 1.2
Glp1rLeprKO	9	23.5 ± 1.6	2.9 ± 0.6	18.6 ± 1.0	12.3 ± 2.1

Figure EV8. DREADD validation, baseline characterization, and HFD-induced weight gain and food intake in Glp1rLepr-KO mice.

(**A**) Schematic and representative HA-IR (red) image from bilateral AAV-FLEX-FREX-hM3Dq-HA injection into the caudal ARC/ventral DMH of *Glp1r*-ires-Cre;*Trh*-p2a-Dre mice. Scale bar: 200 μm. (**B**) Spatial transcriptomics reference map with projected *Trh/Glp1r* neurons (red); *Lepr/Glp1r* neuron population is curled in dashed line. (**C**) Summary of the DREADD cohort (main text Fig. 5A–C). (**D**) Baseline characteristics of chow-fed $Lepr^{Glp1r}$KO ($n = 20$) and control ($n = 14$) mice used for leptin-dependent feeding suppression experiments (main text Fig. 5D,E). (**E**) Baseline chow-period body weight and DEXA body composition of $Lepr^{Glp1r}$KO ($n = 9$) and control ($n = 10$) mice prior to HFD exposure. Data are mean ± SD. (**F**) Cumulative food intake following HFD switch. Cumulative energy intake (kcal) in control (circle) and $Lepr^{Glp1r}$KO (square) mice before and after introduction of HFD (dashed line) separated by sex. (**G**) Percent body weight change from baseline in control and $Lepr^{Glp1r}$KO mice over 60 days of HFD feeding. (**H**) Fat mass measured by ECHO-MRI at baseline (chow), 1 week, and 9 weeks of HFD feeding. (**I**) Daily food intake (kcal) in control and $Lepr^{Glp1r}$KO mice in the days immediately before and after introduction of HFD. Data are mean ± SEM. Asterisks denote BH-adjusted *P* values from within-timepoint contrasts: $^{+}P < 0.1$, $^{*}P < 0.05$, $^{**}P < 0.01$, $^{***}P < 0.001$.

 © The Author(s)

A

Genotype	Diet	N	Female	Male	BW (g)
KO	Chow	8	2	6	24.6 ± 3.2
KO	DIO	5	0	5	53.2 ± 2.8
WT	Chow	6	1	5	21.1 ± 3.0
WT	DIO	5	0	5	56.0 ± 3.6

B

Genotype	N	Female	Male	BW (g)	Leptin (ng/mL)
KO	12	0	12	48.0 ± 4.7	84.0 ± 5.5 (n=4)
WT	11	0	11	43.3 ± 12.3	78.2 ± 5.2 (n=3)

Figure EV9. Baseline characteristics and plasma leptin levels of Glp1rLepr-KO and control mice used in fasting-refeeding experiments

(**A**) Baseline characteristics of $Lepr^{Glp1r}$KO and control mice used in the chow vs DIO fasting-refeeding experiment (main text Fig. 6A). Chow-fed and DIO cohorts are independent groups of animals. (**B**) Summary table including plasma leptin for a subset of mice (KO $n = 4$, WT $n = 3$). Data are mean ± SD (tables) or mean ± SEM (dot plots) with individual animals shown.

© The Author(s)

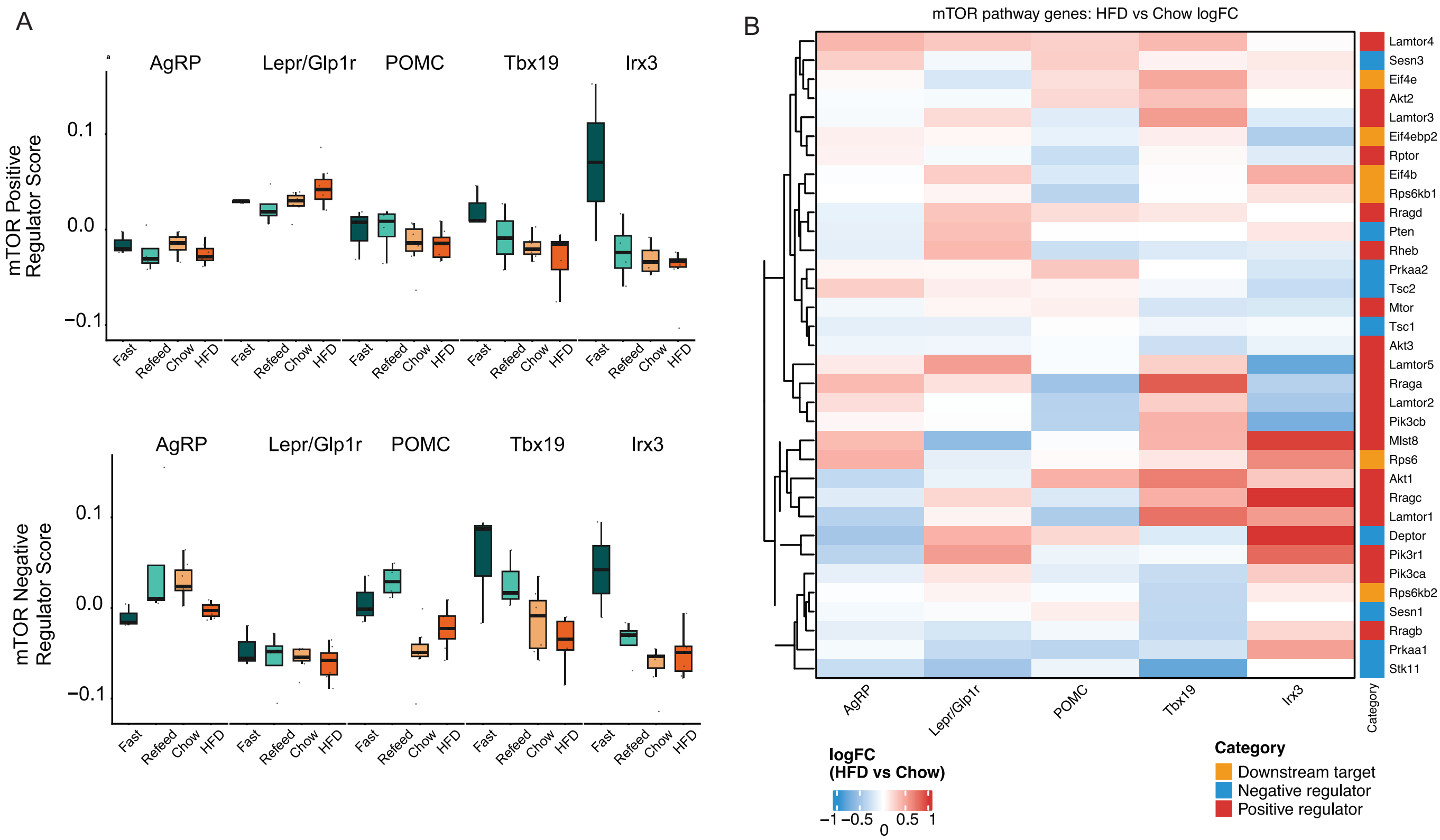

Figure EV10. mTOR pathway gene expression across Lepr neuron populations does not explain diff erential leptin sensitivity in DIO.

(**A**) Expression levels of selected mTOR pathway positive regulators (top) and negative regulators (bottom) across hypothalamic *Lepr* neuron populations from snRNA-seq data. *Lepr/Glp1r* neurons tend to express higher levels of mTOR-activating genes and lower levels of mTOR-inhibiting genes relative to other *Lepr* populations. Box plots show median, interquartile range, and whiskers extending to 1.5×IQR. (**B**) Heatmap of $\log_2$ fold-change (HFD vs Chow) for mTOR pathway genes across *Lepr* neuron populations. Genes are annotated by functional category: positive regulators (red), negative regulators (blue), and downstream targets (yellow) of mTOR signaling. Despite elevated baseline mTOR pathway gene expression, *Lepr/Glp1r* neurons retain robust leptin sensitivity in DIO, arguing against a simple model in which mTOR hyperactivation uniformly drives leptin resistance across all *Lepr* populations.

 © The Author(s)
